# Global, regional, and national burdens of ischemic heart disease in the older adults aged 60–89 years: a systematic analysis for the Global Burden of Disease Study 2019

**DOI:** 10.3389/fcvm.2025.1443881

**Published:** 2025-03-20

**Authors:** Hao Zhi, Yuedong Yang, Juan Zhao, Chenhan Mao, Jianping Shen, Xindong Wang

**Affiliations:** ^1^ The Third Clinical Medical College, Nanjing University of Chinese Medicine, Nanjing, Jiangsu, China; ^2^ Affiliated Hospital of Integrated Traditional Chinese and Western Medicine, Nanjing University of Chinese Medicine, Nanjing, Jiangsu, China; ^3^ Nanjing Lishui District Hospital of Traditional Chinese Medicine, Nanjing, Jiangsu, China

**Keywords:** global burden of disease, older adults, ischemic heart disease, disability-adjusted life-year, mortality, prevalence

## Abstract

**Background:**

Ischemic heart disease (IHD) places a heavy burden on individual and public health. Nevertheless, comprehensive assessments of the burden of IHD in the elderly are absent. It is imperative to update the burden of IHD in older adults and predict the trends.

**Methods:**

The absolute numbers and age-standardized rates (ASRs) of prevalence, mortality, and disability-adjusted life-years (DALYs) for IHD among people aged 60–89 years from 1990 to 2019 were analyzed based on the Global Burden of Disease Study 2019 (GBD 2019). Joinpoint regression analysis was utilized to evaluate the epidemiologic trend of IHD in the elderly from 1990 to 2019. Bayesian age-period-cohort model was used to predict the burden of IHD among the elderly from 2020 to 2034.

**Results:**

Age-standardized prevalence rate (ASPR), age-standardized incidence rate (ASIR), age-standardized DALY rate (ASDR), and age-standardized mortality rate (ASMR) of IHD in older adults have declined slightly over the past 30 years. In 2019, the ASPR, ASIR, ASDR, and ASMR among the elderly with IHD were 14,280.53 (95% UI, 12,301.34–16,610.6), 1,445.21 (1,142–1,793.58), 11,225.74 (10,342.09–11,960.64), and 675.24 (614.21–721.75) per 100,000**.** The burden of IHD was significantly higher in older men than in women during the study period. In terms of socio-demographic index (SDI), countries and territories with lower SDI bore a more severe burden of IHD. The burden of IHD in the elderly varied considerably across countries. Uzbekistan had the largest increase in rates of prevalence, incidence, DALY, and mortality. The projections show a downward trend in DALY and mortality rates for IHD in older adults from 2020 to 2034, but incidence and prevalence will increase.

**Conclusion:**

From 1990 to 2019, the worldwide burden of IHD among the elderly witnessed a decline. The IHD burden varied significantly across countries and territories. Policymakers should rationalize the allocation of health resources and implement effective prevention and treatment strategies to reduce the burden of IHD among the elderly in economically less developed countries and regions.

## Introduction

Ischemic heart disease (IHD) is an ischemic disease of the myocardium caused by narrowing or blockage of the coronary arteries, which is likely to result in angina pectoris, myocardial infarction, and even sudden death (1). IHD is one of the major contributors to the global burden of disease, and remains a serious public health issue.

The Global Burden of Disease Study 2019 (GBD 2019) is a large database researched by a multinational collaboration that enables us to gain a comprehensive understanding of the epidemiologic dynamics of IHD. To our knowledge, several studies have reported on the disease burden of IHD since the GBD 2019 data update (2, 3). It is estimated that the total number of IHD-related deaths reached 9.14 million in 2019, accounting for 49.2% of all cardiovascular disease-related deaths (4). As is well known, IHD occurs predominantly in the elderly population. It is essential to understand the global burden of IHD in the elderly. However, no study has focused on a comprehensive description of the global burden of IHD in older adults and its changing trends. As population aging further worsens and epidemiological shifts continue in each country, systematic assessment of the burden of IHD in older adults contributes to better policy formulation and rational allocation of healthcare resources by policymakers. Here, we report the prevalence, mortality, and disability-adjusted life-years (DALYs) of IHD in older adults aged 60–89 years between 1990 and 2019 at the global, regional, and national levels. In addition, we assessed temporal trends in the burden of IHD among older adults over the past 30 years. Overall, these results might provide important guidance for developing effective strategies to reduce the IHD disease burden.

## Materials and methods

### Data sources

Older adults aged 60–89 years were included in this study. Raw material for this study was downloaded from the GBD 2019 database (https://vizhub.healthdata.org/gbd-results/). The GBD 2019 database systematically assesses the burden of 369 diseases and injuries and 87 risk factors in 204 countries from 1990 to 2019 (5). Detailed descriptions of the raw data and general methods of the GBD 2019 study have been described in previous publications (4–7). According to the Sociodemographic Index (SDI), various countries and territories are categorized into five groups in ascending order: low SDI, low-middle SDI, middle SDI, high-middle SDI, and high SDI. In the GBD 2019 study, IHD was defined by standard case definitions, including acute myocardial infarction, chronic stable angina, chronic IHD, and IHD-induced heart failure (4). In this study, data on IHD stratified by sex, age group (60–64, 65–69, 70–74, 75–79, 80–84, and 85–89) were collected from the GBD database for global, 21 GBD regions and 204 countries and territories from 1990 to 2019. Prevalence, incidence, mortality, and DALY for IHD were extracted. Prevalence is the total number of individuals in a given population with a particular disease at a given time or for a defined period. In GBD 2019, disease prevalence rates were derived by using a broad range of population-representative data sources identified by literature review and via study collaborations, including scientific reports of cohorts and registries, population surveys, microdata from registry and cohort studies, and health system administrative data. DisMod-MR 2.1, a Bayesian meta-regression disease modeling tool, was used to estimate the prevalence of IHD. DALY, one of the summary health indicators, represents the sum of years of life lost (YLL) and years of disability life (YLD). IHD-related YLL was computed by multiplying the number of deaths in each age group by the standardized life expectancy for that age group. YLD was calculated by multiplying the prevalence of each mutually exclusive sequelae by its disability weight. The Cause of Death Ensemble model approach was utilized for modeling deaths due to IHD based on vital registration and verbal autopsy data.

### Statistical analysis

To estimate temporal trends of IHD burden in the elderly between 1990 and 2019, a joinpoint regression analysis was performed (8). The Joinpoint regression software (version 5.0.2) was used for the analyses, and results were presented as average annual percentage change (AAPC) values for age-standardized rates of prevalence, incidence, mortality, and DALY, as well as 95 confidence intervals (CIs). The trend is considered constant when 0 value is within the 95% CI of the AAPC. When both boundaries of the 95% CI are greater than 0, the trend is considered to be upward. Conversely, when both 95% CIs are negative, the trend is downward. To predict the burden of IHD among the elderly from 2020 to 2034, the Bayesian age-period-cohort model was implemented by “BAPC” package in R software (version 4.3.1) (9). R 4.3.1 was used for data analysis and visualization. *P* < 0.05 is considered statistically significant.

## Results

### Global burden

**Figure 1:**
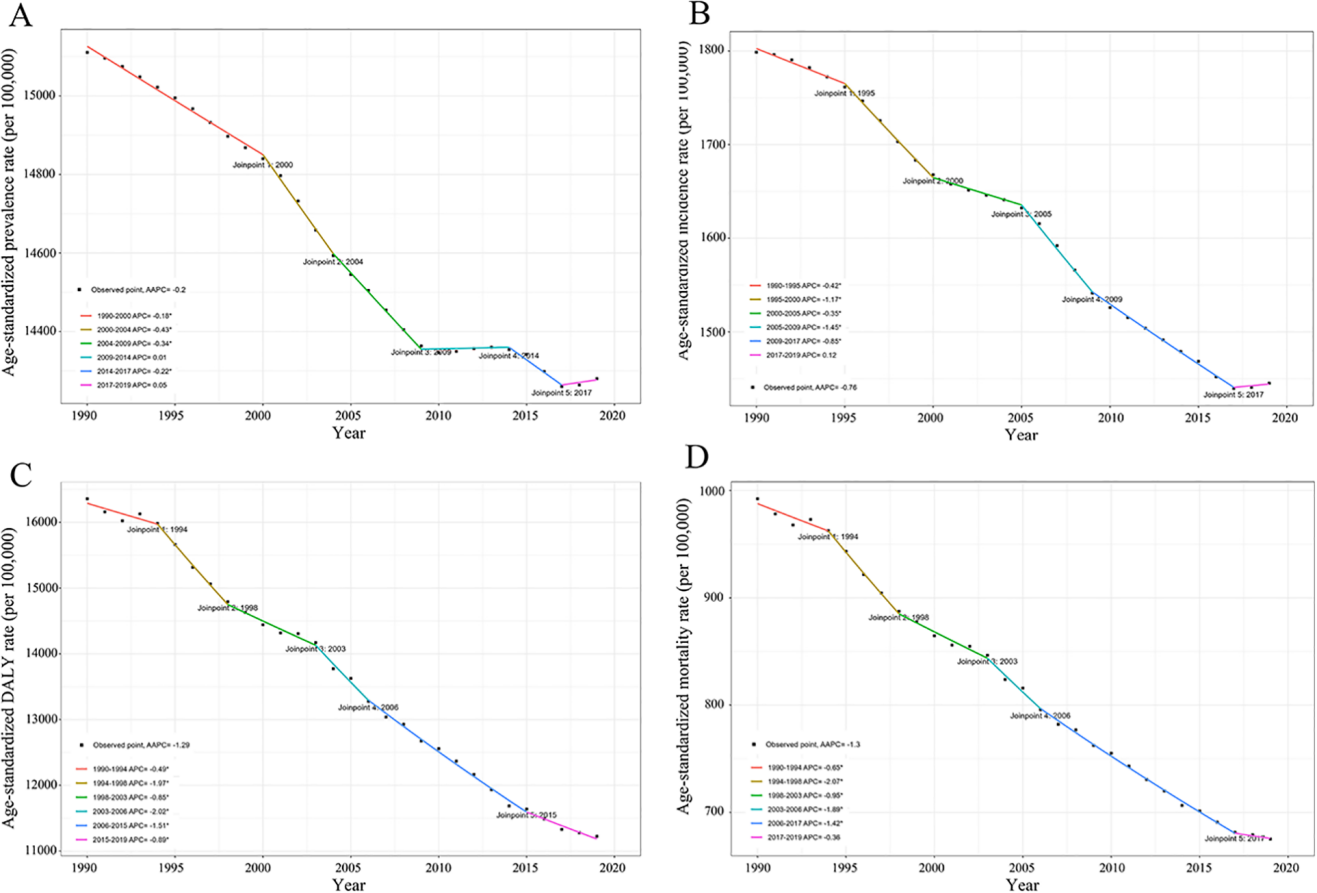
APC in IHD prevalence **(A)**, incidence **(B)**, DALY **(C)**, and mortality **(D)** per 100, 000 population among the elderly globally from 1990 to 2019. APC, annual percent change; IHD, ischemic heart disease; DALY, disability-adjusted life-years.

In 2019, there were 142,022,672 [95% uncertainty interval (UI): 125,462,924–160,840,841] cases of IHD in the elderly globally, which is more than double (105.44%) compared to 1990 (Supplementary Table 1). The age-standardized prevalence rate (ASPR) and age-standardized incidence rate (ASIR) in 2019 were 14,280.53 (95% CI: 12, 301.34–16,610.6) and 1,445.21 (1,142–1,793.58) per 100,000, respectively (Supplementary Tables 1 and 2). Over the past three decades, the ASPR [AAPC: −0.2% (95% CI: −0.22% to −0.18%)] and ASIR [AAPC: −0.76% (−0.8% to −0.72%)] for IHD among older adults have shown slightly decreasing trends. Specifically, the ASPR continued to decline between 1990 and 2019. The prevalence declined again between 2014 and 2017. The most significant decline occurred between 2000 and 2004 [APC: −0.43% (−0.5% to −0.36%)] (Supplementary Table 3 and Figure 1).

The number of DALY and death cases of IHD in older adults in 2019 was 111,169,550 (104,096,500–117,970,464) and 6,523,467 (6,021,131–6,929,986), respectively, representing an increase of 49.74% and 54.03%, respectively, compared to 1990 (Supplementary Table 4). The age-standardized DALY rate (ASDR) in 2019 was 11,225.74 (10,342.09–11,960.64) per 100,000. A continuous downward trend in ASDR has been observed over the period of 1990–2019 [AAPC: −1.29% (−1.48% to −1.1%)], with the most notable decline being observed between 2003 and 2006 [APC: −2.02% (−3.32% to −0.7%)]. Similarly, a downward trend in age-standardized mortality rates (ASMR) has been observed over the past 30 years [AAPC: −1.3% (−1.5% to −1.1%)] (Table 1). ASMR has also continued to decline from 1990 to 2017. The ASMR in 2019 was 675.24 (614.21–721.75) per 100,000. Notably, the increase in DALY and death cases attributed to IHD does not mean the condition is getting worse in the population. ASRs are the better measure compared to absolute changes in numbers (10, 11). Overall, both ASDR and ASMR have continued to decline globally, implying a gradual decline in the burden of IHD among the elderly. Progress has and continues to be made.

**Table 1:** Global and regional mortality of IHD among the elderly along with their trends from 1990 to 2019.

Death	1990		2019		1990–2019
Location	Death Cases NO.(95%UI)	ASMR/100,000 (95% CI)	Death Cases NO.(95%UI)	ASMR/100,000 (95% CI)	AAPC (95%CI)
Global	42,35,252.18 (40,25,071.17 to 43,80,609.24)	992.36 (932.27 to 1,033.38)	65,23,466.87 (60,21,130.72 to 69,29,985.81)	675.24 (614.21 to 721.75)	−1.3 (−1.5 to −1.1)
SDI					
High SDI	13,29,288.22 (12,51,356.72 to 13,66,443.19)	974.11 (915.44 to 1,005.27)	950,574.82 (860,696.18 to 10,05,966.07)	367.18 (332.6 to 389.35)	−3.3 (−3.52 to −3.08)
High-middle SDI	14,59,252.98 (13,91,617.69 to 14,98,969.41)	1,232.41 (1,163.02 to 1,275.76)	19,93,734.43 (18,20,387.15 to 21,23,635.02)	791.25 (716.71 to 847.44)	−1.55 (−1.79 to −1.31)
Middle SDI	800,813.15 (755,438.18 to 851,453.23)	821.61 (760.35 to 879.5)	20,72,038.96 (18,91,258.01 to 22,40,266.87)	784.33 (705.74 to 851.56)	−0.17 (−0.3 to −0.04)
Low-middle SDI	464,894.78 (424,933.87 to 506,229.74)	812.91 (730.69 to 895.92)	11,30,944.08 (10,22,162.42 to 12,39,165.16)	776.51 (692.69 to 857)	−0.12 (−0.17 to −0.06)
Low SDI	178,652.08 (159,558.32 to 201,257.44)	812.76 (710.29 to 923.72)	372,815.05 (331,031.36 to 417,981.36)	752.72 (655.66 to 854.99)	−0.28 (−0.34 to −0.22)
GBD regions					
Andean Latin America	13,981.57 (12,348.38 to 15,564.23)	644.71 (554.08 to 735.59)	23,575.66 (19,407.1 to 27,957.32)	362.85 (289.63 to 444.37)	−1.95 (−2.39 to −1.51)
Australasia	32,738.08 (30,798.56 to 33,749.27)	1,098.01 (1,019.65 to 1,149.54)	20,878 (18,524.75 to 22,362.96)	312.25 (271.14 to 342.86)	−4.23(−4.59 to −3.86)
Caribbean	33,471.09 (31,430.73 to 34,951.94)	1,086.22 (1,004.89 to 1,151.81)	43,965.78 (38,502.12 to 50,066.53)	706.05 (601.8 to 811.62)	−1.35 (−1.54 to −1.16)
Central Asia	97,953.59 (93,187.67 to 100,953.02)	1,950.01 (1,831.22 to 2,025.2)	151,092.54 (139,617.45 to 163,711.05)	2,288.52 (2,084.08 to 2,484.96)	0.66 (0.47 to 0.86)
Central Europe	316,016.91 (301,586.76 to 323,287.33)	1,803.88 (1,705.97 to 1,859.29)	269,855.21 (236,286.08 to 301,838.81)	917.3 (791.27 to 1,032.21)	−2.38 (−2.58 to −2.18)
Central Latin America	67,085.04 (62,907.32 to 69,732.29)	769.8 (714.68 to 805.35)	150,249 (130,778.78 to 171,668.45)	556.38 (474.27 to 639.02)	−1.18 (−1.39 to −0.96)
Central Sub-Saharan Africa	16,059.56 (13,400.44 to 19,618.5)	806.94 (630.65 to 1,028.15)	32,804.47 (25,181.87 to 42,255.9)	722.86 (525.21 to 964.13)	−0.38 (−0.52 to −0.25)
East Asia	456,505.44 (408,199.81 to 509,601.68)	561.9 (496.79 to 633.2)	15,08,053.67 (13,06,511.67 to 17,04,196.5)	664.09 (570.6 to 752.92)	0.56 (0.33 to 0.8)
Eastern Europe	640,607.78 (615,449.44 to 653,779.31)	1,949.37 (1,853.23 to 2,003.49)	759,044.62 (683,281.22 to 825,329.6)	1,692.4 (1,510.24 to 1,851.66)	−0.53 (−1.11 to 0.05)
Eastern Sub-Saharan Africa	41,306.46 (35,635.84 to 46,360.64)	598.59 (503.01 to 694.21)	84,410.11 (68,157.38 to 100,465.21)	573.52 (451.38 to 692.84)	−0.15 (−0.27 to −0.03)
High-income Asia Pacific	114,703.61 (107,042.91 to 119,650.3)	495.6 (457.57 to 520.51)	109,694.77 (92,387.96 to 119,344.97)	164.73 (139.87 to 180.43)	−3.74 (−4 to −3.47)
High-income North America	499,911.02 (468,154.64 to 515,419.63)	1,068.08 (998.54 to 1,106.9)	393,080.25 (361,216.71 to 413,956.61)	485.05 (442.92 to 513.16)	−2.7 (−2.86 to −2.53)
North Africa and Middle East	307,232.53 (284,795.85 to 329,412.74)	1,834.2 (1,667.06 to 1,993.4)	558,903.56 (497,289.04 to 627,447.12)	1,305.27 (1,142.08 to 1,470.38)	−1.15 (−1.42 to −0.87)
Oceania	2,657.69 (2,177.23 to 3,314.86)	991.47 (780.93 to 1,261.64)	6,702.2 (5,421.15 to 8,282.92)	1,084.8 (861.8 to 1,367.4)	0.3 (0.22 to 0.37)
South Asia	471,636.58 (418,776.93 to 523,699.33)	906.69 (793.95 to 1,021.92)	12,51,317.43 (10,96,656.46 to 14,04,186.47)	834.05 (722.2 to 942.46)	−0.35 (−0.46 to −0.23)
Southeast Asia	1,64,626.69 (1,50,101.19 to 1,79,943.64)	673.89 (599.69 to 749.56)	3,98,054.83 (3,57,986.09 to 4,35,028.59)	645.43 (570.45 to 714.71)	−0.13 (−0.26 to 0)
Southern Latin America	51,739.71 (48,984.7 to 53,488.36)	953.44 (881.31 to 1,008.6)	43,966.07 (40,444.45 to 46,716.06)	412.72 (369.08 to 450.22)	−2.83 (−3.08 to −2.57)
Southern Sub-Saharan Africa	14,413.71 (12,808.24 to 15,745.47)	504.26 (439.59 to 564.77)	31,484.11 (28,660.71 to 34,374.78)	544.67 (483.96 to 603.62)	0.25 (−0.22 to 0.73)
Tropical Latin America	82,808.84 (78,364.75 to 85,840.79)	885.85 (821.73 to 930.06)	1,21,302.03 (1,10,785.6 to 1,27,951.63)	431.3 (388.34 to 461.55)	−2.45 (−2.54 to −2.36)
Western Europe	7,43,561.21 (7,02,319.8 to 7,64,731.59)	941.91 (887.62 to 972.86)	4,43,555.77 (3,99,651.29 to 4,70,343.18)	333.44 (301.42 to 355.47)	−3.55 (−3.79 to −3.32)
Western Sub-Saharan Africa	66,235.05 (55,702.4 to 80,885.68)	787.61 (653.96 to 967.66)	1,21,476.79 (1,02,532.48 to 1,40,065.82)	713.99 (591.75 to 830.6)	−0.33 (−0.47 to −0.18)

IHD, ischemic heart disease; ASMR, age-standardized mortality rate; UI, uncertainty interval; CI, confidence interval.

In terms of gender, the burden of IHD for both older men and women showed a decreasing trend from 1990 to 2019. Interestingly, we found that the disease burden of IHD was significantly higher in older men than in women. The ASPR was 17,798.76 (15,441.72–20,531.28) per 100,000 and 11,298.2 (9,634.61–13,286.01) per 100,000 in 2019 for both men and women, respectively. Both ASDR and ASMR were also significantly higher for males than for females. The ASDR and ASMR for males in 2019 were 13,901.52 (12,796.89–14,915.73) per 100,000 and 819.61 (749.26–879.72) per 100,000, respectively. For females, the ASDR and ASMR were 8,919.9 (7,974.97–9,655.32) per 100,000 and 556.8 (489.54–605.56) per 100,000, respectively.

### Global burden by socio-demographic index

In 2019, the low-middle SDI region recorded the highest ASPR [18,612.16 (16,089.56–21,552.61) per 100,000] and ASIR [1,957.08 (1,540.95–2,428.56) per 100,000]. While the high SDI region showed the lowest ASPR [9,649.98 (8,467.36–10,960.87) per 100,000] and ASIR [990.65 (785.57–1,224.39) per 100,000]. As for ASDR, the low SDI region [13,051.37 (11,438.54–14,744.33) per 100,000] was the highest in 2019. ASDR [6,014.48 (5,567.25–6,341.03) per 100,000] and ASMR [367.18 (332.6–389.35) per 100,000] were lowest in the high SDI regions. The greatest increase in IHD prevalence between 1990 and 2019 was in the middle SDI and low-middle SDI regions. High-SDI areas had the largest decreases in ASPR, ASIR, ASDR, and ASMR during this period.

### Continental level

Among the four continents, in 2019, the ASIRs of IHD in the elderly were ranked as follows: Africa [2,774.84 (2,135.87–3,562.52) per 100,000], Asia [2,157.67 (1,497.66–2,990.45) per 100,000], Europe [1,857.83 (1,370.77–2,449.23) per 100,000], and America [1,071.49 (782.03–1,429.26) per 100,000]. Similarly, the ASPRs in Africa, Asia, Europe, and America were 20,926.28 (18,491.06–23,694.25), 18,617.15 (15,484.73–22,233.65), 15,845.89 (13,616.91–18,419.32), and 10,581.77 (8,899.17–12,532.70) per 100,000, respectively. Among the four continents, Africa had the highest ASDR and ASMR, which were 14,908.37 (13,645.48–16,209.31) and 848.52 (774.05–922.63) per 100,000, respectively. America had the lowest ASDR and ASMR, which were 7,982.64 (7,410.07–8,311.26) and 450.59 (409.92–471.52) per 100,000, respectively.

### Regional burden

Among the 21 GBD regions, North Africa and Middle East had the highest ASPR [29,803.12 (26,926.91–32,986.1) per 100,000] in 2019. Central Asia recorded the highest ASDR [36,728.89 (33,508.6–40,063.49) per 100,000] and ASMR [2,288.52 (2,084.08–2,484.96) per 100,000]. The ASPR for Andean Latin America, Southeast Asia and Tropical Latin America has remained stable over the past 30 years. ASPRs trended downward in 11 regions, with High-income North America experiencing the most significant decline [AAPC: −1.69% (−1.76% to −1.63%)]. ASPR increased in 7 regions, with the largest increase in Western Sub-Saharan Africa [AAPC: 0.38% (0.35% to 0.4%)]. ASDR and ASMR declined in most GBD regions, with Australasia experiencing the largest declines in ASDR [AAPC: −4.45% (−4.79% to −4.11%)] and ASMR [−4.23% (−4.59% to −3.86%)]. ASDR remained stable in Eastern Europe and Southern Sub-Saharan Africa. The ASDR and ASMR increased in Central Asia, Oceania, and East Asia, with the most pronounced rise in Central Asia.

### National burden

**Figure 2:**
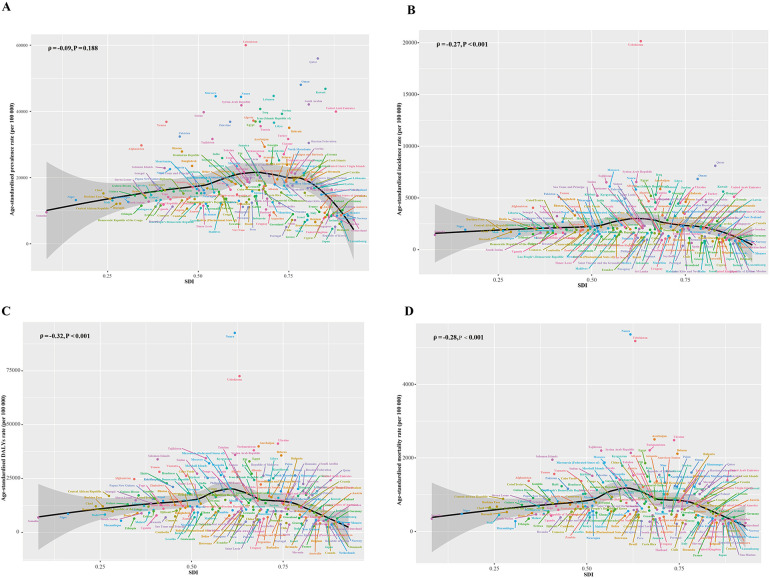
ASPR **(A)**, ASIR **(B)**, ASDR **(C)**, and ASMR **(D)** of IHD among the elderly by SDI for 204 countries and territories in 2019. The black line represents the expected values based on ASDR and SDI in all locations. Globally, ASRs for IHD in older adults declined disproportionately with increasing SDI. ASPR, age-standardized prevalence rate; ASIR, age-standardized incidence rate; ASDR, age-standardized disability-adjusted life-years rate; ASMR0, age-standardized mortality rate; IHD, ischemic heart disease; SDI, socio-demographic index; ASR, age-standardized rate. *P < 0.05; APC, annual percentage change.

At the national level, ASIR, ASDR, and ASMR for IHD disproportionately declined among older adults in 2019 as SDI increased (Figure 2). The ASPR per 100,000 population in 2019 ranged from 5,398.69 (95% CI: 4,728.13–6,158.8) in Republic of Korea to 37,507.51 (32,987.29–42,547.34) in Iran (Supplementary Table 5). The ASDR varied by more than 26 times across countries. The ASMR varied by more than 28 times. Japan showed the lowest ASDR [2,602.06 (2,301.07–2,793.51) per 100,000] and ASMR [159.71 (135.81–73.68) per 100,000] (Supplementary Tables 6 and 7). Uzbekistan recorded the highest ASIR [6,431.94 (5,753.17–7,146.19)] (Supplementary Table 8), ASDR [68, 521.23 (60,346.77–76,953.89) per 100,000], and ASMR [4,527.55 (4,029.27–5,028.11) per 100,000].

In terms of changes in ASPR, 99 countries showed a downward trend, with United States of America [AAPC: −2.1% (−2.26% to −1.94%)], Finland [−1.31% (−1.41% to −1.22%)], and Poland [−1.28% (−1.37% to −1.19%)] showing the most significant downward trend. In contrast, ASPR increased in 90 countries, with the top three countries in terms of increases being Uzbekistan [AAPC: 0.95% (0.81%–1.09%)], Guinea [0.64% (0.59%–0.69%)] and Chad [0.59% (0.55%–0.63%)]. As for ASDR, 40 countries showed upward trends and 151 countries showed downward trends. As for the changes in ASMR, 44 countries showed increasing trends and 147 countries trended downward. Denmark, Israel, and Australia had the largest decreases in ASDR and ASMR. While Uzbekistan, Tajikistan and Philippines experienced the largest increase in ASDR and ASMR.

### Prediction of IHD burden

**Figure 3:**
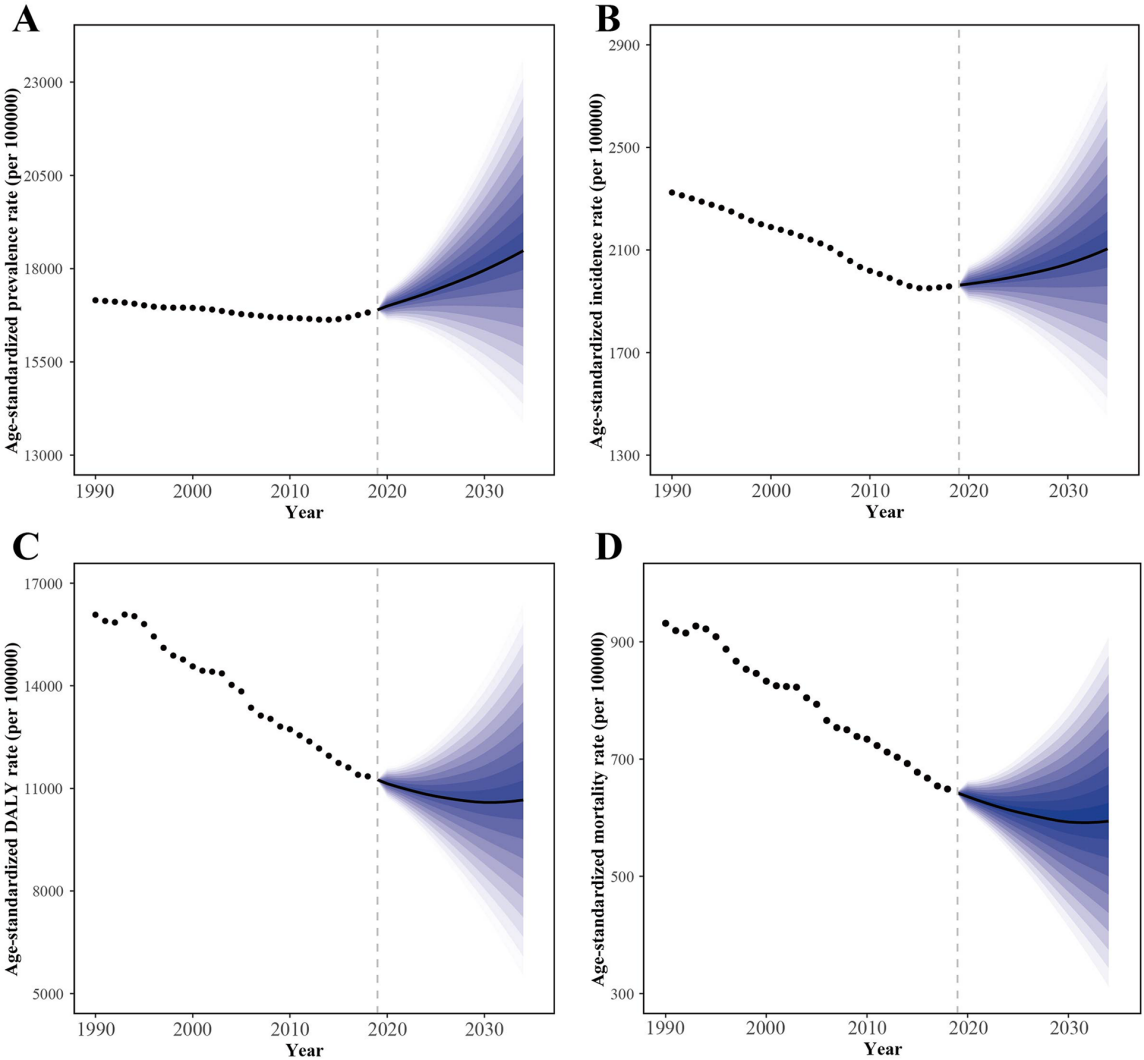
The ASR statistics and predictions for IHD among older adults based on the Bayesian age-period-cohort model. The ASPR **(A)** and ASIR **(B)** are expected to increase. The ASDR **(C)** and ASMR **(D)** of IHD are projected to show a slow decline. ASR, Age standardized rate; IHD, ischemic heart disease; ASPR, age-standardized prevalence rate; ASIR, age-standardized incidence rate; ASDR, age-standardized disability-adjusted life-years rate; ASMR, age-standardized mortality rate.

Globally, the ASPR for IHD among the elderly will continue to rise from 2020 to 2034, reaching about 18,479.8 (95% UI: 13,345.09–23,614.5) per 100,000 in 2034, higher than the 1990 level (Figure 3A). Meanwhile, the ASIR will increase slowly, reaching about 2,104.39 (95% UI: 1,378.32–2,830.47) per 100,000 by 2034, below the 1990 level (Figure 3B). In contrast, the ASDR (Figure 3C) and ASMR (Figure 3D) will continue to decline, reaching approximately 10,661.62 (4,943.95–16,379.28) and 594.04 (280.84–907.23) per 100,000, respectively, by 2034.

## Discussion

This study provides a comprehensive report on the global burden of IHD in older adults aged 60–89 years, which represents an important subgroup of the population. The results showed a gradual decline in ASPR, ASIR, ASDR, and ASMR of IHD in the elderly over the past 30 years. The absolute cases of prevalence, death, and DALY in older adults with IHD increased significantly, which may be related to global healthcare advances and population aging. Overall, older adults remain the primary target population for IHD management. The highest prevalence rates were concentrated in countries with lower SDI. Mortality and DALY were also highest in low-SDI countries. Several recent studies have reported similar results, suggesting that countries with lower levels of SDI typically have a higher burden of IHD (3, 12). In order to address the serious mortality scenarios caused by IHD, countries with relatively low SDIs should strengthen prevention strategies targeting populations at high risk of IHD and provide viable, effective, and affordable medications for the clinical management of IHD (13).

This study found that the epidemiology of IHD in the elderly varied considerably around the world. In 2019, the top three countries in the ASPR ranking were Iran, Egypt, and Kuwait. A recent study showed a decreasing trend in the incidence of IHD in Isfahan, Iran between 2006 and 2010, which was possibly attributed to the comprehensive community-based intervention called the “Isfahan Healthy Heart Program” conducted between 2001 and 2007 (14). However, there was a subsequent increase in the incidence of IHD in Isfahan between 2010 and 2016, which suggests that preventive measures must continue to be taken in order to exclude risk factors for cardiovascular diseases (14). Notably, there was a nationwide change in health monitoring policies in Iran in 2018, which likely led to a significant increase in the number of newly added cases to national cohorts. Ejection fraction was reported to be positively correlated with ankle brachial index in elderly Egyptians with IHD, respectively (15). According to the survey, there was a significant gap in the management of patients with acute heart failure in the Delta region of Egypt compared to international guidelines (16). Device therapy for heart failure has been demonstrated to be underutilized, and guideline-directed pharmacotherapy (especially beta-blockers) was underprescribed (16). Short-term mortality rate of patients with acute heart failure was significantly higher in the Delta region of Egypt compared to Western and other local registries. This was mainly attributed to the poorly resourced healthcare system in this region and the lack of a formalized heart failure management program. Worryingly, the rise in ASPR was led by Uzbekistan, Guinea and Chad. Notably, Uzbekistan had the highest ASDR and ASMR in 2019. Moreover, Uzbekistan showed the most significant rise in ASPR, ASDR and ASMR, which implies an increasing burden of IHD in older age in Uzbekistan. Therefore, countries with increasing burden of IHD in older adults, including Uzbekistan, need more resources for IHD-related preventive and curative services.

Similarly, the etiology, management, and outcomes of heart failure vary significantly between countries. A largescale study of heart failure patients from 40 countries with different economic levels showed that the most common cause of heart failure was IHD (17). Age- and sex-standardized mortality rates for heart failure were lowest in high-income countries and highest in low-income countries (17). After adjusting for patient characteristics and long-term heart failure treatment, the proportional risk of mortality within 30 days of the first admission was three to five times higher in low- and middle-income countries than in high-income countries (17). This study found that countries with lower SDI levels disproportionately bore higher IHD burdens among the elderly. Iran and Egypt, which were categorized as middle -SDI countries, showed the highest ASPR. The top three countries for both ASDR and ASMR were Uzbekistan (middle), Tajikistan (low-middle), and Azerbaijan (middle). Japan, Republic of Korea and France had the lowest ASDR and ASMR and they are all high-SDI countries. These differences highlight the importance of region-specific strategies for the prevention and management of cardiovascular disease.

Significant differences in IHD burden were observed between genders, with older men experiencing significantly higher burdens than women, which is consistent with previous research (18). Understanding the factors that determine gender differences is important for reducing the burden of IHD. Interestingly, the protective role of female hormones, known as the female advantage, has been hypothesized to explain the observed gender differences in IHD risk (19). Seemingly paradoxically, the relative risk of IHD incidence is significantly higher in smoking women compared with Asian smoking men (18). Similar findings were revealed in a meta-analysis that indicated that women who smoked had a 25% higher relative risk of coronary heart disease than men (20). Thus, tobacco control planning should take women into account, especially in countries where smoking prevalence among women is on the rise. Notably, lifestyle and environmental factors may be more prominent drivers of gender differences in IHD risk, such as smoking and stress (13, 21–23). According to the study, the burden of IHD among females in low- and middle-income countries experienced a significant increase from 1990 to 2019 in low- and middle-income countries (24). Moreover, females in several countries experienced a smaller decline in ASMR compared to males (24). Further research on gender-specific differences might facilitate improved survival in patients with IHD.

Elevated serum cholesterol concentrations, hypertension, and smoking are well recognized risk factors for IHD. Various risk factor interventions should be effectively implemented.IHD may remain asymptomatic for a long period of time until the development of diseases that potentially result in plaque destabilization or increased oxygen demand. Therefore, primary prevention is particularly important to reduce the burden of IHD. Secondary prevention is also essential to improve the prognosis and quality of life of patients. It is well known that antiplatelet and anticoagulant drugs play an essential role in the secondary prevention of IHD. Physical activity is crucial in both primary and secondary prevention of IHD. In primary prevention, physical activity is effective by controlling major cardiovascular risk factors such as hypertension and dyslipidemia. In secondary prevention, exercise reduces subsequent coronary events. No effort should be spared to encourage physical activity in at-risk asymptomatic subjects and patients with a history of IHD (25). Nonetheless, the management and prevention of IHD in low socioeconomic status populations faces numerous challenges (26). The higher mortality rate among patients with acute coronary syndromes in low-SDI countries is caused by a lack of awareness of symptoms among patients and primary care doctors, delays in access to healthcare facilities, unavailability of thrombolysis and coronary revascularization, and unaffordability of costly medications (26). Rapid diagnostic facilities and accessible and long-term care in secondary and tertiary hospitals are necessary for IHD. There is a need for a high level of attention to the social determinants of health (low educational attainment, poverty, living and working conditions), more funding for health care and effective primary care. Improved primary prevention is needed with the elimination of tobacco and trans fats, reduction of alcohol and salt consumption, and the promotion of healthy foods and physical activity. Highly effective primary care focusing on the management of blood pressure, lipids, and diabetes is desired. Sharing tasks with community health workers, electronic decision support systems, and the use of fixed-dose combinations of statins and antihypertensive drugs could significantly lower risk factors and probably contribute to a significant decrease in IHD. Finally, IHD prevention training for doctors, nurses and health workers ought to be enhanced (26).

### Limitations

There are some limitations to this study. First, the quality of the data used in this study relies on the quality control of the original GBD data collection process, and bias is still unavoidable. It is recommended that the findings of this study be further validated with the help of a large cohort study. Second, IHD is attributed to a variety of risk factors. Several key factors, such as smoking, alcohol consumption, and hypertension, have not been analyzed in this study.

## Conclusion

From 1990 to 2019, rates of IHD prevalence, incidence, mortality, and DALY among older adults declined globally. However, the IHD burden varied significantly across countries and territories. Countries with lower levels of sociodemographic development showed a more severe burden of IHD among the elderly. These data may be useful in planning ways to improve the prevention and treatment of IHD in older adults globally. Policymakers should rationalize the allocation of health resources and implement effective prevention and treatment strategies to reduce the burden of IHD among the elderly in economically less developed countries and regions.

## Data Availability

The original contributions presented in the study are included in the article/Supplementary Material, further inquiries can be directed to the corresponding authors.
