# Supplementary material for: Global, regional, and national burdens of ischemic heart disease in the older adults aged 60–89 years: a systematic analysis for the Global Burden of Disease Study 2019
Source: Front Cardiovasc Med. 2025 Mar 20;12:1443881. doi: 10.3389/fcvm.2025.1443881 (PMC11965688; doi:10.3389/fcvm.2025.1443881)
Supplement: Supplementary file 1 [file Table1.docx]

**Supplementary Table 1** Global and regional prevalence of IHD among the elderly along with their trends from 1990 to 2019.

| Prevalence | 1990 |  | 2019 |  | 1990-2019 |
| --- | --- | --- | --- | --- | --- |
| Location | Prevalenct Cases NO.(95%UI) | ASPR/100,000 (95% CI) | Prevalenct Cases NO.(95%UI) | ASPR/100,000 (95% CI) | AAPC (95%CI) |
| Global | 69131253.36 (60941732.81 to 78380851.75) | 15110.67 (12966.03 to 17599.06) | 142022672.06 (125462924 to 160840841.24) | 14280.53 (12301.34 to 16610.6) | -0.2 (-0.22 to -0.18) |
| SDI |  |  |  |  |  |
| High SDI | 18026548.46 (15938961.78 to 20335764.22) | 13271.06 (11394.35 to 15305.9) | 23580376.68 (21198132.5 to 26096046.99) | 9649.98 (8467.36 to 10960.87) | -1.1 (-1.14 to -1.06) |
| High-middle SDI | 20828162.9 (18212360.78 to 23813861.79) | 16416.72 (14001.45 to 19277.76) | 37963030.51 (33130440.23 to 43365408.61) | 14859.25 (12667.58 to 17471.53) | -0.35 (-0.36 to -0.33) |
| Middle SDI | 15618171.75 (13617510.52 to 17889504.16) | 14362.04 (12254.86 to 16921.71) | 43119722.61 (37779465.14 to 49177132) | 15113.3 (12941.92 to 17737.57) | 0.18 (0.15 to 0.2) |
| Low-middle SDI | 10867445.85 (9602795.87 to 12344145.96) | 17622.27 (15147.27 to 20465.34) | 28577373.43 (25376111.67 to 32361729.92) | 18612.16 (16089.56 to 21552.61) | 0.18 (0.15 to 0.21) |
| Low SDI | 3752208.84 (3335885.72 to 4231982.08) | 15670.84 (13477.07 to 18178.64) | 8703462.99 (7741143.87 to 9841994.84) | 16354.58 (14076.27 to 18980.96) | 0.15 (0.12 to 0.18) |
| GBD regions |  |  |  |  |  |
| Andean Latin America | 153415.63 (128270.99 to 184227.13) | 6838.77 (5508.6 to 8552.26) | 451712.57 (375453.29 to 536966.75) | 6873.63 (5539.65 to 8613.36) | 0.02 (-0.01 to 0.05) |
| Australasia | 581591.65 (533872.47 to 630650.9) | 19110.32 (17192.58 to 21066.35) | 1056633.8 (970455.86 to 1149374.83) | 16540.18 (14949.64 to 18254.86) | -0.52 (-0.61 to -0.43) |
| Caribbean | 611429.47 (551546.13 to 679412.69) | 19367.1 (17056.11 to 22054.61) | 1178955.7 (1061448.7 to 1304060.94) | 18828.68 (16604.75 to 21428.84) | -0.1 (-0.1 to -0.09) |
| Central Asia | 1282379.34 (1164740.13 to 1401567.83) | 24666.4 (21908.01 to 27753.93) | 1885840.6 (1724930.78 to 2065370.92) | 25555.26 (22887.76 to 28595.35) | 0.12 (0.1 to 0.13) |
| Central Europe | 3804697.61 (3307560.77 to 4369115.25) | 20694.98 (17475.04 to 24598.58) | 4861015.47 (4218834.44 to 5596599.47) | 16809.75 (14173.01 to 20022.3) | -0.71 (-0.75 to -0.68) |
| Central Latin America | 1118761.05 (981220.74 to 1278407.01) | 12319.13 (10485.61 to 14574.26) | 3203698.42 (2797688.12 to 3668976.58) | 11645.96 (9884.69 to 13794.99) | -0.19 (-0.21 to -0.18) |
| Central Sub-Saharan Africa | 236891.68 (211136.33 to 266361.86) | 10860.89 (9379.99 to 12599) | 507871.1 (452716.22 to 569547.36) | 10239.21 (8878.99 to 11866.02) | -0.2 (-0.21 to -0.2) |
| East Asia | 11332170.34 (9507677.11 to 13436561) | 11942.57 (9803.92 to 14607.3) | 31890474.59 (27009608.99 to 37359791.57) | 12745.31 (10524.21 to 15427.11) | 0.22 (0.17 to 0.27) |
| Eastern Europe | 7625642.65 (6731904.77 to 8670704.37) | 22217.38 (18967.4 to 26009.8) | 10223831.42 (9037105.26 to 11604065.94) | 22811.33 (19478.8 to 26665.39) | 0.09 (0.06 to 0.12) |
| Eastern Sub-Saharan Africa | 843953.38 (729343.35 to 979251.07) | 10959.45 (9143.54 to 13235.32) | 1873624.26 (1621627.26 to 2171635.91) | 11497.87 (9583.24 to 13876.87) | 0.17 (0.14 to 0.19) |
| High-income Asia Pacific | 1761234.11 (1546268.4 to 1991277.21) | 7219 (6160.17 to 8421.61) | 3619582.56 (3211554.91 to 4045835.54) | 6065.69 (5229.51 to 6990.56) | -0.61 (-0.67 to -0.55) |
| High-income North America | 7027635.84 (6173536.52 to 7945585.93) | 15125.09 (12883.89 to 17562.46) | 7415289.18 (6618582.68 to 8302269.29) | 9195.59 (7960.9 to 10544.84) | -1.69 (-1.76 to -1.63) |
| North Africa and Middle East | 5599893.75 (5146764.13 to 6094484.9) | 31150.53 (28194.46 to 34455.37) | 13524574.16 (12398993.22 to 14761935.78) | 29803.12 (26926.91 to 32986.1) | -0.15 (-0.16 to -0.13) |
| Oceania | 38634.98 (33182.02 to 45381.98) | 13605.57 (11414.7 to 16347.47) | 91904.2 (79033.54 to 107795.69) | 14178.14 (11897.23 to 16997.31) | 0.14 (0.13 to 0.15) |
| South Asia | 11796897.85 (10422652.52 to 13404001.29) | 21139.52 (18097.43 to 24571.85) | 35053539.25 (30982812.08 to 39698232.75) | 22154.23 (19089.44 to 25638.59) | 0.15 (0.11 to 0.2) |
| Southeast Asia | 2550345.74 (2189410.16 to 2968107.22) | 9733.46 (8127.75 to 11716.18) | 6337288.27 (5438877.13 to 7402699.23) | 9694.74 (8077.91 to 11685.19) | -0.01 (-0.02 to 0) |
| Southern Latin America | 476039.83 (428491.95 to 525392.93) | 8409.57 (7362.74 to 9536.87) | 726989.02 (662777.22 to 796542.58) | 6875.3 (6080.16 to 7738.49) | -0.69 (-0.71 to -0.67) |
| Southern Sub-Saharan Africa | 395159.49 (346474.11 to 451150.88) | 13128.88 (11100.25 to 15540.91) | 777293.09 (682834.85 to 891225.61) | 12624.83 (10700.52 to 14882.79) | -0.13 (-0.16 to -0.1) |
| Tropical Latin America | 1007936.98 (829696.38 to 1211888.73) | 10077.81 (8058.37 to 12671.45) | 2889036.75 (2405231.42 to 3448144.94) | 10077.03 (8132.5 to 12539.9) | 0 (-0.03 to 0.03) |
| Western Europe | 9901090.27 (8622411.93 to 11215081.66) | 12854.84 (10884.19 to 14952.78) | 12276398.71 (10974663.52 to 13674304.6) | 10506.12 (9161.43 to 12021.1) | -0.7 (-0.73 to -0.66) |
| Western Sub-Saharan Africa | 985451.74 (869176.95 to 1125230.44) | 10564.7 (8943.29 to 12515.24) | 2177118.95 (1910041.81 to 2497842.31) | 11738.23 (9928.34 to 13961.81) | 0.38 (0.35 to 0.4) |

**Supplementary Table 2** Global and regional incidence of IHD among the elderly along with their trends from 1990 to 2019.

| Incidence | 1990 |  | 2019 |  | 1990-2019 |
| --- | --- | --- | --- | --- | --- |
| Location | Incidence Cases NO.(95%UI) | ASIR/100,000 (95% CI) | Incidence Cases NO.(95%UI) | ASIR/100,000 (95% CI) | AAPC (95%CI) |
| Global | 8066892.19 (7003735.78 to 9193908.15) | 1798.7 (1428.79 to 2223.46) | 14296150.96 (12412465.31 to 16319638.83) | 1445.21 (1142 to 1793.58) | -0.76 (-0.8 to -0.72) |
| Sex |  |  |  |  |  |
| Male | 4446634.71 (3849455.25 to 5095422.01) | 2271.03 (1810.74 to 2802.59) | 8142421.53 (7060019.0 to 9,306,452.86) | 1805.08 (1433.5 to 2234.57) | -0.8 (-0.83 to -0.76) |
| Female | 3620257.49 (3134147.49 to 4128899.64) | 1426.93 (1129.52 to 1767.67) | 6153729.43 (5315650.06 to 7029270.86) | 1138.56 (894.4 to 1138.56) | -0.79 (-0.84 to -0.73) |
| SDI |  |  |  |  |  |
| High | 2500829.95 (2188240.71 to 2833760.56) | 1840.96 (1476.78 to 2255.63) | 2447491.32 (2132159.72 to 2777989.5) | 990.65 (785.57 to 1224.39) | -2.13 (-2.17 to -2.09) |
| High-middle | 2393100.68 (2080855.96 to 2725145.9) | 1941.28 (1541.59 to 2398.83) | 3742326.73 (3240167.1 to 4270487.4) | 1469.4 (1158.2 to 1829.62) | -0.97 (-1.02 to -0.91) |
| Middle | 1513799.24 (1305717.97 to 1741639.74) | 1426.88 (1129.47 to 1777.86) | 4110592.76 (3570379.29 to 4694868.14) | 1464 (1157.81 to 1818.16) | 0.05 (-0.04 to 0.14) |
| Low-middle | 1224614.23 (1050553.18 to 1430059.05) | 1957.18 (1526.67 to 2454.52) | 3024681.17 (2602906.86 to 3476586.28) | 1957.08 (1540.95 to 2428.56) | 0 (-0.03 to 0.02) |
| Low | 430211.38 (364001.83 to 502105.28) | 1784.43 (1374.34 to 2269.89) | 963298.18 (823461.16 to 1120805.07) | 1799.54 (1389.95 to 2264.26) | 0.03 (0.01 to 0.04) |
| GBD regions |  |  |  |  |  |
| Andean Latin America | 10723.54 (9020.29 to 12465.55) | 484.46 (366.09 to 620.57) | 28295.56 (23697.59 to 32913.47) | 431.55 (324.95 to 554.18) | -0.47 (-0.74 to -0.19) |
| Australasia | 79970.51 (70171.12 to 90605.31) | 2636.72 (2108.17 to 3225.57) | 112059.65 (94945.62 to 129425.56) | 1764.86 (1352.8 to 2245.91) | -1.35 (-1.51 to -1.19) |
| Caribbean | 65005.32 (56077.69 to 74698.31) | 2071.16 (1621.38 to 2596.37) | 117299.88 (100364.05 to 136373.89) | 1876.79 (1440.18 to 2378.43) | -0.31 (-0.4 to -0.22) |
| Central Asia | 179222.66 (159383.88 to 200079.04) | 3458.79 (2844.8 to 4132.22) | 278960.69 (250454.55 to 307089.22) | 3901.35 (3317.2 to 4535.61) | 0.38 (0.24 to 0.52) |
| Central Europe | 411547.42 (367515.19 to 458932.05) | 2292.59 (1902.83 to 2736.4) | 404597 (361087.53 to 451081.55) | 1394.26 (1147.63 to 1669.98) | -1.8 (-2.15 to -1.44) |
| Central Latin America | 102617.02 (86433.97 to 119586.18) | 1127.91 (855.33 to 1438.12) | 274574.85 (232974.86 to 319791.35) | 996.58 (757.95 to 1273.87) | -0.44 (-0.53 to -0.35) |
| Central Sub-Saharan Africa | 28874.58 (24238.69 to 33956.17) | 1336.86 (1013.2 to 1707.77) | 62490.12 (53186.93 to 72708.96) | 1274.84 (986.54 to 1601.69) | -0.17 (-0.19 to -0.14) |
| East Asia | 800234.21 (677490.12 to 925051.91) | 933.03 (714.13 to 1193.34) | 2483025.98 (2118117.24 to 2854352.92) | 1051.93 (811.44 to 1335.37) | 0.42 (0.34 to 0.49) |
| Eastern Europe | 1057958.9 (910394.27 to 1211975.29) | 3138.37 (2450.3 to 3925.35) | 1333503.82 (1148359.36 to 1533856.86) | 2964.54 (2304.45 to 3725.17) | -0.2 (-0.27 to -0.13) |
| Eastern Sub-Saharan Africa | 83535.9 (69059.76 to 99102.67) | 1113.57 (827.9 to 1448.15) | 179349.4 (149506.28 to 211544.14) | 1122.7 (839.98 to 1445.39) | 0.03 (0 to 0.05) |
| High-income Asia Pacific | 202871.04 (169577.95 to 236482.27) | 845.02 (637.6 to 1093.5) | 395393.99 (328061.79 to 467864.98) | 649.81 (484.67 to 847.02) | -0.88 (-1.04 to -0.72) |
| High-income North America | 975740.38 (813799.05 to 1147897.92) | 2104.71 (1567.7 to 2730.33) | 772480.86 (684682.46 to 870290.65) | 957.09 (779.28 to 1161.18) | -2.68 (-2.77 to -2.58) |
| North Africa and Middle East | 696131.88 (615708.34 to 779600.37) | 3860.3 (3202.82 to 4603.15) | 1563749.83 (1386925.03 to 1753015.63) | 3432.58 (2837.4 to 4095.12) | -0.41 (-0.43 to -0.4) |
| Oceania | 2962.68 (2404.62 to 3524.54) | 1068.48 (783.16 to 1395.17) | 7042.68 (5834.3 to 8312.06) | 1102.72 (816.54 to 1437.53) | 0.09 (-0.01 to 0.19) |
| South Asia | 1449672.73 (1236517.95 to 1695916.47) | 2510.27 (1945.83 to 3158.79) | 3980786.7 (3407074.8 to 4607186.39) | 2471.12 (1929.67 to 3087.96) | -0.06 (-0.1 to -0.02) |
| Southeast Asia | 201008.34 (169049.14 to 235496.86) | 772.98 (591.84 to 985.28) | 450127.6 (383694.58 to 521290.76) | 689.15 (534.81 to 863.4) | -0.45 (-0.71 to -0.18) |
| Southern Latin America | 78701.55 (70084.68 to 88087.55) | 1418.17 (1163.99 to 1696.28) | 97809.37 (84711.17 to 112658.82) | 923.09 (719.31 to 1152.34) | -1.5 (-1.6 to -1.39) |
| Southern Sub-Saharan Africa | 41852.62 (34477.47 to 49737.03) | 1394.39 (1028.72 to 1822.09) | 81244.47 (67641.37 to 95961.15) | 1324.33 (987.06 to 1719.44) | -0.17 (-0.2 to -0.14) |
| Tropical Latin America | 67505.4 (57304.52 to 78327.82) | 680.59 (524.09 to 857.52) | 170415.14 (147001.91 to 196440.68) | 588.12 (461.26 to 731.71) | -0.59 (-0.92 to -0.26) |
| Western Europe | 1422976.7 (1277672.87 to 1577988.48) | 1832.37 (1526.6 to 2172.14) | 1276076.25 (1114761.59 to 1444622.61) | 1069.17 (848.61 to 1324.15) | -1.85 (-1.88 to -1.81) |
| Western Sub-Saharan Africa | 107778.81 (89269.79 to 127595.17) | 1183.48 (878.92 to 1536.34) | 226867.13 (189565.28 to 266057.96) | 1241.92 (930.83 to 1600.46) | 0.16 (0.13 to 0.18) |

**Supplementary Table 3** Joinpoint regression analysis of ASRs for IHD in older adults globally from 1990 to 2019.

| Both |  |  |  |  |  |  |  |
| --- | --- | --- | --- | --- | --- | --- | --- |
| ASPR |  | ASIR |  | ASDR |  | ASMR |  |
| Year | APC_95%CI | Year | APC_95%CI | Year | APC_95%CI | Year | APC_95%CI |
| 1990-2000 | -0.18 (-0.2 to -0.17) | 1990-1995 | -0.42  (-0.49 to -0.34) | 1990-1994 | -0.49 (-0.9 to -0.07) | 1990-1994 | -0.65 (-1.04 to -0.26) |
| 2000-2004 | -0.43 (-0.5 to -0.36) | 1995-2000 | -1.17  (-1.27 to -1.06) | 1994-1998 | -1.97 (-2.6 to -1.34) | 1994-1998 | -2.07 (-2.67 to -1.47) |
| 2004-2009 | -0.34 (-0.38 to -0.29) | 2000-2005 | -0.35  (-0.46 to -0.25) | 1998-2003 | -0.85 (-1.25 to -0.45) | 1998-2003 | -0.95 (-1.34 to -0.56) |
| 2009-2014 | 0.01 (-0.04 to 0.05) | 2005-2009 | -1.45  (-1.61 to -1.29) | 2003-2006 | -2.02 (-3.32 to -0.7) | 2003-2006 | -1.89 (-3.15 to -0.62) |
| 2014-2017 | -0.22 (-0.36 to -0.08) | 1009-2017 | -0.85  (-0.89 to -0.81) | 2006-2015 | -1.51 (-1.67 to -1.35) | 2006-2017 | -1.42 (-1.53 to -1.31) |
| 2017-2019 | 0.05 (-0.1 to 0.19) | 2017-2019 | 0.12  (-0.24 to 0.49) | 2015-2019 | -0.89 (-1.48 to -0.3) | 2017-2019 | -0.36 (-2.24 to 1.56) |
| Female |  |  |  |  |  |  |  |
| ASPR |  | ASIR |  | ASDR |  | ASMR |  |
| Year | APC_95%CI | Year | APC_95%CI | Year | APC_95%CI | Year | APC_95%CI |
| 1990-1995 | -0.21 (-0.23 to -0.19) | 1990-1995 | -0.33 (-0.42 to -0.24) | 1990-1994 | -0.57 (-1 to -0.13) | 1990-1994 | -0.68 (-1.1 to -0.26) |
| 1995-2000 | -0.14 (-0.17 to -0.11) | 1995-2000 | -1.2 (-1.33 to -1.08) | 1994-1998 | -2.11 (-2.77 to -1.45) | 1994-1998 | -2.24 (-2.89 to -1.59) |
| 2000-2005 | -0.5 (-0.53 to -0.48) | 2000-2006 | -0.66 (-0.75 to -0.57) | 1998-2003 | -0.9 (-1.31 to -0.48) | 1998-2003 | -1.04 (-1.45 to -0.62) |
| 2005-2009 | -0.23 (-0.28 to -0.19) | 2006-2009 | -1.77 (-2.16 to -1.38) | 2003-2014 | -1.84 (-1.95 to -1.74) | 2003-2014 | -1.79 (-1.9 to -1.68) |
| 2009-2019 | 0.02 (0.02 to 0.03) | 2009-2017 | -0.75 (-0.8 to -0.69) | 2014-2019 | -0.86 (-1.26 to -0.46) | 2014-2019 | -0.99 (-1.39 to -0.58) |
|  |  | 2017-2019 | 0.05 (-0.39 to 0.49) |  |  |  |  |
| Male |  |  |  |  |  |  |  |
| ASPR |  | ASIR |  | ASDR |  | ASMR |  |
| Year | APC_95%CI | Year | APC_95%CI | Year | APC_95%CI | Year | APC_95%CI |
| 1990-1994 | -0.21 (-0.25 to -0.17) | 1990-1995 | -0.6 (-0.66 to -0.53) | 1990-1994 | -0.6 (-1 to -0.2) | 1990-1994 | -0.77 (-1.11 to -0.43) |
| 1994-2001 | -0.33 (-0.35 to -0.31) | 1995-2000 | -1.19 (-1.28 to -1.1) | 1994-1999 | -1.75 (-2.13 to -1.38) | 1994-1999 | -1.82 (-2.14 to -1.49) |
| 2001-2010 | -0.42 (-0.44 to -0.41) | 2000-2005 | -0.24 (-0.33 to -0.15) | 1999-2003 | -0.76 (-1.36 to -0.15) | 1999-2003 | -0.82 (-1.34 to -0.29) |
| 2010-2014 | 0 (-0.06 to 0.06) | 2005-2009 | -1.44 (-1.58 to -1.31) | 2003-2006 | -2.1 (-3.38 to -0.81) | 2003-2006 | -1.85 (-2.97 to -0.72) |
| 2014-2017 | -0.45 (-0.57 to -0.32) | 2009-2017 | -0.93 (-0.96 to -0.89) | 2006-2019 | -1.3 (-1.39 to -1.21) | 2006-2019 | -1.23 (-1.3 to -1.15) |
|  |  | 2017-2019 | 0.12 (-0.19 to 0.43) |  |  |  |  |

**Supplementary Table 4** Global and regional DALY of IHD among the elderly along with their trends from 1990 to 2019.

| DALY | 1990 |  | 2019 |  | 1990-2019 |
| --- | --- | --- | --- | --- | --- |
| Location | DALY Cases NO.(95%UI) | ASDR/100,000 (95% CI) | DALY Cases NO.(95%UI) | ASDR/100,000 (95% CI) | AAPC (95%CI) |
| Global | 74241584.82 (71320703.98 to 76755771.14) | 16358.05 (15498.72 to 17010.9) | 111169549.84 (104096499.62 to 117970464) | 11225.74 (10342.09 to 11960.64) | -1.29 (-1.48 to -1.1) |
| SDI |  |  |  |  |  |
| High SDI | 21639499.75 (20667679.22 to 22144146.8) | 15955.88 (15204.88 to 16405.87) | 14875063.64 (13832399.01 to 15613528) | 6014.48 (5567.25 to 6341.03) | -3.33 (-3.48 to -3.19) |
| High-middle SDI | 24920723.61 (23930719.66 to 25560790.46) | 19812.93 (18872.54 to 20467.63) | 32340952.34 (30008193.38 to 34345849.55) | 12689.83 (11628.36 to 13564.29) | -1.51 (-1.8 to -1.23) |
| Middle SDI | 14968063.6 (14115576.1 to 15895551.68) | 13816.33 (12881.2 to 14746.96) | 35997354.41 (33112066.55 to 38819543.54) | 12770.06 (11589.86 to 13829.07) | -0.3 (-0.42 to -0.17) |
| Low-middle SDI | 9131159.31 (8359776.56 to 9917539.8) | 14361.16 (12997.3 to 15760.18) | 20868904.7 (19010719.68 to 22821334.63) | 13483 (12107.31 to 14840.21) | -0.18 (-0.27 to -0.09) |
| Low SDI | 3541182.79 (3170901.4 to 3969462.92) | 14325.75 (12622.31 to 16179.29) | 7028038.98 (6261425.76 to 7899436.3) | 13051.37 (11438.54 to 14744.33) | -0.34 (-0.39 to -0.28) |
| GBD regions |  |  |  |  |  |
| Andean Latin America | 238968.2 (213249.83 to 267490.12) | 10634.61 (9189.13 to 12146.21) | 393648.63 (327006.82 to 468950.85) | 5994.4 (4831.4 to 7296.92) | -1.94 (-2.33 to -1.55) |
| Australasia | 551371.87 (525701.71 to 566349.28) | 18163.23 (17086.15 to 18935.63) | 314370.49 (287128.4 to 333524.3) | 4835.89 (4300.3 to 5270.47) | -4.45 (-4.79 to -4.11) |
| Caribbean | 573124.17 (544968.65 to 600665.92) | 18244.79 (17006.23 to 19378.95) | 763631.52 (670559.98 to 870733.46) | 12220.09 (10478.99 to 14027.11) | -1.25 (-1.44 to -1.07) |
| Central Asia | 1676966.08 (1611341.7 to 1720995.62) | 32104.86 (30484.81 to 33221.28) | 2671229.85 (2452828.76 to 2913748.32) | 36728.89 (33508.6 to 40063.49) | 0.56 (0.4 to 0.72) |
| Central Europe | 5265847.06 (5067142.72 to 5385190.39) | 28871.08 (27580.39 to 29727.87) | 4151581.53 (3661598.95 to 4657426.98) | 14282.6 (12366.21 to 16087.04) | -2.46 (-2.65 to -2.26) |
| Central Latin America | 1171755.74 (1115761.65 to 1212794.86) | 12851.58 (12111.65 to 13399.21) | 2543087.18 (2232391.35 to 2910639.29) | 9236.54 (7990.31 to 10609.61) | -1.27 (-1.46 to -1.09) |
| Central Sub-Saharan Africa | 328157.73 (272415.06 to 399139.17) | 14197.88 (11180.23 to 17993.28) | 625340 (479256.5 to 803786.18) | 12379.88 (9085.64 to 16402.44) | -0.47 (-0.63 to -0.32) |
| East Asia | 8569526.17 (7642622.09 to 9512372.88) | 9296.47 (8231.43 to 10451.49) | 24498135.36 (21375638.83 to 27722750.45) | 10137.46 (8744.46 to 11498.89) | 0.28 (0.06 to 0.5) |
| Eastern Europe | 10698699.78 (10368362.35 to 10897135.99) | 31096.72 (29816.84 to 31881.82) | 12390911.33 (11197695.8 to 13529248.58) | 27548.06 (24722.78 to 30212.13) | -0.44 (-0.99 to 0.11) |
| Eastern Sub-Saharan Africa | 809897.35 (701365.81 to 907598.84) | 10442.98 (8865.45 to 12004.53) | 1575877.75 (1283353.36 to 1867140.75) | 9765.44 (7812.18 to 11701.15) | -0.26 (-0.35 to -0.17) |
| High-income Asia Pacific | 1862481.13 (1766534.76 to 1924207.7) | 7739.03 (7247.78 to 8083.36) | 1617848.03 (1415758.19 to 1735507.62) | 2643.02 (2330.71 to 2864.08) | -3.65 (-3.93 to -3.36) |
| High-income North America | 8240901.32 (7833275.24 to 8457153.91) | 17826.72 (16925.88 to 18385.42) | 6467741.96 (6076884.28 to 6765536.71) | 8023.14 (7472.04 to 8440.44) | -2.74 (-2.9 to -2.57) |
| North Africa and Middle East | 5844697.59 (5431436.29 to 6280305.87) | 31771.65 (29085.86 to 34494.49) | 10019386.05 (8907610.1 to 11295945.92) | 21941.53 (19229.81 to 24833.42) | -1.25 (-1.49 to -1.01) |
| Oceania | 55866.66 (45658.76 to 70321.99) | 18101.25 (14320.32 to 23017.68) | 137491.1 (110544.89 to 170169.54) | 19751.91 (15704.2 to 24931.33) | 0.28 (0.21 to 0.36) |
| South Asia | 9509275.9 (8532222.59 to 10499410.87) | 16196.28 (14284.86 to 18127.27) | 23461531.83 (20714174.57 to 26333906.12) | 14613.99 (12739.98 to 16486.99) | -0.38 (-0.48 to -0.28) |
| Southeast Asia | 3108066.05 (2832769.05 to 3391843.32) | 11624.26 (10409.73 to 12871.69) | 7338246.61 (6655689.51 to 7998660.69) | 11072.81 (9860.13 to 12221.73) | -0.17 (-0.29 to -0.05) |
| Southern Latin America | 863802.73 (830862.79 to 888072.06) | 15326.74 (14331.35 to 16166.67) | 718688.95 (675501.96 to 756817.4) | 6809.26 (6158.34 to 7384.26) | -2.69 (-2.9 to -2.48) |
| Southern Sub-Saharan Africa | 264755.1 (236407.58 to 289251.1) | 8702.84 (7630.68 to 9715.24) | 572975.05 (522923.8 to 622609.67) | 9236.43 (8239.46 to 10225.48) | 0.24 (-0.2 to 0.68) |
| Tropical Latin America | 1536200.84 (1467178.81 to 1588370.69) | 15219.16 (14328.24 to 15912.28) | 2241413.95 (2089480.48 to 2353518.23) | 7752.15 (7114.5 to 8267.54) | -2.3 (-2.38 to -2.21) |
| Western Europe | 11865356.64 (11345032.89 to 12158100.87) | 15343.83 (14630.39 to 15807.6) | 6482622.46 (5989148.88 to 6824087.17) | 5257.78 (4852.03 to 5577) | -3.66 (-3.83 to -3.49) |
| Western Sub-Saharan Africa | 1205866.72 (1010297.45 to 1484402.79) | 13058.77 (10837.56 to 16119.84) | 2183790.22 (1842045.23 to 2535888.87) | 11835.89 (9852.8 to 13795.06) | -0.34 (-0.44 to -0.24) |

**Supplementary Table 5** Prevalence of IHD among older adults in 1990 and 2019 and the EAPC in different countries and territories

| Prevalence | 1990 |  | 2019 |  | 1990-2019 |
| --- | --- | --- | --- | --- | --- |
| Location | Prevalenct Cases NO.(95%UI) | ASPR/100,000 (95% CI) | Prevalenct Cases NO.(95%UI) | ASPR/100,000 (95% CI) | EAPC (95%CI) |
| Republic of Korea | 192368 (172466.27 to 213538.3) | 6435.18 (5585.75 to 7295.28) | 603495.27 (544306.25 to 670343.26) | 5398.69 (4728.13 to 6158.8) | -0.93 (-1.07 to -0.79) |
| Japan | 1545336.81 (1351512.26 to 1753069.53) | 7309.19 (6194.03 to 8565.62) | 2942107.47 (2586402.18 to 3312927.32) | 6192.22 (5279.15 to 7199.59) | -0.69 (-0.78 to -0.59) |
| Argentina | 320982.37 (285963.32 to 353774.15) | 8055.83 (7004.52 to 9146.3) | 436714.28 (398488.8 to 479187.53) | 6344.44 (5613.81 to 7164.31) | -0.86 (-0.92 to -0.8) |
| Peru | 85958.17 (71888.41 to 102883.29) | 6616 (5342.31 to 8250.26) | 245969.8 (204147.45 to 291757.92) | 6510.22 (5253.94 to 8139.02) | -0.05 (-0.13 to 0.02) |
| Bolivia (Plurinational State of) | 24269.42 (20126.72 to 29107.5) | 7022.6 (5616.13 to 8831.26) | 72387.63 (60217.28 to 86804.56) | 7196.22 (5786.25 to 8993.12) | 0.12 (0.09 to 0.15) |
| Ecuador | 43188.04 (35860.69 to 52109.3) | 7204.52 (5774.84 to 9095.08) | 133355.15 (110281.84 to 160590.33) | 7450.51 (5968.49 to 9386.06) | 0.14 (0.11 to 0.17) |
| Brunei Darussalam | 766.43 (684.06 to 856.7) | 8518.29 (7476.93 to 9668.11) | 2114.91 (1889.44 to 2367.45) | 7518.41 (6593.48 to 8568) | -0.49 (-0.55 to -0.43) |
| Portugal | 156209.96 (133672.35 to 181236.63) | 8448.62 (7043.32 to 10080.57) | 240698.43 (207087.6 to 279498.81) | 7677.87 (6367.88 to 9229.97) | -0.43 (-0.6 to -0.26) |
| Singapore | 22762.87 (20577.48 to 25155.82) | 9611.22 (8480.56 to 10843.16) | 71864.91 (67687.16 to 76283.06) | 7712.61 (7155.58 to 8326.4) | -0.69 (-0.76 to -0.62) |
| Chile | 107391.13 (96574.17 to 119768.48) | 9221.46 (8055.45 to 10475.71) | 234277.88 (212006.89 to 257960.11) | 7854.59 (6915.14 to 8856.92) | -0.52 (-0.63 to -0.4) |
| Uruguay | 47647.08 (42793.84 to 52972.09) | 9334.46 (8153.63 to 10666.27) | 55960.31 (50692.44 to 61871.13) | 7893.6 (6937.37 to 8958.82) | -0.69 (-0.75 to -0.63) |
| Indonesia | 802651.48 (662329.36 to 967309.41) | 8394.26 (6718.97 to 10522.5) | 1869884.92 (1532140.22 to 2282228.93) | 8496.36 (6748.23 to 10709.39) | -0.04 (-0.09 to 0.01) |
| Luxembourg | 7125.67 (6206.76 to 8111.53) | 9943.51 (8470.73 to 11662.29) | 10739.95 (9494.04 to 12132.84) | 8726.32 (7492.27 to 10157.45) | -0.42 (-0.52 to -0.31) |
| Thailand | 329804.92 (286493.91 to 377642.16) | 9138.07 (7754.51 to 10810.78) | 1091243.11 (948782.64 to 1262445.14) | 8915.99 (7526.17 to 10657.33) | -0.12 (-0.16 to -0.09) |
| Myanmar | 241417.63 (211070.99 to 277748.05) | 9929.55 (8462.36 to 11761.56) | 444577.37 (389023.87 to 507038.97) | 8975.11 (7653.21 to 10604.21) | -0.29 (-0.39 to -0.19) |
| Cyprus | 10020.72 (8687.76 to 11547.89) | 9387.98 (7921.65 to 11113.47) | 23073.52 (20015.48 to 26561.24) | 9020.55 (7622.65 to 10687.18) | -0.21 (-0.34 to -0.08) |
| United States of America | 6474855.47 (5645313.76 to 7342400.79) | 15313.02 (12962.48 to 17869.14) | 6570971.25 (5861058.36 to 7388417.83) | 9159.56 (7868.11 to 10564.92) | -2.1 (-2.26 to -1.94) |
| Cambodia | 43707.43 (37646.26 to 50728.14) | 9691.67 (8125.21 to 11662.43) | 115013.7 (99603.81 to 133600.17) | 9166.43 (7711.69 to 10983.11) | -0.2 (-0.23 to -0.16) |
| Lao People's Democratic Republic | 21136.3 (18459.85 to 24536.52) | 9775.92 (8307.12 to 11629.03) | 40342.37 (35247.27 to 46358.95) | 9281.4 (7900.67 to 11051.67) | -0.15 (-0.21 to -0.09) |
| Malta | 5877.41 (5147.31 to 6664.12) | 10864.72 (9253.02 to 12752.08) | 11734.17 (10436.54 to 13109.37) | 9394.98 (8124.45 to 10842.95) | -0.54 (-0.73 to -0.36) |
| France | 1285783.94 (1139386.46 to 1433419.79) | 11639.36 (10034.07 to 13368.24) | 1634318.62 (1456981.49 to 1820791.43) | 9402.72 (8171.81 to 10766.22) | -0.82 (-0.9 to -0.75) |
| Rwanda | 29428.87 (25498.19 to 33974.13) | 9635.04 (8059.69 to 11588.96) | 57548.49 (49906.48 to 66492.27) | 9410.28 (7888.79 to 11326.71) | -0.17 (-0.22 to -0.13) |
| Greece | 216062.89 (189907.15 to 244045.29) | 10834.52 (9253.14 to 12556.65) | 291309.11 (259531.28 to 327524.08) | 9446.47 (8191.86 to 10874.95) | -0.59 (-0.67 to -0.5) |
| Switzerland | 150705.8 (131766.64 to 170334.01) | 11033.21 (9427.73 to 12767.41) | 206014.88 (184187.16 to 227356.31) | 9449.81 (8271.79 to 10736.46) | -0.53 (-0.63 to -0.43) |
| Viet Nam | 437121.55 (377282.53 to 503541.75) | 9421.35 (7924.34 to 11275.86) | 935752.38 (810923.29 to 1079548.38) | 9463.83 (7970.69 to 11280.75) | 0.09 (0.06 to 0.13) |
| United Kingdom | 1586097.5 (1373950.34 to 1823102.15) | 12947.4 (10869.46 to 15290.9) | 1527104.13 (1338517.68 to 1734962.72) | 9464.19 (8048.91 to 11055.28) | -1.17 (-1.34 to -1) |
| Canada | 552242.94 (486497.53 to 611684.79) | 13243.57 (11440.4 to 14925.41) | 843439.62 (768541.5 to 920598.86) | 9498.89 (8479.07 to 10601.35) | -1.25 (-1.36 to -1.13) |
| Israel | 76151.55 (64760.47 to 87538.17) | 11847.43 (9821.94 to 13929.37) | 141649.26 (124995.26 to 158929.41) | 9587.28 (8269.21 to 11041.02) | -0.93 (-1.02 to -0.84) |
| Spain | 829471.71 (729614.15 to 936110.31) | 11451.29 (9817.3 to 13246.24) | 1160794.93 (1039021.93 to 1297741.04) | 9709.08 (8443.31 to 11178.47) | -0.73 (-0.82 to -0.65) |
| Guatemala | 32599.33 (28713.12 to 37114.54) | 8740.29 (7445.58 to 10292.8) | 127941.67 (111802.73 to 145740.43) | 9771.52 (8316.19 to 11561.92) | 0.48 (0.43 to 0.53) |
| Norway | 114932.68 (99312.88 to 131987.15) | 12388.6 (10386.55 to 14653.12) | 120077.22 (104951.06 to 136875.06) | 9843.79 (8335.36 to 11541.31) | -0.83 (-0.93 to -0.73) |
| Uganda | 68678.31 (59510.03 to 79618.79) | 9923.06 (8298.91 to 11985.25) | 138089.96 (120137.64 to 159437.36) | 9924.66 (8318.4 to 11967.31) | -0.02 (-0.07 to 0.03) |
| Democratic Republic of the Congo | 165345.68 (147119.56 to 186107.05) | 10773.83 (9296.26 to 12534.49) | 335497.59 (298747.94 to 376307.26) | 9933.44 (8588.45 to 11514.62) | -0.33 (-0.37 to -0.29) |
| Greenland | 376.78 (338.96 to 416.27) | 11697.9 (10284.71 to 13164.52) | 760.5 (691.25 to 839.12) | 9967.02 (8849.51 to 11207.55) | -0.61 (-0.65 to -0.57) |
| Timor-Leste | 2295.78 (1987.34 to 2654.91) | 9851.38 (8296.18 to 11799.56) | 9419.83 (8147.12 to 10876.83) | 9989.6 (8416.27 to 11921.02) | 0.06 (0.04 to 0.07) |
| Monaco | 1145.01 (1008.85 to 1287.04) | 11795.95 (10165.18 to 13567.36) | 1236.16 (1102.75 to 1377.56) | 9991.32 (8720.23 to 11369.15) | -0.72 (-0.79 to -0.64) |
| Andorra | 784.49 (694.21 to 892.82) | 11511.88 (9908.63 to 13352.35) | 1665.23 (1480.03 to 1857.92) | 9993.95 (8681.57 to 11444.46) | -0.76 (-0.86 to -0.67) |
| San Marino | 521.71 (458.07 to 591.73) | 11907.45 (10247.26 to 13793.72) | 806.03 (719.52 to 899.23) | 10022.89 (8740.3 to 11467.66) | -0.71 (-0.79 to -0.64) |
| Sweden | 241712.42 (206304.72 to 278608.42) | 11705.81 (9726.75 to 14008.56) | 275202.35 (236274.11 to 318204.25) | 10063.06 (8308.27 to 12120.46) | -0.6 (-0.65 to -0.56) |
| Brazil | 983282.08 (809407.45 to 1183366.9) | 10084.84 (8059.24 to 12679.37) | 2824701.31 (2353311.71 to 3369813.19) | 10073.93 (8129.3 to 12534.65) | 0.06 (0 to 0.12) |
| Eritrea | 8193.09 (7065.79 to 9483.88) | 9926.75 (8296.45 to 11945.58) | 24100.74 (21000.83 to 27780.28) | 10086.48 (8459.9 to 12089.01) | 0 (-0.03 to 0.04) |
| Denmark | 157852.5 (142952.87 to 174370.41) | 14638.18 (13002.05 to 16591.7) | 152609.75 (136163.47 to 169912.71) | 10130.39 (8831.51 to 11553.51) | -1.24 (-1.32 to -1.16) |
| Paraguay | 24654.89 (20512.53 to 29530.4) | 9835.81 (7906.35 to 12405.38) | 64335.44 (53544.64 to 77666.01) | 10214.2 (8237.05 to 12826.94) | 0.13 (0.09 to 0.16) |
| Central African Republic | 11193.37 (9901.78 to 12680.12) | 10429.49 (8928 to 12177.98) | 18948.44 (16799.39 to 21391.58) | 10253.09 (8809.62 to 11971.6) | -0.04 (-0.06 to -0.02) |
| Austria | 187197.03 (166240.14 to 210357.15) | 11737.58 (10167.49 to 13577.89) | 231830.94 (209772.1 to 254007.05) | 10285.52 (9133.77 to 11566.64) | -0.54 (-0.58 to -0.49) |
| Seychelles | 746.64 (645.79 to 865.12) | 10548.37 (8898.97 to 12561.76) | 1247.03 (1079.74 to 1437.62) | 10391.83 (8809.91 to 12340.02) | -0.07 (-0.11 to -0.04) |
| Lesotho | 10219.56 (9012.69 to 11647.01) | 9320.92 (7930.61 to 10991.96) | 13992.33 (12420.1 to 15842.52) | 10469.11 (8972.08 to 12264.05) | 0.41 (0.35 to 0.47) |
| El Salvador | 32039.14 (28020.34 to 36646.29) | 9582.68 (8159.84 to 11318.9) | 76094.01 (66848.18 to 86882.74) | 10484.12 (8963.15 to 12372.03) | 0.37 (0.32 to 0.41) |
| Sri Lanka | 131947.63 (114653.19 to 151050.73) | 11504.67 (9780.08 to 13632.38) | 323442.01 (280730.54 to 372451.75) | 10516.69 (8904.16 to 12495.66) | -0.31 (-0.36 to -0.26) |
| Maldives | 975.76 (844.64 to 1127.24) | 11821.09 (10018.43 to 13996.95) | 3110.49 (2706.75 to 3563.92) | 10535.8 (8915.6 to 12478.84) | -0.48 (-0.52 to -0.44) |
| Mauritius | 9285.42 (8054.51 to 10696.25) | 11358.8 (9607.77 to 13493.07) | 21942.55 (19004.85 to 25362.66) | 10560.99 (8899.47 to 12572.39) | -0.38 (-0.47 to -0.28) |
| Ethiopia | 190637.7 (162437.96 to 224108.22) | 9541.26 (7791.86 to 11696.15) | 456395.83 (389708.72 to 534910.03) | 10607.4 (8684.51 to 13028.08) | 0.35 (0.29 to 0.41) |
| Somalia | 23288.87 (20217.16 to 26873.2) | 10645.09 (8870.47 to 12835.04) | 68427.66 (58850.5 to 79302.01) | 10681.91 (8856.02 to 12910.67) | 0.01 (-0.02 to 0.03) |
| Ireland | 77406.48 (68489.68 to 87008.06) | 14182.72 (12242.75 to 16323.05) | 101840.4 (90747.36 to 113406.34) | 10686.01 (9269.4 to 12180.9) | -1.2 (-1.28 to -1.12) |
| Equatorial Guinea | 2104.73 (1879.88 to 2370.78) | 10677.69 (9185.86 to 12390.92) | 4972.73 (4457.42 to 5547.09) | 10728.47 (9310.11 to 12358.19) | 0.08 (0.03 to 0.13) |
| Mali | 43421.19 (38408.51 to 49378.59) | 9924.36 (8486.13 to 11632.07) | 94646.52 (84122.75 to 107379.73) | 10739.35 (9165.84 to 12600.45) | 0.34 (0.29 to 0.39) |
| Colombia | 224648.07 (198218.21 to 253519.26) | 12041.58 (10338.87 to 14076.85) | 685420.75 (601046.42 to 780356.29) | 10777.84 (9208.11 to 12684.33) | -0.4 (-0.44 to -0.37) |
| Zambia | 31140.4 (27208.59 to 35745.05) | 11015.3 (9301.85 to 13131.15) | 69172.65 (60666.94 to 78719.43) | 10803.91 (9159.52 to 12849.87) | -0.15 (-0.19 to -0.11) |
| Cameroon | 43202.55 (38228.16 to 48966.94) | 9641.11 (8186.41 to 11343.82) | 126619.14 (111156.52 to 143426.44) | 10812.21 (9183.65 to 12754.02) | 0.47 (0.39 to 0.56) |
| Angola | 38807.78 (34385.12 to 43652.56) | 11078.88 (9556.33 to 12852.06) | 108260.7 (96489 to 121650.64) | 10812.8 (9359.18 to 12507.46) | -0.08 (-0.1 to -0.07) |
| Belgium | 250949.87 (219540.15 to 282885.46) | 12259.24 (10423.29 to 14151.34) | 313621.36 (284958.14 to 346524.19) | 10831.74 (9599.12 to 12203.48) | -0.44 (-0.5 to -0.39) |
| Democratic People's Republic of Korea | 160755.21 (136833.2 to 189650.05) | 10234.11 (8477.5 to 12438.1) | 409726.65 (349356.9 to 482519.77) | 10860.83 (9018.8 to 13161.59) | 0.27 (0.23 to 0.32) |
| Finland | 145801.68 (126690.56 to 165331.17) | 15551.91 (13116.56 to 18063.57) | 178433.81 (160847.35 to 198309.06) | 10948.87 (9621.41 to 12500.26) | -1.31 (-1.41 to -1.22) |
| Niger | 28607.62 (25202.75 to 32694.6) | 10490.43 (8909.9 to 12432.48) | 84693.66 (74018.68 to 96877.61) | 10987.37 (9295.61 to 13048.33) | 0.16 (0.13 to 0.18) |
| Burkina Faso | 50105.49 (43478.38 to 57768.45) | 10610.62 (8901.64 to 12721.52) | 101868.91 (89101.02 to 117757.87) | 11144.98 (9375.18 to 13335.02) | 0.13 (0 to 0.25) |
| Guinea | 36346.41 (32194.73 to 41375.81) | 9582.73 (8138.2 to 11361.68) | 66089.26 (58039.95 to 75565.99) | 11205.7 (9498.48 to 13301.91) | 0.64 (0.59 to 0.69) |
| Iceland | 4641.28 (4038.43 to 5268.87) | 12663.91 (10712.02 to 14735.49) | 7828.67 (7067.96 to 8606.03) | 11253.25 (9969.49 to 12643.43) | -0.55 (-0.62 to -0.48) |
| Gabon | 6765.85 (6025.14 to 7611.36) | 10912.99 (9471.25 to 12628.23) | 11892.19 (10686.51 to 13339) | 11255.58 (9772.51 to 12985.56) | 0.12 (0.11 to 0.14) |
| Benin | 24519.18 (21712.45 to 27800.66) | 10936.86 (9313.2 to 12901) | 56600.34 (49797.83 to 64631.53) | 11461.4 (9746.61 to 13526.79) | 0.18 (0.16 to 0.2) |
| Panama | 18486.44 (16241.06 to 21017.03) | 10840.81 (9298.8 to 12795.27) | 56660.05 (49902.22 to 64630.8) | 11468.64 (9837.81 to 13474.02) | 0.23 (0.2 to 0.26) |
| Germany | 2408336.01 (2015096.55 to 2884280.8) | 14474.22 (11725.34 to 17613.72) | 2856575.78 (2585952.28 to 3140508.51) | 11488.71 (10112.19 to 12951.6) | -1.16 (-1.3 to -1.03) |
| Taiwan (Province of China) | 223353.47 (191192.21 to 261203.23) | 12626.32 (10605.14 to 15133.01) | 571774.01 (509416.01 to 639662.86) | 11532.05 (10037.11 to 13303.61) | -0.2 (-0.34 to -0.07) |
| Namibia | 10168.76 (8953.12 to 11569.85) | 12132.69 (10362.03 to 14272.69) | 17597.16 (15473.52 to 19953.57) | 11566.11 (9892.95 to 13547.94) | -0.19 (-0.25 to -0.13) |
| Nigeria | 478895.02 (415719.81 to 553250.04) | 10106.85 (8425.04 to 12111.09) | 970887.51 (840106.58 to 1126648.17) | 11592.5 (9629.23 to 13988.52) | 0.55 (0.46 to 0.63) |
| Costa Rica | 23597.15 (20769.55 to 26910.8) | 11765.47 (10064.27 to 13804.59) | 70614.37 (61887.02 to 80242.18) | 11653.24 (9949.4 to 13695.82) | -0.03 (-0.07 to 0) |
| Congo | 12674.27 (11197.78 to 14264.68) | 11675.82 (10062.55 to 13549.52) | 28299.44 (25256.94 to 31761.15) | 11675.99 (10068.71 to 13534.4) | 0.02 (-0.01 to 0.05) |
| Nicaragua | 18063.93 (15789.4 to 20694.29) | 11164.38 (9524.78 to 13199.42) | 56365.66 (49449.89 to 64675.33) | 11701.36 (9985.75 to 13823.66) | 0.18 (0.12 to 0.24) |
| United Republic of Tanzania | 121530.03 (106507.37 to 139440.77) | 10350.4 (8748.02 to 12295) | 299554.53 (263015.65 to 340839.7) | 11866.1 (10046.22 to 14103.04) | 0.51 (0.49 to 0.54) |
| Honduras | 24896.73 (21807.1 to 28536.89) | 11170.71 (9495.05 to 13249.18) | 81140.82 (70899.31 to 91868.25) | 12020 (10246.75 to 14163.81) | 0.29 (0.25 to 0.33) |
| Togo | 14409.46 (12682.11 to 16351.27) | 11748.14 (9994.64 to 13877.44) | 42002.85 (36954.94 to 48145.04) | 12061.14 (10216.17 to 14289.78) | 0.09 (0.04 to 0.14) |
| Italy | 1584589.58 (1380278.85 to 1817525.59) | 13368.01 (11253.75 to 15776.42) | 2203159.58 (1933842.9 to 2507200.46) | 12077.15 (10260.8 to 14099.01) | -0.54 (-0.61 to -0.46) |
| Ghana | 69700.43 (61714.48 to 78450.64) | 11187.96 (9577.34 to 13078.35) | 197224.3 (174232.88 to 224960.04) | 12079.74 (10318.58 to 14203.86) | 0.28 (0.21 to 0.35) |
| Mexico | 602698.53 (521661.95 to 698882.87) | 12812.69 (10725.33 to 15375.9) | 1635782.6 (1412464.35 to 1900349.51) | 12103.91 (10146.28 to 14538.03) | -0.29 (-0.34 to -0.24) |
| Botswana | 6867.6 (6042 to 7828.61) | 11355.85 (9688.2 to 13351.33) | 16253.48 (14399.37 to 18332.04) | 12121.78 (10437.28 to 14150.01) | 0.23 (0.21 to 0.25) |
| Madagascar | 62733.9 (54515.53 to 72852.04) | 11559.05 (9671.93 to 13889.72) | 119941.66 (104057.31 to 139049.96) | 12131.1 (10173.38 to 14589.58) | 0.17 (0.14 to 0.21) |
| Eswatini | 3430.23 (3013.2 to 3915.47) | 11764.22 (10011.73 to 13932.91) | 7332.8 (6437.03 to 8344.34) | 12192.45 (10410.39 to 14321.13) | 0.13 (0.06 to 0.21) |
| Burundi | 30179.73 (26178.31 to 34995.43) | 11765.76 (9836.3 to 14191.23) | 53704.33 (46508.25 to 62063.16) | 12211.16 (10160.79 to 14728.84) | 0.1 (0.07 to 0.13) |
| Guinea-Bissau | 4890.98 (4280.98 to 5633.06) | 11664.35 (9902.44 to 13823.63) | 8433.67 (7393.41 to 9636.5) | 12271.98 (10371.55 to 14547.8) | 0.22 (0.18 to 0.26) |
| Zimbabwe | 50053.82 (43880.63 to 56970.74) | 11628.18 (9905.38 to 13725.19) | 87130.93 (77133.89 to 99206.93) | 12272.4 (10492.05 to 14431.73) | 0.13 (0.01 to 0.26) |
| Micronesia (Federated States of) | 644.46 (552.15 to 760.31) | 12924.67 (10790.16 to 15632.95) | 819.92 (700.18 to 973.54) | 12344.59 (10287.61 to 15000.25) | -0.2 (-0.21 to -0.19) |
| Venezuela (Bolivarian Republic of) | 141731.73 (125567.85 to 160795.34) | 13490.82 (11627.89 to 15748.08) | 413678.51 (364727.44 to 469342.18) | 12385.71 (10630.36 to 14540.86) | -0.34 (-0.38 to -0.3) |
| Chad | 34437.99 (30099.47 to 39636.67) | 10610.73 (8969.9 to 12658.44) | 70500.1 (61274.05 to 81040.47) | 12427.62 (10407.85 to 14916.07) | 0.59 (0.55 to 0.63) |
| Cabo Verde | 3464.92 (3065.36 to 3936.67) | 11444.83 (9758.07 to 13433.94) | 5843.63 (5182.22 to 6614.4) | 12475.97 (10642.28 to 14627.88) | 0.3 (0.25 to 0.35) |
| Comoros | 3273.83 (2816.8 to 3808.48) | 13012.29 (10865.89 to 15665.7) | 6766.65 (5875.18 to 7765.66) | 12504.42 (10475.1 to 15005.93) | -0.18 (-0.22 to -0.14) |
| Malaysia | 118467.67 (103149.89 to 135601.62) | 12447.15 (10574.87 to 14659.67) | 376062.12 (327959.82 to 434325.24) | 12712.63 (10858.8 to 15014.32) | 0.14 (0.1 to 0.18) |
| Sao Tome and Principe | 872.99 (766.93 to 996.82) | 11518.72 (9804.32 to 13586.06) | 1339.35 (1177.37 to 1524.19) | 12728.66 (10780.63 to 15042.08) | 0.36 (0.32 to 0.41) |
| Mozambique | 75478.36 (64937.13 to 87448.48) | 12229.74 (10185.78 to 14781.51) | 140376.16 (121580 to 162648.04) | 12773.25 (10705.28 to 15376.3) | 0.21 (0.18 to 0.25) |
| South Africa | 314419.52 (274019 to 361525.03) | 13651.03 (11479.43 to 16193.18) | 634986.38 (553842 to 730273.22) | 12777.83 (10754.73 to 15125.39) | -0.36 (-0.48 to -0.25) |
| China | 10948061.65 (9183514.53 to 12992266.75) | 11959.01 (9805.64 to 14645.87) | 30908973.93 (26130146.51 to 36253066.48) | 12801.53 (10555.78 to 15515.42) | 0.29 (0.24 to 0.34) |
| Poland | 972917.12 (818585.34 to 1155453.22) | 17695.25 (14285.28 to 21905.89) | 1195983.64 (989334.83 to 1428554.22) | 12850.45 (10297.27 to 16078.1) | -1.28 (-1.37 to -1.19) |
| Malawi | 51970.56 (44713.03 to 60468.79) | 12896.27 (10684.21 to 15601.37) | 98022.16 (84146.89 to 114096.31) | 12877.28 (10622.03 to 15616.08) | -0.06 (-0.15 to 0.02) |
| Netherlands | 393491.64 (341938.63 to 445074.88) | 15155.23 (12786.99 to 17548.03) | 573372.21 (515879.3 to 634318.99) | 12944.19 (11381.62 to 14654.87) | -0.76 (-0.83 to -0.7) |
| Kenya | 114407.42 (96693.41 to 135003.6) | 13117.91 (10661.9 to 16189.56) | 284320.69 (240381.65 to 336900.99) | 13044.13 (10618.95 to 16054.88) | -0.08 (-0.17 to 0.01) |
| Mauritania | 13500.75 (11917.08 to 15275.61) | 12029.51 (10291.33 to 14065.46) | 29896.94 (26412.9 to 33985.8) | 13126.24 (11195.22 to 15476.66) | 0.3 (0.25 to 0.35) |
| Liberia | 16193.15 (14247.96 to 18474.39) | 12457.05 (10591.23 to 14694.49) | 25715.79 (22676.78 to 29588.4) | 13141.28 (11147.74 to 15640.87) | 0.31 (0.26 to 0.36) |
| Senegal | 45560.32 (40091.08 to 51773.01) | 13055.88 (11083.2 to 15394.46) | 105033.5 (92252.2 to 119948.95) | 13180.49 (11171.14 to 15644.14) | 0.03 (-0.04 to 0.1) |
| Marshall Islands | 211.83 (181.33 to 247.43) | 12402.55 (10354.65 to 14935.86) | 411.86 (351.81 to 490.01) | 13235.28 (11069.46 to 16031.54) | 0.2 (0.17 to 0.24) |
| South Sudan | 30929.7 (26999.01 to 35577.78) | 11996.43 (10112.39 to 14311.6) | 48332.3 (41969.84 to 55561.62) | 13343.38 (11217.82 to 15927.79) | 0.36 (0.29 to 0.44) |
| Solomon Islands | 1655.62 (1409.97 to 1961.19) | 13513.47 (11278.58 to 16318.87) | 3579.84 (3067.22 to 4222.49) | 13405 (11167.82 to 16213.87) | -0.1 (-0.12 to -0.07) |
| Djibouti | 1463.28 (1268.18 to 1697.3) | 12220.7 (10282.27 to 14631.69) | 7372.59 (6355.35 to 8571.13) | 13469.24 (11269.21 to 16176.42) | 0.33 (0.3 to 0.37) |
| Philippines | 407397.55 (354390.62 to 467920.65) | 13284.84 (11277.67 to 15709.52) | 1096947.89 (948709.75 to 1262383.43) | 13481.96 (11399.66 to 16046.11) | -0.09 (-0.23 to 0.06) |
| Sierra Leone | 29406.09 (25807.12 to 33788.56) | 13343.21 (11329.65 to 15842.39) | 50244.08 (43952.13 to 57610.71) | 13482.78 (11408.4 to 16035.88) | 0.06 (0.03 to 0.1) |
| Tuvalu | 97.51 (83.02 to 115.01) | 12853.94 (10701.15 to 15569.63) | 159.28 (136.09 to 187.3) | 13614.77 (11368.75 to 16416.55) | 0.17 (0.12 to 0.21) |
| Kiribati | 477.67 (409.09 to 565.02) | 13283.06 (11044.78 to 16079.06) | 861.11 (733 to 1025.09) | 13730.67 (11378.48 to 16738.75) | 0.12 (0.09 to 0.15) |
| Gambia | 4636.53 (4073.57 to 5295.23) | 12740.05 (10798.93 to 15004.31) | 14200.79 (12457.56 to 16177.29) | 13765.67 (11675.05 to 16282.87) | 0.33 (0.28 to 0.38) |
| Nauru | 38.76 (33.21 to 45.33) | 13731.94 (11516.18 to 16474.12) | 37.43 (31.87 to 44.14) | 13786.48 (11524.23 to 16545.2) | -0.01 (-0.07 to 0.05) |
| New Zealand | 90847.11 (79813.18 to 103130.7) | 17764.79 (15200.76 to 20606.31) | 139484.24 (123068.71 to 157335.2) | 13810.78 (11838.08 to 15919.98) | -0.9 (-0.98 to -0.82) |
| Niue | 37.37 (32.17 to 43.65) | 13262.25 (11102.08 to 15966.4) | 36.79 (31.55 to 42.75) | 13814.2 (11541.72 to 16589.76) | 0.11 (0.06 to 0.15) |
| Northern Mariana Islands | 163.52 (141.34 to 189.89) | 13019.7 (11032.49 to 15461.31) | 700.84 (602.91 to 820.72) | 13847.48 (11621.17 to 16567.76) | 0.16 (0.1 to 0.22) |
| Palau | 147.87 (126.56 to 172.87) | 13583.73 (11362 to 16296.06) | 296.45 (253.15 to 348.59) | 13987.94 (11694.8 to 16781.7) | 0.06 (0.02 to 0.1) |
| Papua New Guinea | 23419.82 (20056.2 to 27450.35) | 13321.54 (11150.04 to 16046.87) | 58529.06 (50317.4 to 68587.65) | 14061.62 (11788.82 to 16908.69) | 0.19 (0.14 to 0.24) |
| Guam | 925.67 (799.59 to 1081.51) | 12384.11 (10476.06 to 14690.35) | 3148.34 (2710.2 to 3655.28) | 14246.18 (12011.81 to 16937.78) | 0.47 (0.39 to 0.55) |
| Samoa | 1376.62 (1182.6 to 1612.46) | 14215.23 (11899.98 to 17076.56) | 2223.01 (1911.28 to 2594.63) | 14270.93 (11938.58 to 17157.77) | -0.03 (-0.06 to 0.01) |
| Tokelau | 24.09 (20.75 to 28.22) | 13448.62 (11260.14 to 16233.67) | 22.06 (18.93 to 25.76) | 14281.9 (11958.7 to 17173.25) | 0.18 (0.16 to 0.2) |
| Cook Islands | 192.97 (166.37 to 224.25) | 13944.82 (11714.41 to 16641.5) | 442.69 (383.63 to 515.46) | 14358.85 (12130.96 to 17174.68) | 0.06 (0.02 to 0.1) |
| Tonga | 875.41 (754.95 to 1022.68) | 14419.46 (12100.53 to 17298.35) | 1348.37 (1164.33 to 1565.8) | 14625.54 (12224.1 to 17572.77) | 0 (-0.03 to 0.03) |
| American Samoa | 310.4 (265.79 to 362.85) | 14051.05 (11832.51 to 16814.86) | 762.57 (656.16 to 892.71) | 14751.87 (12398.48 to 17728.91) | 0.14 (0.1 to 0.18) |
| Fiji | 4856.13 (4179.43 to 5689.22) | 14693.13 (12279.84 to 17708.5) | 11323.83 (9713.41 to 13315.51) | 14758.44 (12343.29 to 17706.54) | 0.04 (0.03 to 0.06) |
| Vanuatu | 1041.28 (893.82 to 1229.34) | 15653.45 (13079.83 to 18923.65) | 2859.99 (2456.35 to 3360.9) | 15416.39 (12886.15 to 18586.68) | -0.07 (-0.09 to -0.06) |
| Albania | 37522.02 (32357.27 to 43444.37) | 16600.39 (13925.22 to 19890.14) | 87191.56 (76349.33 to 99466.46) | 15558.54 (13237.99 to 18356.61) | -0.19 (-0.23 to -0.15) |
| Slovakia | 169305.84 (147919.86 to 194183.33) | 22214.82 (18861.51 to 26261.95) | 200372.21 (175201.07 to 228664.85) | 16618.95 (14148.64 to 19546.19) | -1.09 (-1.15 to -1.04) |
| Australia | 490744.54 (450703.5 to 529472.35) | 19380.05 (17519.13 to 21261.13) | 917149.57 (848091.91 to 994213.51) | 17055.52 (15455.18 to 18750.58) | -0.44 (-0.47 to -0.41) |
| Cuba | 247839.91 (223248.65 to 276003.85) | 19193.6 (16920.45 to 21888.09) | 398519.36 (358654.46 to 441527.04) | 17075.23 (15027.88 to 19507.17) | -0.4 (-0.45 to -0.36) |
| North Macedonia | 40053.29 (34651.7 to 46151.51) | 19016.73 (15951.92 to 22702.34) | 70430.16 (61366.6 to 80617.18) | 17288.96 (14671.8 to 20464.87) | -0.36 (-0.38 to -0.35) |
| Romania | 692142.83 (604289.86 to 792460.48) | 20214.99 (17228.75 to 23881.17) | 877727.76 (765993.97 to 1005843.34) | 17330.04 (14712.09 to 20507.46) | -0.7 (-0.76 to -0.64) |
| Slovenia | 64238.76 (55394.34 to 74276.61) | 20957.13 (17631.68 to 25053.21) | 99047.2 (86756.53 to 113478.29) | 17440.39 (14786.43 to 20673.33) | -0.75 (-0.81 to -0.7) |
| Croatia | 160773.87 (138285.27 to 187103.87) | 20418.68 (17052.77 to 24536.97) | 208500.31 (179985.53 to 239722.78) | 17487.23 (14663.43 to 20951.46) | -0.56 (-0.61 to -0.5) |
| Haiti | 63479.14 (56637.48 to 71058.04) | 18433.55 (16110.31 to 21240.57) | 129493.05 (116151.11 to 144743.6) | 17949.67 (15635.49 to 20665.91) | -0.1 (-0.12 to -0.07) |
| Bermuda | 1437.8 (1292.55 to 1591.84) | 18995.18 (16725.57 to 21586.9) | 3031.98 (2741.47 to 3370.84) | 17951.1 (15855.06 to 20374.33) | -0.22 (-0.24 to -0.2) |
| Bulgaria | 319530.45 (278160.68 to 366851.26) | 20300.45 (17315.3 to 23843.23) | 368854.3 (322034.91 to 419443.86) | 18245.66 (15545.6 to 21418.56) | -0.6 (-0.69 to -0.51) |
| Bosnia and Herzegovina | 83402.67 (72341.27 to 96707.02) | 19231.4 (16152.75 to 22959.42) | 144683.69 (126603.12 to 165006.16) | 18429.81 (15680.25 to 21759.65) | -0.13 (-0.19 to -0.08) |
| United States Virgin Islands | 1740.26 (1560.91 to 1935.34) | 18692.02 (16423.1 to 21344.28) | 4747.1 (4271.35 to 5265.72) | 18918.46 (16672.97 to 21515.71) | 0.06 (0.03 to 0.08) |
| Nepal | 187083.38 (168279.42 to 208553.36) | 20081.39 (17576.31 to 22838.91) | 482747.69 (437091.56 to 535078.5) | 19112.07 (16883.21 to 21618.67) | -0.17 (-0.24 to -0.1) |
| Dominican Republic | 73993.96 (66429.41 to 82820.43) | 17828.41 (15628.65 to 20490.18) | 205509.33 (184626.8 to 227368.5) | 19131.89 (16855.24 to 21727.59) | 0.27 (0.23 to 0.3) |
| Antigua and Barbuda | 1371.92 (1233.35 to 1532.34) | 19543.57 (17196.45 to 22268.76) | 2239.48 (2018.47 to 2481.73) | 19173.76 (16908.72 to 21789.59) | -0.1 (-0.11 to -0.09) |
| Turkey | 989587.55 (900945.8 to 1086345.07) | 25928.74 (23207.19 to 29009.21) | 2016764.43 (1811673.45 to 2247781.37) | 19553.2 (17284.29 to 22164.18) | -1.07 (-1.16 to -0.99) |
| Dominica | 1867.29 (1684.95 to 2082.12) | 18866.23 (16536.99 to 21523.13) | 2211.44 (1987 to 2454.12) | 19553.61 (17168.86 to 22317.08) | 0.19 (0.16 to 0.22) |
| Hungary | 479379.11 (419480.67 to 547960.86) | 24918 (21264.65 to 29260.03) | 514940.34 (453546.98 to 581348.11) | 19647.95 (16860.21 to 23005.59) | -0.92 (-0.95 to -0.88) |
| Montenegro | 15595.72 (13465.21 to 18053.04) | 21417.18 (18025.34 to 25532.89) | 25483.5 (22187.61 to 29230.66) | 19769.26 (16764.24 to 23394.73) | -0.29 (-0.32 to -0.25) |
| Bahamas | 3361.19 (3006.06 to 3720.76) | 19783.39 (17379.87 to 22507.15) | 8474.74 (7628.08 to 9367.95) | 19853.76 (17463.79 to 22584.24) | 0.07 (0.04 to 0.09) |
| Saint Kitts and Nevis | 1027.46 (920.97 to 1143.03) | 19205.34 (16861.54 to 22025.87) | 1421.24 (1276.32 to 1587.31) | 19879.93 (17507.65 to 22656.99) | 0.14 (0.13 to 0.16) |
| Guyana | 8405.77 (7499.34 to 9463.19) | 20379.49 (17772.3 to 23524.66) | 13492.72 (12101.33 to 15100.92) | 20045.91 (17552.34 to 22945.23) | -0.04 (-0.08 to 0) |
| Saint Vincent and the Grenadines | 1792.59 (1604.46 to 2004.8) | 19397.21 (16926.16 to 22266.13) | 3314.22 (2979.64 to 3692.32) | 20145.08 (17729.67 to 23001.57) | 0.17 (0.14 to 0.2) |
| Czechia | 461329.69 (406402.23 to 522944.9) | 25518.07 (21997.8 to 29574.74) | 576020.74 (506244.17 to 652521.91) | 20357.97 (17507.71 to 23642.28) | -0.83 (-0.92 to -0.75) |
| Barbados | 8125.59 (7306.48 to 9060.97) | 20026.54 (17586.27 to 22751.84) | 12956.66 (11669.82 to 14329.94) | 20451.83 (18026.89 to 23276.72) | 0.06 (0.05 to 0.08) |
| Grenada | 1882.9 (1694.55 to 2107.01) | 19461.42 (17056.58 to 22366.97) | 2635.61 (2370.63 to 2924.69) | 20548.18 (18069.11 to 23362.1) | 0.17 (0.15 to 0.2) |
| Lithuania | 127814.14 (114306.41 to 143166.96) | 22465.31 (19496.1 to 26011.2) | 161531.8 (144130.75 to 180240.33) | 20670.24 (17946.21 to 23882.5) | -0.25 (-0.3 to -0.2) |
| Saint Lucia | 2297.14 (2066.49 to 2569.57) | 20551.66 (18024.41 to 23480.7) | 5287.05 (4753.41 to 5889.6) | 20726.12 (18178.49 to 23655.64) | 0.02 (0 to 0.05) |
| Puerto Rico | 96265.9 (86250.47 to 106872.98) | 20692.22 (18230.08 to 23568.5) | 196440.76 (177919.29 to 217824.75) | 20796.35 (18340.3 to 23617.56) | 0.02 (-0.02 to 0.06) |
| Jamaica | 43616.28 (39129.55 to 48613.99) | 18916.46 (16580.01 to 21626.37) | 74172.44 (66761.51 to 82475.1) | 21058.61 (18475.03 to 24039.99) | 0.43 (0.39 to 0.46) |
| Republic of Moldova | 110352.91 (98650.85 to 122055.77) | 21728.43 (18964.79 to 24805.12) | 156243.09 (139977.18 to 174309.58) | 21397.76 (18636.82 to 24659.08) | -0.08 (-0.11 to -0.06) |
| Bangladesh | 1020074.65 (921065.02 to 1130289.32) | 20501.25 (18018.84 to 23281.05) | 3271220.12 (2978540.41 to 3621897.55) | 21489.39 (19021.26 to 24318.12) | 0.19 (0.13 to 0.25) |
| Belize | 2221.18 (1993.23 to 2471.63) | 20224.15 (17693.52 to 23155.6) | 6277.17 (5640.3 to 6986.56) | 21659.95 (19065.93 to 24697.28) | 0.25 (0.21 to 0.3) |
| Suriname | 5711.67 (5121.58 to 6371.56) | 19918.28 (17435.59 to 22834.7) | 14816.27 (13328.47 to 16483.19) | 21693.97 (19059.47 to 24747.66) | 0.31 (0.27 to 0.36) |
| Bhutan | 4875.7 (4378.88 to 5400.64) | 20669.38 (18180.33 to 23498.2) | 13751.02 (12486.93 to 15135.8) | 21727.83 (19178.34 to 24517.66) | 0.17 (0.13 to 0.21) |
| India | 9067170.22 (7941496.25 to 10331699.39) | 20778.8 (17692.47 to 24256.65) | 28465144.1 (24943146.07 to 32400999.41) | 21985.76 (18815.81 to 25564.75) | 0.25 (0.19 to 0.3) |
| Russian Federation | 4733208.31 (4171014.98 to 5395125.58) | 21633.14 (18394.82 to 25366.46) | 6686813.69 (5893901.69 to 7604407.13) | 22099.24 (18789.85 to 25958.52) | -0.01 (-0.07 to 0.05) |
| Latvia | 100773.31 (89661.89 to 113291.19) | 22082.19 (19115.46 to 25640.52) | 121337.02 (107716.92 to 136418.54) | 22184.49 (19177.83 to 25790.98) | -0.04 (-0.06 to -0.01) |
| Serbia | 308506.23 (271412.36 to 352005.74) | 22949.31 (19655.14 to 26913.74) | 491780.08 (434318.12 to 556300.92) | 22200.27 (19133.8 to 25880.49) | -0.21 (-0.24 to -0.17) |
| Belarus | 347263.14 (311803.97 to 387822.65) | 21619.7 (18912.93 to 24748.67) | 455670.7 (411396.07 to 502687.22) | 22368.33 (19606.17 to 25426.07) | 0.1 (-0.02 to 0.22) |
| Kyrgyzstan | 81957.5 (74392.19 to 90502.58) | 23200.97 (20394.17 to 26358.83) | 104065.74 (94163.93 to 115099.09) | 22666.08 (19992.68 to 25699.28) | -0.08 (-0.1 to -0.06) |
| Kazakhstan | 354245.89 (320450.37 to 390208.76) | 25182.02 (22294.2 to 28430.35) | 439909.65 (397876.33 to 485867.94) | 23297.23 (20635.81 to 26297.2) | -0.44 (-0.58 to -0.29) |
| Trinidad and Tobago | 24623.27 (22178.72 to 27441.72) | 24040.87 (21109.95 to 27467.19) | 53978.04 (48476.99 to 59882.85) | 23610.98 (20788.98 to 26866.35) | -0.06 (-0.09 to -0.03) |
| Ukraine | 2138914.21 (1878803.5 to 2438789.23) | 23654.75 (20024.13 to 27826.52) | 2546315.12 (2245192.64 to 2891218.11) | 25206 (21382.02 to 29622.98) | 0.14 (0.02 to 0.26) |
| Armenia | 74917.21 (68047.31 to 82673.26) | 25782.97 (22788.49 to 29116.37) | 132273.86 (119852.36 to 146003.17) | 25274.84 (22352.44 to 28575.13) | -0.05 (-0.07 to -0.02) |
| Georgia | 197017.33 (179416.28 to 215334.37) | 26500.76 (23594.19 to 29759.89) | 197387.9 (177802.8 to 219600.9) | 25435.91 (22392.42 to 28941.27) | -0.18 (-0.2 to -0.16) |
| Pakistan | 1517693.9 (1330556.84 to 1725414.15) | 23924.34 (20353.93 to 27948.36) | 2820676.32 (2480518.49 to 3207567.03) | 25855.12 (22155.14 to 30072.14) | 0.28 (0.24 to 0.33) |
| Tajikistan | 77459.47 (70214.6 to 85609.83) | 24741.98 (21761.69 to 28054.38) | 111788.36 (101649.15 to 123494.62) | 26119.14 (23232.25 to 29307.59) | 0.22 (0.2 to 0.24) |
| Mongolia | 30125.82 (27178.99 to 33211.57) | 26156.64 (23074.36 to 29602.47) | 55233.82 (49990.87 to 60910.46) | 26396.07 (23354.64 to 29821.36) | 0.03 (-0.01 to 0.06) |
| Turkmenistan | 50835.95 (46160.2 to 55941.25) | 25154.17 (22277.99 to 28398.51) | 101802.15 (92957.91 to 112233.24) | 26470.76 (23578.96 to 29801.3) | 0.12 (0.09 to 0.15) |
| Estonia | 67316.63 (60462.74 to 74934.49) | 25801.73 (22573.68 to 29390.45) | 95919.99 (86436.86 to 106145.53) | 26575.92 (23344.78 to 30251.43) | 0.12 (0.11 to 0.13) |
| Azerbaijan | 137727.65 (124670.36 to 150800.28) | 26478.14 (23477.19 to 29803.59) | 244392.51 (223520.21 to 267756.39) | 26826.49 (23972.17 to 29938.88) | 0.01 (-0.01 to 0.03) |
| Tunisia | 153216.25 (140111.68 to 168011.11) | 27160.81 (24448.79 to 30172.75) | 405692.73 (372394.69 to 442114.92) | 27576.76 (24916.19 to 30520.72) | 0.03 (0.01 to 0.05) |
| Algeria | 398715.88 (365299.15 to 435042.33) | 29866.26 (27064.83 to 32845.2) | 1043896.09 (955298.51 to 1137069.53) | 27977.14 (25235.94 to 31015.82) | -0.26 (-0.29 to -0.24) |
| Uzbekistan | 278092.52 (251456.56 to 306518.09) | 22213.86 (19638.56 to 25117.61) | 498986.61 (458476.35 to 544072.65) | 29003.04 (26382.26 to 32026.84) | 0.95 (0.81 to 1.09) |
| Qatar | 2034.44 (1856.3 to 2235.66) | 28764.05 (25942.19 to 31903.44) | 18272.65 (16436.62 to 20108.07) | 29649.83 (26650.11 to 32826.14) | 0.05 (0.02 to 0.08) |
| Yemen | 142822.27 (130849.72 to 155649.3) | 29019.36 (26185.86 to 32062.87) | 398518.97 (364273.96 to 434311.48) | 29758.42 (26812.44 to 33066.72) | 0.09 (0.08 to 0.1) |
| Libya | 53924.1 (49490.98 to 58712.88) | 27878.02 (25166.3 to 30890.79) | 152577.92 (139250.58 to 166522.79) | 30235.8 (27166.55 to 33577.57) | 0.23 (0.18 to 0.28) |
| Palestine | 28195.83 (25780.99 to 30824.95) | 29393.25 (26398.16 to 32728.38) | 69579.12 (63756.28 to 75854.82) | 30238.32 (27231.92 to 33443.5) | 0.11 (0.09 to 0.13) |
| Lebanon | 74979.1 (68805.19 to 81431.85) | 29882.71 (26997.89 to 33036.89) | 196243.01 (179991.85 to 212804.09) | 30412.77 (27439.07 to 33718.68) | 0.15 (0.1 to 0.21) |
| Jordan | 39651.55 (36100.26 to 43585.25) | 31801.23 (28567.28 to 35302.61) | 197770.89 (180283.34 to 217254.07) | 31094.33 (27920.65 to 34679.64) | -0.1 (-0.14 to -0.07) |
| Sudan | 309878.42 (284444 to 336962.41) | 30281.03 (27346.76 to 33451.21) | 585288.61 (534745.02 to 635725.72) | 31153.7 (28093.76 to 34548.14) | 0.09 (0.07 to 0.1) |
| Afghanistan | 259267.68 (237894.95 to 283101.72) | 32663.92 (29455.08 to 36223.7) | 333198.03 (304533.58 to 364880.02) | 31378.84 (28240.13 to 35017.22) | -0.18 (-0.22 to -0.15) |
| Syrian Arab Republic | 160835.3 (148348.59 to 175307.39) | 30148.29 (27293.88 to 33342.37) | 412105.97 (378785.61 to 448978.66) | 31431.35 (28497.04 to 34700.7) | 0.07 (0.03 to 0.11) |
| Bahrain | 5132.66 (4666.1 to 5635.14) | 33120.09 (29780.35 to 36810.1) | 26172.01 (23715.18 to 28877.47) | 31665.78 (28509 to 35195.78) | -0.19 (-0.23 to -0.14) |
| United Arab Emirates | 7527.7 (6852.52 to 8248.63) | 29178.1 (26275.58 to 32339.96) | 61170.94 (55315.98 to 67605.29) | 31723.61 (28676.47 to 35162.8) | 0.33 (0.3 to 0.36) |
| Morocco | 484029.71 (442222.92 to 529332.07) | 32650.4 (29337.31 to 36248.87) | 1071143.03 (979382.64 to 1174105.69) | 31880.05 (28681.59 to 35488.95) | -0.16 (-0.21 to -0.1) |
| Saudi Arabia | 159975.85 (146324.38 to 174375) | 28466.66 (25641.41 to 31473.86) | 432804.3 (395723.49 to 474133.97) | 31930.05 (28929.09 to 35248.98) | 0.46 (0.4 to 0.52) |
| Iraq | 286466.14 (262524.74 to 312209.8) | 34180.55 (30926.26 to 37840.6) | 748230.78 (684561.37 to 819068.89) | 32702.69 (29522.81 to 36143.27) | -0.15 (-0.17 to -0.13) |
| Oman | 18388.12 (16804.12 to 20160.05) | 30319.47 (27249.46 to 33704.88) | 46558.64 (42480.46 to 51170.48) | 33641.85 (30415.55 to 37268.54) | 0.44 (0.39 to 0.49) |
| Kuwait | 16860.66 (15393.67 to 18411.59) | 32896.92 (29619.2 to 36519.78) | 73896.18 (67514.59 to 80560.78) | 34247.57 (30936.65 to 37946.69) | 0.13 (0.07 to 0.19) |
| Egypt | 1005603.24 (930867.53 to 1085942.4) | 33293.98 (30384.38 to 36520.68) | 2229778.18 (2060146.91 to 2408219.35) | 34418.61 (31470.15 to 37708.04) | 0.22 (0.17 to 0.28) |
| Iran (Islamic Republic of) | 999034.93 (886823.8 to 1125299.05) | 37970.84 (33305.34 to 42995.72) | 2991170.94 (2682370.37 to 3346275.18) | 37507.51 (32987.29 to 42547.34) | -0.08 (-0.13 to -0.04) |

**Supplementary Table 6** DALYs of IHD among older adults in 1990 and 2019 and the EAPC in different countries and territories

| DALY | 1990 |  | 2019 |  | 1990-2019 |
| --- | --- | --- | --- | --- | --- |
| Location | DALY Cases NO.(95%UI) | ASDR/100,000 (95% CI) | DALY Cases NO.(95%UI) | ASDR/100,000 (95% CI) | EAPC (95%CI) |
| Japan | 1447318.7 (1356141.62 to 1506054.05) | 6932.74 (6466.59 to 7248.49) | 1265972.79 (1099862.52 to 1362724.25) | 2602.06 (2301.07 to 2793.51) | -3.41 (-3.6 to -3.22) |
| Republic of Korea | 373599.36 (349468.15 to 393103.04) | 12828.33 (11343.55 to 14256.32) | 302034.12 (265463.78 to 340125.02) | 2730.93 (2247.91 to 3238.03) | -5.18 (-5.56 to -4.8) |
| France | 935728.94 (880325.98 to 984117.94) | 8375.83 (7692.82 to 9006.1) | 590660.29 (533369.18 to 637269.43) | 3208.59 (2796.43 to 3596.77) | -3.57 (-3.7 to -3.43) |
| Spain | 725914.47 (682593.66 to 755502.14) | 10099.42 (9224.33 to 10890.95) | 492061.92 (449171.46 to 535059.2) | 3840.59 (3394.3 to 4285.03) | -3.69 (-3.87 to -3.51) |
| Portugal | 212522.07 (202909.45 to 220707.63) | 11737.14 (10848.21 to 12571.6) | 129561.45 (118292.35 to 139080.52) | 3915.23 (3467.37 to 4346.64) | -4.35 (-4.58 to -4.13) |
| Taiwan (Province of China) | 149561.09 (142322.86 to 157259.02) | 8598.57 (7894.13 to 9289.27) | 193839.67 (156853.37 to 237921.29) | 3924.36 (3117.13 to 4911.82) | -2.71 (-2.88 to -2.53) |
| San Marino | 314.79 (270.83 to 362.81) | 7251.07 (5779.57 to 8922.07) | 345.82 (240.67 to 483.82) | 4055.94 (2632.94 to 5964.76) | -2.08 (-2.31 to -1.86) |
| Israel | 112697.78 (107948.01 to 116166.91) | 17689.03 (16602.77 to 18593.52) | 62538.04 (57308.99 to 66661.65) | 4185.87 (3714.32 to 4601.55) | -5.76 (-6.11 to -5.42) |
| Andorra | 481.23 (375.97 to 653.29) | 7481.09 (5516.95 to 10385.72) | 719.7 (547.86 to 913) | 4265.44 (3052.09 to 5677.25) | -2.14 (-2.36 to -1.92) |
| Netherlands | 391464.22 (373808.62 to 404882.44) | 15079.77 (14120.68 to 15907.44) | 193665.71 (178611.58 to 206714.61) | 4287.49 (3798.34 to 4766.98) | -5.1 (-5.43 to -4.78) |
| Switzerland | 182559.17 (173177.64 to 189529.39) | 13144.28 (12255.69 to 13894.16) | 103452.21 (92602.99 to 113875.01) | 4447.47 (3879.04 to 4980.19) | -4.2 (-4.37 to -4.02) |
| Peru | 133068.19 (114905.83 to 152654.84) | 10201.89 (8387.5 to 12158.6) | 169117.83 (127316.91 to 216678.22) | 4456.48 (3181.42 to 6012.54) | -2.9 (-3.25 to -2.56) |
| Australia | 451985.41 (430987.73 to 465227.96) | 17912.17 (16784.1 to 18757.66) | 250103.91 (227750.77 to 267116.63) | 4560.06 (4005.26 to 5028.22) | -5.28 (-5.55 to -5.01) |
| Italy | 1344940.54 (1289086.29 to 1428048.45) | 11343.94 (10799.01 to 12074.04) | 912939.25 (812674.63 to 981669.12) | 4587.37 (4103.51 to 4951.55) | -3.54 (-3.71 to -3.37) |
| Chile | 147177.35 (141332.1 to 151725.13) | 12751.37 (11845.08 to 13530.54) | 142411.62 (132295.52 to 151228.64) | 4769.82 (4253.22 to 5241.34) | -3.35 (-3.52 to -3.17) |
| Denmark | 223830.51 (214822.12 to 229908.07) | 20588.84 (19525.41 to 21429.19) | 73036.36 (67386.14 to 78245.3) | 4798.8 (4234.41 to 5335.61) | -5.85 (-6.16 to -5.53) |
| Luxembourg | 11351.14 (10817.59 to 11820.77) | 15835.4 (14683.26 to 16920.64) | 6226.92 (5413.88 to 7057.34) | 4872.04 (4141.63 to 5626.6) | -4.36 (-4.49 to -4.22) |
| Norway | 175466.71 (167831.91 to 180109.62) | 18992.17 (18097.96 to 19588.52) | 60803.93 (55893.84 to 65153.56) | 4896.69 (4479.37 to 5271.04) | -5.14 (-5.32 to -4.97) |
| Thailand | 303015.41 (267087.67 to 338766.37) | 8476.78 (6723.68 to 10412.43) | 604841.44 (463427.39 to 774955.4) | 4940.65 (3567.21 to 6544.51) | -2.31 (-2.47 to -2.15) |
| Belgium | 290271.91 (274549.4 to 300804.33) | 14124.46 (13071.61 to 15062) | 153237.33 (140813.87 to 163545.8) | 5018.41 (4465.47 to 5546.23) | -3.87 (-4.02 to -3.73) |
| Singapore | 39701.13 (38169.15 to 40962.3) | 16281.81 (15189.63 to 17238.86) | 46735.68 (42436.57 to 49916.97) | 5018.89 (4413.63 to 5536.29) | -4.44 (-4.64 to -4.25) |
| Slovenia | 41549.37 (32597.83 to 53002.01) | 13500.74 (10398.4 to 17736.17) | 32627.9 (26036.01 to 41745.6) | 5609.48 (4355.01 to 7223) | -3.75 (-4 to -3.5) |
| Panama | 20108.69 (18759.99 to 21128.16) | 11771.98 (10652.24 to 12692.48) | 28022.28 (22241.02 to 34907.85) | 5675.71 (4394.57 to 7150.44) | -2.23 (-2.48 to -1.98) |
| Iceland | 6347.12 (5968.43 to 6649.11) | 17221.9 (15944.67 to 18318.76) | 4064.42 (3647.37 to 4461.3) | 5695.75 (4981.85 to 6375.7) | -4.19 (-4.32 to -4.05) |
| Monaco | 1240.74 (1000.23 to 1460.52) | 12449.63 (9580.13 to 15351.1) | 735.39 (580.89 to 856.76) | 5737.23 (4265.45 to 7211.88) | -2.81 (-3.09 to -2.53) |
| Canada | 672865.21 (639746.55 to 693958.28) | 16221.18 (15143.85 to 17029.36) | 517408.74 (476399.76 to 548055.89) | 5784.02 (5143.04 to 6342.32) | -4.14 (-4.41 to -3.88) |
| Puerto Rico | 71510.32 (67906.3 to 74331.99) | 15488.16 (14449.63 to 16399.38) | 55892.61 (44660.46 to 68470.95) | 5933.91 (4668.18 to 7388.97) | -3.75 (-3.94 to -3.55) |
| United Kingdom | 2526284.41 (2421293.57 to 2580975.57) | 20755.89 (19854.68 to 21276.38) | 1009918.1 (945372.13 to 1052448.13) | 6138.01 (5729.1 to 6420.86) | -4.92 (-5.21 to -4.63) |
| New Zealand | 99386.46 (95398.4 to 102419.96) | 19438.09 (18295.05 to 20370.49) | 64266.58 (59177.11 to 68003.63) | 6314.83 (5651.93 to 6856.33) | -4.44 (-4.67 to -4.2) |
| Saint Lucia | 1565.24 (1461.86 to 1664.97) | 14499.51 (12863.39 to 16066.45) | 1620.28 (1393.88 to 1874.96) | 6357.55 (5241.47 to 7566.81) | -3.17 (-3.68 to -2.67) |
| Jamaica | 18652.59 (17262.16 to 20300.04) | 8110.05 (7181.29 to 9057.09) | 23122.06 (19036.22 to 27732.98) | 6538.03 (5206.55 to 8007.67) | -0.65 (-0.99 to -0.3) |
| Sweden | 395211.06 (376240.49 to 407392.54) | 18931.49 (17852.68 to 19733.79) | 185256.07 (170638.52 to 199631.46) | 6568.78 (5919.14 to 7197.17) | -3.96 (-4.09 to -3.83) |
| Ireland | 129999.38 (125072.19 to 134104.23) | 24084.15 (22918.44 to 25118.54) | 63369.78 (57035.83 to 67720.35) | 6604.13 (5830.73 to 7246.94) | -5.16 (-5.44 to -4.88) |
| Uruguay | 77637.68 (74296.65 to 80421.29) | 15269.13 (14158.08 to 16215.05) | 46844.42 (43768.41 to 49623.54) | 6640.74 (5971.89 to 7300.09) | -2.99 (-3.15 to -2.84) |
| Ecuador | 56201.13 (52785.27 to 59251.02) | 9425.31 (8454.62 to 10341.82) | 121303.14 (97996.66 to 151435.17) | 6828.3 (5363.19 to 8624.65) | -1 (-1.28 to -0.72) |
| Germany | 3403583.69 (3237240.59 to 3520515.81) | 20131.91 (18786.17 to 21183.77) | 1839669.75 (1701665.49 to 1950181.21) | 6929.41 (6244.51 to 7566.25) | -4.08 (-4.38 to -3.79) |
| Barbados | 4878.5 (4549.51 to 5157.54) | 12068.5 (10941.96 to 13117.71) | 4390.54 (3734.82 to 5092.1) | 6937.97 (5738.07 to 8227.81) | -2.38 (-2.65 to -2.11) |
| Austria | 263688.27 (249516.21 to 283560.52) | 16355.76 (15225.85 to 17751.06) | 166938.99 (153784.77 to 178584.47) | 7011.84 (6287.65 to 7680.89) | -3.47 (-3.68 to -3.26) |
| Costa Rica | 26365.01 (24767.03 to 27698.23) | 13132.53 (11993.44 to 14067.69) | 43705.82 (35221.13 to 54737.81) | 7168.9 (5626.93 to 9008.95) | -2.44 (-2.68 to -2.2) |
| Bermuda | 1882.87 (1776.06 to 1979.5) | 24937.66 (23100.11 to 26747.1) | 1225.19 (1051.55 to 1447.11) | 7193.54 (6012.32 to 8613.51) | -4.44 (-4.83 to -4.05) |
| Colombia | 293764.38 (279409.26 to 311268.52) | 15478.27 (14368.59 to 16564.78) | 478793.63 (383334.94 to 598572.45) | 7512.44 (5829.11 to 9483.46) | -2.67 (-2.8 to -2.53) |
| Greece | 273113.68 (261266.83 to 283823.07) | 13713.68 (12749.8 to 14608.93) | 252469.21 (231606.47 to 267999.88) | 7692.01 (6931.99 to 8370.21) | -2.37 (-2.51 to -2.24) |
| Argentina | 638952.76 (612762.66 to 659869.04) | 16098.75 (14874.56 to 17199.82) | 529396.79 (493183.04 to 561419.87) | 7719.79 (6893.46 to 8518.61) | -2.53 (-2.72 to -2.33) |
| Brazil | 1508824.12 (1441905.44 to 1559884.1) | 15330.57 (14440.39 to 16039.84) | 2183442.44 (2034029.15 to 2294298.48) | 7720.99 (7086.77 to 8237.51) | -2.3 (-2.38 to -2.21) |
| Cyprus | 19129.92 (17689.13 to 21248.88) | 20224.89 (17623.76 to 23494.16) | 19695.97 (17386.98 to 23272.01) | 7833.88 (6623.29 to 9454.76) | -3.95 (-4.16 to -3.74) |
| South Africa | 172915.67 (148636.79 to 191485.19) | 7438.65 (6291.41 to 8420.56) | 390476.56 (355886.37 to 419799.43) | 7839.74 (6982.58 to 8612.6) | 0.18 (-0.35 to 0.71) |
| Guatemala | 56075.82 (50441.1 to 61882.2) | 15327.69 (13489.45 to 17196.68) | 105084.17 (85667.67 to 127545.65) | 8144.46 (6486.77 to 9951.22) | -2.37 (-2.87 to -1.88) |
| United States of America | 7567179.32 (7203091.44 to 7765305.92) | 17990.07 (17079.55 to 18565.29) | 5949488.69 (5595795.07 to 6230070.75) | 8299.37 (7739.11 to 8733.64) | -3.13 (-3.35 to -2.92) |
| Malta | 12025.27 (11503.69 to 12558.99) | 22383.27 (20905.34 to 23740.57) | 10571.16 (9486.76 to 11667.06) | 8406.55 (7365.04 to 9408) | -3.43 (-3.52 to -3.33) |
| Belize | 1592.66 (1477.27 to 1706.69) | 14508.05 (13043.27 to 15963.72) | 2512 (2211.12 to 2842.15) | 8600.38 (7311.74 to 9955.28) | -2.39 (-2.87 to -1.91) |
| Kenya | 59838.31 (50500.41 to 71612.23) | 7042.92 (5867.73 to 8467.56) | 184310.93 (145922.42 to 226852.28) | 8671.9 (6760.33 to 10784.11) | 0.94 (0.74 to 1.14) |
| Antigua and Barbuda | 946.44 (879.4 to 1010.12) | 13588.04 (11972.49 to 15204.33) | 995.97 (856.27 to 1128.98) | 8676.47 (7146.11 to 10293.68) | -1.77 (-1.99 to -1.55) |
| Rwanda | 36488.55 (28790.09 to 46540.31) | 11891.29 (8456.91 to 16370.95) | 51726.82 (38521.35 to 67808.29) | 8711.09 (5884.11 to 12189.56) | -1.58 (-1.79 to -1.38) |
| Finland | 217338 (209130.97 to 223739.15) | 23203.95 (21971.3 to 24217.27) | 145033.32 (134537.5 to 155611.15) | 8718.63 (7835.48 to 9498.75) | -3.64 (-3.79 to -3.49) |
| Dominica | 1447.85 (1308.78 to 1578.35) | 14587.2 (12424.27 to 16831.11) | 1001.51 (844.03 to 1190.58) | 8817.66 (6791.07 to 11208.31) | -1.79 (-2.04 to -1.54) |
| Mexico | 499059.44 (474720.73 to 516157.45) | 10679.56 (10074.39 to 11105.86) | 1203318.8 (1052338.93 to 1364518.02) | 8931.35 (7668.49 to 10194.54) | -0.69 (-0.85 to -0.52) |
| Ethiopia | 250534.55 (196025.77 to 323231.99) | 11944.11 (8950.29 to 15687.52) | 379962.12 (286865.26 to 472936.2) | 8940.04 (6527.74 to 11449.83) | -1.19 (-1.26 to -1.11) |
| South Sudan | 24421.9 (18886.8 to 30256.46) | 9531.7 (6706.57 to 12784.93) | 32572.08 (23405.02 to 43300.41) | 8987.21 (6000.1 to 12654.35) | -0.08 (-0.17 to 0.01) |
| Uganda | 59232.44 (44548.96 to 74114.03) | 8770.83 (5963.66 to 11962.78) | 122952.85 (85997.15 to 154679.76) | 8988.4 (5891.99 to 12182.2) | -0.11 (-0.34 to 0.13) |
| Paraguay | 27376.72 (23971.53 to 30375.11) | 10924.49 (9297 to 12460.83) | 57971.51 (45865.7 to 73399.87) | 9147.88 (6915.64 to 11895.72) | -0.49 (-0.77 to -0.22) |
| Bahamas | 2438.46 (2258.03 to 2631.95) | 14306.2 (12662.21 to 15955.79) | 3969.19 (3326.3 to 4750.88) | 9151.96 (7386.84 to 11159.72) | -1.76 (-1.97 to -1.54) |
| Zambia | 32439.01 (27142.9 to 39031.83) | 11223.25 (8270.48 to 14817.92) | 59259.91 (47733.5 to 72777.22) | 9213.77 (6707.85 to 12276.43) | -0.94 (-1.23 to -0.65) |
| Viet Nam | 491822.65 (408842.45 to 583220.47) | 10576.25 (7939.81 to 13684.79) | 931351.67 (755193.79 to 1109265.75) | 9416.26 (7019.13 to 12063.63) | -0.49 (-0.62 to -0.35) |
| El Salvador | 40676.45 (37897.95 to 43231.75) | 12112.52 (10797.76 to 13334.32) | 69684.4 (54642.36 to 87217.94) | 9585.88 (7275.36 to 12258.72) | -0.92 (-1.06 to -0.78) |
| Malawi | 43050.4 (35788.39 to 50936.46) | 10494.93 (7868.34 to 13543) | 72930.34 (57938.75 to 90001.99) | 9587.58 (6969.41 to 12588.72) | -0.42 (-0.59 to -0.25) |
| Greenland | 668.41 (604.92 to 734.28) | 20366.59 (17413.13 to 23576.42) | 741.78 (618.26 to 868.28) | 9608.49 (7566.35 to 11767.6) | -3.12 (-3.36 to -2.88) |
| Cook Islands | 196.75 (169.02 to 232.31) | 13755.43 (10655.65 to 17432.45) | 297 (254 to 349.77) | 9664.1 (7563.6 to 12033.42) | -1.31 (-1.48 to -1.14) |
| United Republic of Tanzania | 105312.12 (86409.12 to 126882.15) | 9216.95 (6812.49 to 11927.64) | 246164.1 (183508.67 to 310017.02) | 9886.48 (6777.73 to 13439.49) | 0.28 (0.25 to 0.32) |
| Mauritius | 23487.27 (22488.39 to 24421.61) | 27449.69 (25510.82 to 29270.53) | 20752.53 (17303.68 to 24841.1) | 9927.3 (8012.34 to 12098.14) | -4.47 (-4.93 to -4) |
| Equatorial Guinea | 3220.64 (2367.55 to 4257.24) | 15417.4 (10485.6 to 21720.75) | 4627.79 (3355.86 to 6347.47) | 10039.44 (6660.61 to 14326.52) | -1.92 (-2.17 to -1.68) |
| China | 8255867.47 (7341383.87 to 9193067.2) | 9292.59 (8201.17 to 10477.16) | 23853563.12 (20716429.07 to 27039355.49) | 10248.09 (8811.76 to 11651.69) | 0.96 (0.69 to 1.24) |
| Myanmar | 372263.53 (295981.91 to 461426.68) | 14594.22 (10753 to 19202.22) | 518882.37 (454920.33 to 598817.5) | 10337.02 (8391.2 to 12618.14) | -1.37 (-1.44 to -1.3) |
| Bolivia (Plurinational State of) | 49698.87 (36571.81 to 65292.15) | 14342.53 (10018.14 to 19537.96) | 103227.66 (73423.23 to 135447.04) | 10387.59 (7038.16 to 14424.77) | -1.17 (-1.41 to -0.92) |
| Sri Lanka | 195300.29 (178807.49 to 213488.47) | 16922.38 (14613.47 to 19428.96) | 326182.6 (243222.66 to 417673.32) | 10628.42 (7721.02 to 13976.23) | -1.34 (-1.52 to -1.17) |
| Seychelles | 1030.85 (944.93 to 1126.06) | 14571.81 (12463.76 to 16776.93) | 1296.44 (1141.79 to 1454.9) | 10663.07 (8917.71 to 12557.4) | -1.33 (-1.5 to -1.16) |
| Nigeria | 614220.65 (460471.58 to 838455.01) | 13147.65 (9764.84 to 18047.67) | 889796.19 (653191.62 to 1130900.78) | 10744.59 (7772.07 to 13596.39) | -0.81 (-0.93 to -0.69) |
| Saint Kitts and Nevis | 1281.35 (1195.32 to 1364.66) | 24494.9 (21953.28 to 27105.36) | 767.97 (687.65 to 865.45) | 10759.52 (9178.01 to 12493.16) | -2.98 (-3.25 to -2.71) |
| Mozambique | 54229.3 (44114.98 to 65612.91) | 8777.85 (6386.8 to 11718.93) | 119632.27 (95389.15 to 150837.31) | 10798.09 (7683.77 to 14606.74) | 1.17 (0.97 to 1.37) |
| Mauritania | 17791.88 (15035.43 to 20972.69) | 15873.55 (12101.29 to 20162.98) | 24446.08 (19287.45 to 30451.57) | 10812.29 (7863.46 to 14125.7) | -1.22 (-1.38 to -1.06) |
| Comoros | 2923.25 (2016.45 to 3641.56) | 11771.89 (7715.99 to 15914.44) | 5808.41 (4463.82 to 7466.15) | 10832.1 (7600.11 to 14713.42) | -0.34 (-0.41 to -0.26) |
| Kuwait | 10718.76 (9935.86 to 11523.73) | 19814.01 (17808.09 to 21651.76) | 24381.18 (20457.21 to 28997.48) | 10861.3 (8906.89 to 13018.95) | -2 (-2.33 to -1.67) |
| Cuba | 258284.19 (244946.96 to 266730.47) | 20018.65 (18805.74 to 20950.97) | 256269.29 (215007.43 to 306684.95) | 10967.86 (9002.33 to 13239.72) | -2.43 (-2.72 to -2.15) |
| Suriname | 5377.79 (4988.31 to 5698.32) | 18526.27 (16476.6 to 20404.28) | 7596.28 (6447.51 to 8857.51) | 11007.75 (8757.21 to 13563.47) | -1.93 (-2.33 to -1.54) |
| Bangladesh | 591758.08 (502841.83 to 677093.08) | 11570.6 (9126.87 to 14235.27) | 1712713.22 (1342566.95 to 2105629.98) | 11153.4 (8331.17 to 14297.42) | 0.45 (0.11 to 0.78) |
| Grenada | 1883.8 (1747.53 to 2020.48) | 19617.94 (17465.85 to 21657.35) | 1462.43 (1344.61 to 1584.79) | 11420.86 (10077.43 to 12779.63) | -1.95 (-2.22 to -1.68) |
| Eritrea | 8544.11 (6086.07 to 11588.68) | 9874.69 (6354.37 to 14761.87) | 27651.32 (21771.29 to 34699.47) | 11521.53 (8309.02 to 15540.54) | 0.58 (0.52 to 0.65) |
| Poland | 1696893.22 (1628941.5 to 1736759.61) | 30901.82 (29416.97 to 31836.91) | 1090603.24 (932351.7 to 1263711.58) | 11576.1 (9713 to 13566.68) | -3.78 (-3.98 to -3.57) |
| Turkey | 946418.96 (830314.3 to 1058378.77) | 23879.14 (19874.34 to 27962.03) | 1211776.23 (982212.83 to 1466520.13) | 11764.09 (9263.78 to 14642.38) | -2.7 (-2.98 to -2.43) |
| Cambodia | 60069.28 (49621.05 to 74543.01) | 12750.24 (9601.69 to 16700.14) | 149452.39 (121231.3 to 178047.84) | 11802.87 (8943.69 to 15085.16) | -0.35 (-0.4 to -0.3) |
| Namibia | 10954.12 (9138.39 to 12904.44) | 12985.85 (9820.41 to 16538.52) | 17932.65 (14359.15 to 21497.1) | 11803.75 (8674.66 to 15485.8) | -0.45 (-0.63 to -0.27) |
| Mali | 55621.28 (45814.13 to 66654.02) | 13083.12 (9729.79 to 16866.79) | 102920.93 (82321.92 to 127736.76) | 11867.8 (8670.11 to 15540.91) | -0.26 (-0.36 to -0.16) |
| Burundi | 35874.82 (27806.13 to 45831.18) | 13952.91 (9898.8 to 19118.29) | 52745.79 (39410.11 to 68559.31) | 11897.89 (8334.2 to 16312.41) | -0.67 (-0.73 to -0.61) |
| Benin | 29879.23 (25511.75 to 34634.38) | 13310.43 (10183.2 to 16825) | 58808.06 (47322.28 to 72231.77) | 11905.75 (8839.16 to 15399.22) | -0.32 (-0.38 to -0.25) |
| Croatia | 194444.44 (183405.76 to 204915.07) | 25126.14 (23006.9 to 26997.03) | 147059.78 (119577.38 to 178514.65) | 11910.15 (9507.17 to 14646.7) | -2.59 (-2.71 to -2.48) |
| Eswatini | 3456.69 (2857.84 to 4118.69) | 11702.68 (8772.37 to 15066.07) | 7176.77 (5503.4 to 9309.38) | 11930.3 (8483.12 to 16291.42) | 0.41 (0.16 to 0.67) |
| Brunei Darussalam | 1861.93 (1662.36 to 2072.58) | 20690.22 (17539.86 to 24126.34) | 3105.44 (2754.28 to 3494.14) | 11940.3 (10086.67 to 13917.55) | -1.75 (-1.91 to -1.6) |
| Angola | 50619.16 (39899.64 to 62345.78) | 13490.98 (9733.67 to 18014.86) | 122936.88 (95267 to 154543.48) | 11970.34 (8472.45 to 15932.81) | -0.61 (-0.71 to -0.5) |
| Democratic People's Republic of Korea | 164097.62 (128535.3 to 200943.02) | 10341.94 (7389.27 to 13637.67) | 450732.57 (380717.39 to 530190.6) | 11988.21 (9228.48 to 14999.85) | 0.71 (0.49 to 0.92) |
| Maldives | 2490.34 (2212.34 to 2828.24) | 28214.7 (23622.22 to 33349.69) | 3539.87 (2969.97 to 4129.97) | 12030.51 (9667.76 to 14579.35) | -3.7 (-3.93 to -3.46) |
| Liberia | 18371.62 (15374.94 to 21849.52) | 14238.46 (10871.68 to 18156.78) | 23494.47 (18242.81 to 30120.62) | 12040.17 (8709.53 to 16051.67) | -0.43 (-0.57 to -0.28) |
| Senegal | 47301.52 (38482.12 to 54742.78) | 13637.36 (10218.32 to 17271.66) | 95680.83 (76004.82 to 116310.7) | 12090.34 (8878.46 to 15758.87) | -0.32 (-0.42 to -0.21) |
| Djibouti | 1233.61 (975.95 to 1577.61) | 10411.97 (7553.62 to 14034.84) | 6549.03 (4748.89 to 8877.96) | 12097.22 (8219.46 to 16863) | 0.59 (0.52 to 0.67) |
| Cameroon | 49057.37 (38841.33 to 60235.72) | 11000.3 (7976 to 14599.9) | 142676.51 (112837.62 to 181762.22) | 12103.68 (8774.56 to 16228.42) | 0.52 (0.32 to 0.73) |
| Saint Vincent and the Grenadines | 1699.28 (1584.62 to 1808.93) | 18599.83 (16695.3 to 20444.33) | 1974.86 (1756.46 to 2220.93) | 12126.2 (10379.35 to 14010.41) | -1.58 (-1.92 to -1.24) |
| Gabon | 8905.96 (7302.86 to 10781.4) | 13994.99 (10347.72 to 18318.24) | 12961.37 (10133.17 to 15784.87) | 12159 (8744.97 to 16104.96) | -0.46 (-0.55 to -0.37) |
| Democratic Republic of the Congo | 225173.59 (179296.24 to 290934.38) | 13915.62 (10251.95 to 18684.28) | 417889.61 (305924.5 to 562493.71) | 12190.77 (8318.04 to 17000.91) | -0.5 (-0.56 to -0.44) |
| Jordan | 29723.27 (25983.5 to 33600.36) | 23225.92 (19118.22 to 27598.72) | 77492.87 (65694.55 to 91849.86) | 12207.64 (9903.34 to 14906.21) | -2.95 (-3.26 to -2.63) |
| Trinidad and Tobago | 22943 (21998.03 to 23939.48) | 22550.56 (21000.3 to 24034.6) | 28266.68 (21919.29 to 35965.02) | 12301.46 (9461.92 to 15679.99) | -2.83 (-3.12 to -2.53) |
| Lesotho | 9827.13 (7592.33 to 12062) | 8891.94 (6254.08 to 11937.43) | 16747.94 (12582.6 to 21903.4) | 12302.19 (8444.43 to 16942.35) | 1.65 (1.4 to 1.9) |
| Niger | 37711.48 (29563.87 to 46077.48) | 13894.78 (10024.37 to 18413.07) | 95058.63 (74937.58 to 121117.09) | 12390.81 (8987.3 to 16552.72) | -0.34 (-0.45 to -0.22) |
| Estonia | 89795.96 (86304.28 to 92587.59) | 34457.95 (32559.68 to 36001.8) | 46491.62 (36612.77 to 62221.2) | 12535.33 (9613.59 to 16920.67) | -4.31 (-4.68 to -3.93) |
| Nepal | 110104.99 (87017.93 to 139329.36) | 11159.61 (8176.1 to 14843.31) | 321047.49 (253081.25 to 387933.62) | 12578.37 (9273.18 to 16197.73) | 0.51 (0.42 to 0.6) |
| Chad | 39253.17 (32335.42 to 47131.61) | 12162.88 (9071.65 to 15703.55) | 72958.74 (60168.08 to 89228.1) | 12791.74 (9552.65 to 16744.66) | 0.27 (0.21 to 0.33) |
| Ghana | 78216.91 (64743.92 to 92640.81) | 12539.99 (9519.2 to 15972.94) | 211059.56 (174898.85 to 252480.86) | 12991.83 (9939.38 to 16569.62) | 0.38 (0.29 to 0.48) |
| Tonga | 837.23 (706.47 to 964.61) | 13356.25 (10467.35 to 16551.13) | 1198.79 (987.67 to 1427.55) | 12993.49 (10067.22 to 16288.21) | -0.1 (-0.31 to 0.11) |
| Somalia | 27623.12 (21009.09 to 34847.56) | 12316.8 (8559.53 to 16794.3) | 84053.45 (61734.83 to 112313.53) | 13004.18 (8994.03 to 18336.35) | 0.52 (0.39 to 0.64) |
| Guinea | 42954.9 (34698.72 to 52909.94) | 11365.25 (8239.07 to 14910.72) | 77260.57 (62221.99 to 95561.18) | 13013.16 (9671.38 to 17139.48) | 0.87 (0.73 to 1.01) |
| Madagascar | 67557.54 (57480.16 to 79121.56) | 12547.83 (9932.3 to 15471.07) | 128298.51 (93148.43 to 170715.02) | 13026.55 (8868.06 to 17998.77) | 0.07 (-0.07 to 0.21) |
| Czechia | 596557 (572849.17 to 612956.2) | 33046.56 (31289.76 to 34496.04) | 369844.69 (309185.66 to 436178.77) | 13138.33 (10802.27 to 15649.02) | -3.37 (-3.49 to -3.26) |
| Venezuela (Bolivarian Republic of) | 191262.06 (181743.51 to 200701.05) | 17875.77 (16552.83 to 19063.52) | 448084.31 (349293.81 to 567724.41) | 13199.67 (10209.06 to 16806.24) | -1.39 (-1.57 to -1.22) |
| Botswana | 7843.52 (6203.59 to 9701.63) | 12674.73 (9323.15 to 16786.86) | 18331.09 (13867.25 to 23511.87) | 13330.58 (9418.1 to 17998.98) | -0.03 (-0.28 to 0.23) |
| Bhutan | 3029.89 (2233.4 to 3989.06) | 12287.17 (8638.99 to 16678.87) | 8479.58 (6705.5 to 10411.2) | 13414.09 (9905.4 to 17248.69) | 0.35 (0.28 to 0.43) |
| Indonesia | 1173558.56 (1036755.2 to 1311141.82) | 11755.93 (10170.31 to 13392.61) | 3066681.25 (2602380.59 to 3395950.85) | 13700.47 (11602.43 to 15433.47) | 0.69 (0.59 to 0.78) |
| Nicaragua | 17627 (16220.66 to 18949.99) | 10862.42 (9339.33 to 12338.96) | 64669.21 (55079.23 to 74578.45) | 13789.24 (11212.82 to 16447.37) | 0.52 (0.2 to 0.84) |
| Albania | 36870.11 (34813.48 to 38853.03) | 16749.53 (15116.56 to 18337.38) | 76731.27 (59905.1 to 98028.13) | 13805.57 (10534.26 to 17897.58) | -0.37 (-0.56 to -0.17) |
| Sierra Leone | 33362.6 (26262.12 to 41207.1) | 15221.61 (10981.64 to 20031.5) | 51754.34 (39495.03 to 66531.55) | 13911.23 (9898.35 to 18820.87) | -0.03 (-0.16 to 0.11) |
| Togo | 17975.47 (14953.56 to 21332.23) | 14720.2 (11303.59 to 18805.19) | 48728.83 (39304.53 to 60977.81) | 13943.6 (10330.97 to 18345.7) | -0.11 (-0.17 to -0.05) |
| Philippines | 254927.45 (222381.3 to 290949.7) | 8663.59 (7502.86 to 10013.81) | 1149987.5 (965961.05 to 1348423.56) | 13953.45 (11605.91 to 16371.12) | 2.18 (1.7 to 2.67) |
| Cabo Verde | 3829.36 (3365.04 to 4239.38) | 12550.41 (10312.44 to 14864.88) | 6598.78 (5688.4 to 7406.63) | 13954.58 (11131.92 to 16866.87) | -0.41 (-0.79 to -0.02) |
| Burkina Faso | 53074.19 (43377.62 to 63264.59) | 11316.6 (8344.83 to 14554.23) | 128727.68 (106034.84 to 151496.74) | 14033.06 (10666.88 to 17714.03) | 1.07 (0.85 to 1.3) |
| Bosnia and Herzegovina | 98100.51 (92707.13 to 102586.19) | 23161.82 (21081.84 to 25028.5) | 112796.7 (92751.78 to 136963.92) | 14596.71 (11570.02 to 18067.05) | -2.15 (-2.33 to -1.96) |
| American Samoa | 344.77 (309.32 to 384.79) | 14807.2 (12031.26 to 17943.81) | 781.07 (684.79 to 879.96) | 14673.98 (11936.71 to 17519.51) | 0.04 (-0.02 to 0.1) |
| Congo | 20787.22 (16610.98 to 25939.6) | 18136.36 (13315.31 to 24038.57) | 36209.99 (27694.14 to 47485.15) | 14692.3 (10291.31 to 19919.38) | -0.84 (-0.93 to -0.75) |
| Bahrain | 7277.47 (6403.59 to 8163.74) | 45715.69 (39798.16 to 51867.1) | 11064.83 (8933.54 to 13513.55) | 14737.66 (11744.48 to 18211.28) | -4.46 (-4.8 to -4.12) |
| India | 7874230.97 (6970541.74 to 8788095.47) | 17146.57 (14978.17 to 19435.16) | 19333697.5 (16777734.93 to 22127118.64) | 14740.05 (12628.72 to 16937.74) | -0.56 (-0.67 to -0.46) |
| Northern Mariana Islands | 158.05 (134.58 to 187.17) | 11787.14 (9036.22 to 14909.79) | 813.48 (707.15 to 920.67) | 14767.77 (12061.6 to 17694.05) | 1.25 (1.07 to 1.43) |
| Sao Tome and Principe | 866.94 (725.59 to 1000.25) | 11738.22 (9082.74 to 14608.45) | 1551.12 (1256.42 to 1817.44) | 14911.65 (11321.41 to 18622.87) | 0.83 (0.71 to 0.94) |
| Honduras | 26816.89 (22145.65 to 35470.58) | 11943.14 (9255.12 to 16064.6) | 101724.55 (83805.21 to 125598.33) | 15167.36 (11687.87 to 19713.46) | 1.02 (0.81 to 1.24) |
| Iran (Islamic Republic of) | 750015.93 (697660.32 to 806179.98) | 28494.98 (25842.46 to 30876.61) | 1218784.98 (1135788.81 to 1317093.96) | 15363.47 (14106.77 to 16702.94) | -2.7 (-2.98 to -2.41) |
| Malaysia | 183188.62 (168992.43 to 197137.62) | 18648.03 (15828.62 to 21626.37) | 470754.99 (378272.36 to 573682.67) | 15422.5 (11928.11 to 19402.98) | -1.4 (-1.75 to -1.04) |
| Timor-Leste | 2677.53 (2172.65 to 3336.54) | 11183.72 (8458.34 to 14663.85) | 14640.38 (11172.72 to 17985.33) | 15497.85 (11270.49 to 19900.74) | 1.26 (1.15 to 1.37) |
| Central African Republic | 19451.15 (15425.74 to 24268.78) | 16529.6 (11820.54 to 22205.27) | 30714.35 (22821.48 to 40679.45) | 15578.23 (10687.27 to 21615.5) | -0.08 (-0.15 to -0.01) |
| Gambia | 4896.88 (3904.39 to 6057.15) | 13686.77 (9979.53 to 18028.66) | 16062.67 (12956.58 to 19297.97) | 15641.44 (11689.97 to 19957.23) | 0.53 (0.4 to 0.66) |
| Hungary | 514746.12 (495740.28 to 529982.15) | 26995.91 (25366.09 to 28487.71) | 415421.43 (351645.65 to 486576.56) | 15799.78 (13091.89 to 18724.78) | -2.03 (-2.13 to -1.93) |
| Romania | 922974.89 (887101.72 to 964806.21) | 27671.66 (25802.68 to 29463.67) | 814445.69 (689613.08 to 954194.7) | 15852.46 (13079.05 to 18950.71) | -2.53 (-2.77 to -2.29) |
| Lao People's Democratic Republic | 40102.97 (31913.01 to 50406.47) | 17779.66 (13200.79 to 23656.29) | 70269.33 (56525.49 to 84142.85) | 15934.69 (12189.76 to 19872.4) | -0.49 (-0.55 to -0.43) |
| Montenegro | 11413.36 (10367.1 to 12541.93) | 15662.83 (13401.87 to 18015.06) | 20572.8 (17397.41 to 24085.75) | 16076.16 (13135.42 to 19412.39) | 0.23 (0.05 to 0.42) |
| Dominican Republic | 52094.72 (46363.25 to 57879.78) | 12643.12 (10800.91 to 14493.44) | 180971.69 (143670.02 to 228186.29) | 16799.09 (12779.37 to 21738.54) | 1.67 (1.43 to 1.9) |
| Zimbabwe | 59757.97 (52754.13 to 67014.25) | 13944.29 (11207.25 to 16872.64) | 122310.04 (98560.94 to 148199.57) | 16889.45 (12778.89 to 21592.03) | 1.02 (0.85 to 1.19) |
| Guam | 1542.56 (1364.08 to 1752.33) | 19710.75 (16332.84 to 23401.84) | 3826.38 (3257.87 to 4496.34) | 16963.4 (14029.73 to 20229.86) | -0.58 (-0.91 to -0.24) |
| Serbia | 314679.92 (290399.2 to 341415.99) | 24064.74 (21039.76 to 27020.35) | 370124.92 (306361.35 to 443714.42) | 17106.93 (13762.91 to 20902.98) | -1.64 (-1.92 to -1.35) |
| United States Virgin Islands | 1848.95 (1613.4 to 2092.47) | 20003.27 (16843.11 to 23461.27) | 4219.55 (3742.77 to 4647.27) | 17116.82 (14309.73 to 19894.82) | -0.23 (-0.46 to 0) |
| Guinea-Bissau | 7702.42 (6011.94 to 9829.61) | 17834.09 (12733.3 to 24003.8) | 12108.63 (9671.78 to 15121.31) | 17189.81 (12835.79 to 22331.38) | 0.12 (0.02 to 0.21) |
| Papua New Guinea | 26611.49 (18572.6 to 38325.1) | 13813.81 (9058.41 to 20307.66) | 77989.94 (56683.53 to 103860.53) | 17300.95 (12050.63 to 24076.76) | 0.96 (0.82 to 1.1) |
| Libya | 38533.19 (31396.01 to 47801.93) | 19496.81 (15197.49 to 24720.3) | 89024.89 (70783.13 to 115430.37) | 17445.75 (13232.78 to 22927.64) | -0.44 (-0.63 to -0.25) |
| United Arab Emirates | 8237.3 (6840.81 to 10421.58) | 30816.37 (24327.4 to 39388.87) | 36753.27 (27242.88 to 48253.26) | 17750.21 (12750.33 to 23448.34) | -2.14 (-2.7 to -1.57) |
| Slovakia | 274996.07 (261698.27 to 285482.83) | 36097.34 (33366.89 to 38428.78) | 211299.59 (169727.47 to 257080.98) | 17790.28 (14082.24 to 21852.5) | -2.64 (-2.86 to -2.42) |
| Tokelau | 34.78 (28.43 to 42.96) | 19168.38 (14547.39 to 25021) | 28 (23.09 to 34.01) | 18089.19 (14120.48 to 22857.66) | -0.18 (-0.28 to -0.07) |
| North Macedonia | 49038.2 (46314.73 to 51734.4) | 23235.46 (20858.17 to 25619.87) | 70999.9 (58017.59 to 86484.19) | 18286.63 (14513.32 to 22637.04) | -1.27 (-1.52 to -1.01) |
| Pakistan | 930151.97 (776755.8 to 1079168.29) | 14573.41 (11546.57 to 17738.02) | 2085594.04 (1751405.41 to 2508317.63) | 18467.68 (15016.88 to 22647.76) | 0.71 (0.47 to 0.95) |
| Latvia | 143682.56 (138413.6 to 147564.11) | 31342.59 (29726.21 to 32717.22) | 102396.96 (88126.52 to 119984.51) | 18558.84 (15500.53 to 22188.02) | -2.18 (-2.46 to -1.9) |
| Tunisia | 132705.99 (116275.27 to 152114.38) | 23693.13 (19870.09 to 27890.09) | 274587.61 (207534.34 to 352851.63) | 18778.79 (13850.06 to 24459.94) | -1.01 (-1.11 to -0.92) |
| Lithuania | 184922.44 (176300.38 to 190479.33) | 32250.7 (30475.01 to 33522.41) | 153749.88 (129633.79 to 182563.63) | 19153.89 (15825.58 to 22807.59) | -1.95 (-2.15 to -1.74) |
| Guyana | 11891.55 (10589.18 to 13158.47) | 28247.68 (24539.3 to 32034.52) | 13564.37 (10875.98 to 16737.09) | 19494.93 (15335.34 to 24192.92) | -1.17 (-1.32 to -1.01) |
| Algeria | 423446.73 (352123.53 to 502436.18) | 34868.01 (28636.88 to 41690.4) | 720519.05 (583689.81 to 886951.04) | 19965.47 (15697.94 to 24773.25) | -2.11 (-2.21 to -2.01) |
| Haiti | 91812.4 (76559.82 to 114281.01) | 25894.01 (19904.92 to 32893.66) | 147941.12 (108334.3 to 201711.01) | 20256.54 (14191.91 to 27928.78) | -0.72 (-0.85 to -0.6) |
| Niue | 58.81 (50.56 to 67.8) | 21326.74 (16911.78 to 26095.32) | 55.03 (44.96 to 63.02) | 20520.18 (15876.95 to 24749.01) | -0.24 (-0.33 to -0.16) |
| Bulgaria | 513583.84 (478587.31 to 537876.87) | 34321.85 (31636.44 to 36580.91) | 419053.61 (347302.59 to 498929.2) | 20752.26 (16694.18 to 25153.32) | -2.66 (-3.06 to -2.26) |
| Georgia | 294516.93 (274400.29 to 314975.86) | 39767.49 (36517.54 to 42833.04) | 163308.5 (140561.9 to 188826.7) | 20873.52 (17372.44 to 24647.33) | -2.83 (-3.14 to -2.52) |
| Palau | 266.16 (214.76 to 340.39) | 23254.31 (17633.39 to 30325.07) | 479.79 (392.32 to 590.82) | 21019 (16284.83 to 26622.73) | -0.35 (-0.41 to -0.3) |
| Saudi Arabia | 147305.74 (119484.2 to 175455.63) | 25448.92 (20051.52 to 31533.33) | 307363.7 (257482.91 to 356506.87) | 21032.21 (17051.66 to 24980.45) | -0.62 (-0.86 to -0.38) |
| Samoa | 2208.04 (1872.57 to 2590.82) | 22016.21 (17212.19 to 27191.57) | 3351.72 (2891.35 to 3983.44) | 21064.45 (17014.31 to 25965.21) | -0.13 (-0.15 to -0.1) |
| Palestine | 30403.77 (24349.98 to 37356.57) | 31151.58 (24423.42 to 38641.42) | 49724.78 (43384.66 to 56941.89) | 21470.31 (18149.86 to 25242.85) | -1.66 (-1.8 to -1.52) |
| Armenia | 90684.02 (87078.91 to 93851.55) | 31642.53 (29804.53 to 33261.85) | 112460.16 (96345.61 to 129955.16) | 21483.35 (18116.85 to 24980.96) | -2.05 (-2.25 to -1.85) |
| Kiribati | 986.93 (811.45 to 1166.43) | 23912.09 (18215.14 to 30416.92) | 1622.45 (1281.41 to 2006.91) | 22715.28 (16711.39 to 29650.02) | -0.2 (-0.25 to -0.14) |
| Kazakhstan | 411751.52 (392861.03 to 427756.8) | 29228.94 (27208.06 to 30953.64) | 419768.46 (365443.55 to 473444.56) | 22924.06 (19607.59 to 26344.03) | -1.57 (-2.29 to -0.85) |
| Qatar | 3149.31 (2548.2 to 3759.73) | 43765.61 (34298.74 to 52643.39) | 10153.9 (7780.4 to 12889.54) | 22965.92 (17981.12 to 28800.27) | -2.45 (-2.79 to -2.1) |
| Russian Federation | 6614864.38 (6413956.69 to 6735497.08) | 30067.51 (28821.6 to 30794.94) | 7006488.16 (6150809.97 to 7891285.6) | 23008.34 (19920.73 to 26043.1) | -1.29 (-1.77 to -0.8) |
| Tuvalu | 202.77 (164.68 to 247.82) | 24765.78 (18815.23 to 31976.87) | 281.43 (226.6 to 359.85) | 23378.21 (17870.43 to 30601.6) | -0.19 (-0.23 to -0.16) |
| Lebanon | 90162.41 (78715.73 to 104402.2) | 35316.45 (29996.81 to 41563.71) | 152111.88 (112618.33 to 174008.04) | 23685.88 (17247.3 to 28047.55) | -1.1 (-1.29 to -0.92) |
| Fiji | 9996.18 (8466.42 to 11658.15) | 28277.17 (22680.58 to 34470.24) | 19743.64 (15844.62 to 24265.63) | 24439.28 (18930.92 to 30721.41) | -0.72 (-0.85 to -0.59) |
| Marshall Islands | 472.82 (374.73 to 573.68) | 26372.68 (19608.12 to 33968.07) | 874.79 (675.9 to 1134.7) | 25741.9 (18929.26 to 33944.31) | 0.18 (0.02 to 0.34) |
| Republic of Moldova | 197890.44 (191624.44 to 202806.05) | 39827.64 (37637.68 to 41522.66) | 191090.14 (168409.4 to 214514.14) | 26140.01 (22640.96 to 29696.43) | -1.92 (-2.27 to -1.57) |
| Iraq | 273751.32 (233222.18 to 317130.59) | 32160.51 (26790.72 to 38240.51) | 608625.91 (492210.38 to 705910.43) | 26253.28 (20951.35 to 31037.61) | -1.02 (-1.12 to -0.91) |
| Micronesia (Federated States of) | 1330.89 (1002.2 to 1703.48) | 24922.91 (17866.67 to 33511.07) | 1935.15 (1397.51 to 2503.03) | 26497.6 (18688.03 to 35048.86) | 0.19 (0.14 to 0.25) |
| Sudan | 385003.38 (311147.61 to 455929.23) | 37000.78 (29022.17 to 44738.56) | 523031.54 (418555.21 to 651254.97) | 27666.86 (21230.97 to 35094.54) | -1.17 (-1.24 to -1.11) |
| Mongolia | 45836.93 (39043.43 to 52239.95) | 40108.32 (33147.57 to 47231.75) | 57953.59 (46952.26 to 72107.68) | 27897.45 (21819.99 to 35061.6) | -2.1 (-2.56 to -1.64) |
| Morocco | 484819.86 (429283.12 to 548865.09) | 32044.96 (27071.56 to 37160.22) | 963422.38 (769247.34 to 1113701.09) | 28400.5 (22301.38 to 33869.54) | -0.62 (-0.76 to -0.47) |
| Vanuatu | 1799.29 (1442.09 to 2301.93) | 25775.14 (19369.71 to 33979.49) | 5603.28 (4546.86 to 7199.78) | 29122.28 (22604.56 to 38215.88) | 0.3 (0.2 to 0.41) |
| Yemen | 189133.87 (152133.11 to 235139.59) | 36537.84 (28716.46 to 45954.66) | 409130.83 (337874.78 to 520195.85) | 29986.79 (23656.9 to 38344.44) | -0.86 (-0.96 to -0.76) |
| Kyrgyzstan | 85418.95 (80894.34 to 90222.03) | 24126.12 (22245.66 to 25870.62) | 139889.44 (124538.01 to 155865) | 30853.44 (27128.01 to 34597.3) | 1.18 (0.72 to 1.64) |
| Afghanistan | 341723.58 (279452.62 to 411782.34) | 41053.14 (31948.25 to 50687.31) | 335893.04 (266250.9 to 403589.95) | 31575.32 (24100.89 to 39258.56) | -1.1 (-1.24 to -0.97) |
| Nauru | 94.83 (78.24 to 115.62) | 30392.02 (23693.51 to 37951.31) | 104.4 (84.31 to 126.75) | 32486.28 (25248.94 to 40404.42) | 0.13 (-0.23 to 0.49) |
| Belarus | 503383.17 (480053.89 to 519798.81) | 31286.28 (29443.16 to 32766.01) | 679128.22 (553151.14 to 839150.37) | 33143.67 (26751.45 to 41061.37) | 0 (-0.42 to 0.42) |
| Oman | 32754.7 (26002.83 to 39928.53) | 50833.44 (40193.42 to 61902.61) | 44809.98 (40591.55 to 49778.29) | 33790.52 (29169.09 to 38524.51) | -1.32 (-1.46 to -1.17) |
| Syrian Arab Republic | 203785.7 (169200.07 to 241569.84) | 37227.56 (30504.51 to 44320.23) | 438409.75 (342885.09 to 560135.27) | 33802.51 (26527.01 to 42890.25) | -0.77 (-0.95 to -0.58) |
| Turkmenistan | 82325.32 (79039.75 to 84901.89) | 40394.13 (38001.73 to 42333.4) | 141957.32 (116886.56 to 171148.53) | 36555.01 (29785.45 to 44187.4) | -1.27 (-1.71 to -0.84) |
| Egypt | 1311695.32 (1205738.07 to 1447262.83) | 42448.49 (37352.3 to 48044.58) | 2502143.91 (1934326.49 to 3140631.15) | 37581.09 (28846.85 to 47668.05) | -0.3 (-0.4 to -0.21) |
| Solomon Islands | 5632.76 (4459.02 to 6963.16) | 38972.21 (29674.18 to 49847.78) | 12010.91 (9774.62 to 14182.85) | 40438.39 (31997.94 to 49554.79) | 0.07 (0.01 to 0.13) |
| Ukraine | 2964160.84 (2861645.61 to 3036758.52) | 32819.86 (31065.15 to 34202.03) | 4211566.36 (3690403.79 to 4821479.52) | 41752.47 (35969.24 to 48032.78) | 0.33 (-0.09 to 0.75) |
| Azerbaijan | 185330.46 (172888.19 to 196468.91) | 35064.61 (32046.79 to 37680.59) | 368599.63 (321922.85 to 420818.24) | 42052.03 (35869.76 to 48625.66) | 0.24 (0 to 0.48) |
| Tajikistan | 82604.18 (75262.17 to 89851.16) | 26351.71 (23481.33 to 29055.83) | 185251.84 (155196.2 to 222219.73) | 45403.8 (37896.78 to 54209.5) | 2.24 (2.03 to 2.44) |
| Uzbekistan | 398497.76 (377100.57 to 412338.73) | 31766.99 (29591.91 to 33273.1) | 1082040.91 (941241.67 to 1229116.08) | 68521.23 (60346.77 to 76953.89) | 2.83 (2.19 to 3.47) |

**Supplementary Table 7** Mortality of IHD among older adults in 1990 and 2019 and the EAPC in different countries and territories

| Death | 1990 |  | 2019 |  | 1990-2019 |
| --- | --- | --- | --- | --- | --- |
| Location | Death Cases NO.(95%UI) | ASMR/100,000 (95% CI) | Death Cases NO.(95%UI) | ASMR/100,000 (95% CI) | EAPC (95%CI) |
| Japan | 90943.89 (84039.86 to 95490.07) | 448.52 (411.47 to 472.42) | 86821.71 (72446.95 to 94673.73) | 159.71 (135.81 to 173.68) | -3.61 (-3.81 to -3.41) |
| Republic of Korea | 21540.59 (19792.5 to 22738.06) | 827.97 (726.18 to 924.68) | 19994.94 (17095.07 to 22793.07) | 183.98 (148.69 to 218.85) | -5.04 (-5.4 to -4.67) |
| France | 60288.72 (55630.26 to 63968.55) | 514.87 (466.26 to 556.41) | 39870.38 (34856.17 to 43814.33) | 198.57 (169.42 to 225.47) | -3.58 (-3.74 to -3.43) |
| Taiwan (Province of China) | 7988.39 (7575.7 to 8389.61) | 520.95 (474.96 to 563.65) | 11184.44 (9015.69 to 14009.46) | 229.94 (179.59 to 291.32) | -2.87 (-3.07 to -2.67) |
| Spain | 45405.17 (42018.52 to 47663.87) | 638.37 (574.95 to 691.39) | 33708.15 (29930.82 to 37365.43) | 237.85 (205.94 to 268.98) | -3.76 (-3.93 to -3.58) |
| Portugal | 13087.78 (12380.14 to 13604.3) | 746.92 (683.74 to 804.37) | 8829.83 (7842.97 to 9601.03) | 245.71 (213.25 to 275.85) | -4.38 (-4.59 to -4.17) |
| San Marino | 19.88 (17.01 to 23.09) | 465.85 (367.6 to 576.02) | 23.71 (16.08 to 32.81) | 257.7 (165.35 to 377.38) | -2.11 (-2.32 to -1.91) |
| Netherlands | 23394.02 (21990.91 to 24337.25) | 887.17 (818.59 to 942.18) | 12456.85 (11200.67 to 13402.94) | 268.3 (233.63 to 299.66) | -4.87 (-5.19 to -4.55) |
| Peru | 7787.54 (6691.95 to 8931.46) | 615.24 (500.28 to 735.22) | 10328.24 (7607.46 to 13344.02) | 269.15 (186.71 to 368.18) | -2.92 (-3.26 to -2.59) |
| Andorra | 27.51 (21.6 to 37.23) | 473.38 (345.69 to 659.15) | 46.34 (35.06 to 58.53) | 269.2 (189.24 to 359.7) | -2.11 (-2.34 to -1.89) |
| Israel | 6853.83 (6514.31 to 7083.03) | 1089.72 (1013.49 to 1149.67) | 4112.82 (3630.68 to 4412.64) | 270.04 (235.59 to 298.42) | -5.59 (-5.9 to -5.28) |
| Chile | 8892.87 (8437.34 to 9216.47) | 804.32 (741.29 to 855.94) | 8446.69 (7667.3 to 9038.76) | 283.28 (248.46 to 314.42) | -3.59 (-3.78 to -3.4) |
| Thailand | 16891.2 (14852.95 to 19096.3) | 514.7 (405.63 to 632.99) | 34171.14 (25492.88 to 44059.06) | 286.06 (199.69 to 383.7) | -2.53 (-2.71 to -2.36) |
| Australia | 26898.73 (25283.93 to 27770.43) | 1087.4 (1004.02 to 1143.2) | 16597.62 (14634.98 to 17922.97) | 293.7 (252.23 to 326) | -5.12 (-5.38 to -4.86) |
| Italy | 84370.75 (80023.53 to 90918.54) | 709.59 (669.03 to 765.61) | 64549.06 (55673.78 to 70209.93) | 294.49 (258.04 to 320.43) | -3.46 (-3.62 to -3.3) |
| Switzerland | 11881.77 (11114.08 to 12394.36) | 817.76 (752.1 to 868.85) | 7345.47 (6412.28 to 8229.39) | 295.96 (253.06 to 336.03) | -4.01 (-4.19 to -3.82) |
| Singapore | 2120.78 (2022.65 to 2187.4) | 927.36 (858.16 to 984.11) | 2708.59 (2399.8 to 2917.99) | 305.18 (262.75 to 339.12) | -4.19 (-4.4 to -3.98) |
| Luxembourg | 702.31 (662.17 to 732.63) | 969.46 (888.58 to 1039.67) | 411.69 (348.68 to 468.52) | 306.75 (256.33 to 357) | -4.24 (-4.36 to -4.12) |
| Denmark | 14141.24 (13424.75 to 14571.16) | 1258.94 (1180.9 to 1314.75) | 4785.13 (4299.58 to 5141.91) | 307.81 (267.46 to 344.87) | -5.7 (-6.01 to -5.4) |
| Norway | 10753.89 (10130.5 to 11074.88) | 1125.14 (1058 to 1164.24) | 3932.84 (3529.66 to 4245.1) | 307.98 (276.56 to 333.51) | -4.97 (-5.16 to -4.79) |
| Belgium | 18064.32 (16923.4 to 18857.35) | 862.48 (789.49 to 922.75) | 10187.41 (9176.72 to 11000.36) | 308.62 (269.54 to 342.49) | -3.89 (-4.04 to -3.73) |
| Panama | 1175.45 (1075.61 to 1240.75) | 705.44 (627.02 to 764.39) | 1640.9 (1276.15 to 2052.61) | 332.63 (251.68 to 420.69) | -2.37 (-2.61 to -2.11) |
| Slovenia | 2518.41 (1963.86 to 3276.82) | 818.97 (623.03 to 1098.29) | 2078.85 (1619.05 to 2670.13) | 337.7 (256.33 to 444.63) | -3.82 (-4.07 to -3.56) |
| Puerto Rico | 4229.83 (3977.15 to 4417.89) | 920.75 (849.33 to 979.42) | 3316.71 (2606.5 to 4099.76) | 339.8 (260.86 to 425.68) | -3.85 (-4.04 to -3.66) |
| Canada | 39853.72 (37414.66 to 41217.03) | 973.37 (896.33 to 1023.97) | 32577.58 (29304.75 to 34819.95) | 357.16 (310.95 to 393.41) | -4.1 (-4.35 to -3.84) |
| Iceland | 395.05 (367.19 to 414.72) | 1053.26 (963.64 to 1122.99) | 267.33 (233.27 to 295.09) | 361.83 (309.8 to 407.24) | -4.05 (-4.17 to -3.93) |
| Saint Lucia | 93.48 (87.13 to 99.29) | 908.98 (804 to 1007.63) | 93.14 (79.94 to 107.61) | 369.1 (300.81 to 440.75) | -3.51 (-4.05 to -2.96) |
| Monaco | 82.22 (66.25 to 96.56) | 773.02 (595.16 to 950.92) | 50.26 (39.44 to 58.66) | 371.52 (276.07 to 467.9) | -2.64 (-2.89 to -2.39) |
| Jamaica | 1137.63 (1040.24 to 1251.27) | 488.94 (428.56 to 548.5) | 1339.92 (1095.17 to 1599.84) | 375.81 (297.5 to 461.35) | -0.81 (-1.19 to -0.43) |
| United Kingdom | 151340.85 (143808.89 to 155119.68) | 1214.73 (1149.25 to 1249.24) | 65487.46 (60163.1 to 68555.31) | 378.08 (347.38 to 396.85) | -4.72 (-4.99 to -4.46) |
| Uruguay | 4656.5 (4398.25 to 4843.32) | 925.37 (849.6 to 987.26) | 2944.23 (2686.77 to 3141.87) | 393.95 (349.3 to 435.8) | -3.05 (-3.22 to -2.88) |
| Barbados | 312.15 (288.87 to 330) | 737.49 (662.89 to 801.09) | 257.41 (218.22 to 297.21) | 410.23 (337.39 to 486.96) | -2.53 (-2.82 to -2.23) |
| New Zealand | 5839.35 (5536.86 to 6038.71) | 1152.13 (1070.27 to 1213.7) | 4280.38 (3832.41 to 4573.26) | 412.71 (362.09 to 451.3) | -4.07 (-4.27 to -3.86) |
| Sweden | 25595.04 (23936.18 to 26469.01) | 1174.95 (1093.95 to 1229.63) | 12306.69 (11096.9 to 13480.99) | 414.73 (368.54 to 458.46) | -3.88 (-3.98 to -3.77) |
| Ecuador | 3423.84 (3178.53 to 3621.64) | 589 (523.74 to 648.5) | 7234.19 (5827.51 to 9050.69) | 418.68 (327.02 to 530.92) | -1.05 (-1.38 to -0.73) |
| Costa Rica | 1559.45 (1439.39 to 1646.72) | 791.2 (711.11 to 850.74) | 2516.68 (1986.56 to 3137.74) | 419.76 (324.28 to 528.86) | -2.54 (-2.81 to -2.28) |
| Ireland | 7633.8 (7280.14 to 7897.92) | 1425.38 (1343.16 to 1493.26) | 4112.54 (3648.87 to 4421.2) | 423.15 (367.19 to 465.3) | -4.86 (-5.13 to -4.59) |
| Brazil | 81263.14 (76935.95 to 84243.08) | 892.55 (828.04 to 936.99) | 118098 (108161.49 to 124504.39) | 429.25 (386.53 to 459.98) | -2.42 (-2.54 to -2.3) |
| Bermuda | 109.55 (103.07 to 115.14) | 1493.22 (1372.57 to 1605.02) | 77.9 (65.79 to 92.75) | 448.27 (369.21 to 538.54) | -4.31 (-4.68 to -3.94) |
| Colombia | 16232.13 (15216.05 to 17257.54) | 904.27 (827.11 to 972.47) | 28861.91 (22696.78 to 36062.87) | 449.07 (343.11 to 567.47) | -2.57 (-2.69 to -2.44) |
| Germany | 220434 (205965.06 to 229275.01) | 1262.76 (1165.65 to 1334.37) | 129449.96 (117763.11 to 138099.15) | 449.9 (401.14 to 492.48) | -3.94 (-4.23 to -3.65) |
| Austria | 16763.59 (15730.07 to 18317.38) | 1008.25 (927.21 to 1110.89) | 11663.38 (10469.88 to 12648.17) | 462.21 (408.45 to 509.77) | -3.22 (-3.42 to -3.02) |
| South Africa | 9583.57 (8178.81 to 10641.65) | 432.7 (363.47 to 492.66) | 21903.56 (19983.41 to 23540.75) | 468.26 (413.44 to 516.32) | 0.24 (-0.27 to 0.75) |
| Argentina | 38188.26 (36063.39 to 39576.57) | 1001.32 (916.53 to 1073.62) | 32572.94 (30030.9 to 34727.94) | 470.84 (415.95 to 523.53) | -2.57 (-2.8 to -2.34) |
| Greece | 16823.84 (15895.77 to 17508.06) | 846.08 (780.56 to 906.04) | 17756.45 (15941.78 to 19019.07) | 479.58 (426.12 to 525.44) | -2.37 (-2.54 to -2.21) |
| Belize | 93.13 (85.4 to 99.72) | 858.34 (764.12 to 947.21) | 137.64 (119.87 to 155.16) | 492.71 (413.74 to 573.66) | -2.46 (-2.93 to -1.98) |
| United States of America | 460010.59 (430685.02 to 474380.97) | 1077.41 (1006.86 to 1116.99) | 360456.19 (331381.09 to 379816.82) | 501.04 (458.05 to 530.22) | -3.11 (-3.32 to -2.9) |
| Kenya | 3248.77 (2711.71 to 3900.66) | 414.95 (341.62 to 504.1) | 9670.32 (7507.89 to 12076.78) | 507.8 (389.07 to 638.68) | 0.91 (0.74 to 1.09) |
| Cyprus | 1162.94 (1070.38 to 1298.13) | 1366.84 (1182.42 to 1597.07) | 1259.58 (1097.46 to 1513.62) | 508.24 (424.94 to 623.73) | -4.09 (-4.28 to -3.9) |
| Bahamas | 132.9 (122.24 to 143.14) | 806.75 (705.73 to 902.3) | 213.44 (176.42 to 258.01) | 522.84 (419.6 to 639.13) | -1.77 (-1.99 to -1.54) |
| Paraguay | 1545.7 (1353.86 to 1713.88) | 640.47 (537.08 to 734.99) | 3204.03 (2516.79 to 4063.66) | 523.69 (392.57 to 683.1) | -0.53 (-0.84 to -0.23) |
| South Sudan | 1311.92 (1022.51 to 1609.68) | 557.46 (390.2 to 746.56) | 1740.58 (1248.24 to 2309.94) | 525.77 (347.18 to 743.06) | -0.06 (-0.15 to 0.03) |
| Guatemala | 3128.04 (2826.19 to 3435.8) | 961.92 (847.97 to 1077.2) | 6660.87 (5374.66 to 8051.3) | 526.24 (417.79 to 639.88) | -2.42 (-2.86 to -1.97) |
| Ethiopia | 11844.78 (9196.32 to 15184.39) | 646.74 (476.41 to 856.7) | 21039.21 (15718.55 to 26305.03) | 529.13 (378.73 to 683.44) | -0.85 (-0.92 to -0.79) |
| Rwanda | 1864.89 (1474.24 to 2400.9) | 687.3 (480.63 to 954.64) | 2795.52 (2058.11 to 3654.7) | 529.71 (351.68 to 746.49) | -1.32 (-1.5 to -1.14) |
| Dominica | 90.02 (81.21 to 97.67) | 886.88 (754.88 to 1021.09) | 61.26 (51.71 to 72.72) | 530.7 (405.02 to 677.79) | -1.89 (-2.13 to -1.64) |
| Antigua and Barbuda | 57.13 (52.57 to 61.06) | 789.37 (689.78 to 888.4) | 57.96 (50.07 to 65.57) | 532.79 (437.29 to 632.1) | -1.56 (-1.77 to -1.35) |
| Malta | 713.9 (675.65 to 746.83) | 1357.74 (1256.77 to 1444.31) | 680.46 (599.23 to 754.49) | 533.65 (460.58 to 600.06) | -3.27 (-3.37 to -3.18) |
| Zambia | 1673.89 (1404.92 to 2008.57) | 635.72 (462.03 to 842.62) | 3140.68 (2536.26 to 3841.52) | 534.2 (385.74 to 712.55) | -0.81 (-1.07 to -0.54) |
| Uganda | 3220.29 (2386.18 to 4065.72) | 525.19 (352.41 to 716.93) | 6766.94 (4622.38 to 8485.5) | 538.72 (344.3 to 731.06) | -0.08 (-0.29 to 0.13) |
| Cook Islands | 10.19 (8.75 to 11.98) | 756.02 (574.94 to 966.1) | 16.67 (14.18 to 19.7) | 545.45 (418.9 to 688.85) | -1.22 (-1.39 to -1.05) |
| Malawi | 2136.42 (1777.84 to 2521.62) | 590.05 (439.19 to 765.26) | 3790.21 (3034.59 to 4661.57) | 545.7 (389.69 to 723.01) | -0.36 (-0.51 to -0.21) |
| Mexico | 29616.41 (27734.37 to 30749.48) | 659.91 (613.49 to 688.32) | 71733.89 (62218.03 to 81083.43) | 546.94 (465.14 to 624.59) | -0.67 (-0.87 to -0.47) |
| Greenland | 35.29 (32.05 to 38.81) | 1197 (1018.7 to 1389.84) | 40.24 (33.49 to 47.11) | 567.16 (446.14 to 694.72) | -3.2 (-3.47 to -2.92) |
| Finland | 13007.44 (12401.15 to 13422.38) | 1378.72 (1292.46 to 1445.01) | 9875.3 (8949.5 to 10643.62) | 569.3 (502.92 to 621.72) | -3.33 (-3.45 to -3.21) |
| Viet Nam | 27335.18 (22867.15 to 32540.26) | 624.07 (466.89 to 809.8) | 53733.35 (44122.58 to 63070.38) | 569.56 (423.52 to 728.64) | -0.42 (-0.54 to -0.29) |
| El Salvador | 2334.16 (2130.3 to 2484.44) | 712.55 (624.29 to 787.11) | 4342.46 (3388.91 to 5451.39) | 583.68 (438.15 to 745.38) | -0.76 (-0.9 to -0.62) |
| Mauritius | 1205.23 (1148.63 to 1254.47) | 1528.04 (1408.68 to 1636.72) | 1150.44 (956.46 to 1369.28) | 587.67 (471.47 to 717.14) | -4.19 (-4.63 to -3.76) |
| United Republic of Tanzania | 5614.39 (4590.77 to 6800.12) | 553.52 (405.46 to 720.01) | 13800 (10238.77 to 17320.85) | 592.64 (401.15 to 806.15) | 0.28 (0.23 to 0.33) |
| Myanmar | 18748.62 (15184.89 to 22815.8) | 817.9 (602.99 to 1077.3) | 28513.54 (25264.96 to 32712.7) | 612.19 (497.69 to 745.39) | -1.18 (-1.25 to -1.11) |
| Kuwait | 546.87 (499.54 to 590.14) | 1117.19 (985.85 to 1229.69) | 1297.21 (1087.43 to 1545.82) | 613.15 (497.22 to 736.98) | -2 (-2.32 to -1.69) |
| Suriname | 297.9 (275.47 to 316.08) | 1060.33 (938.04 to 1171.61) | 410.95 (347.28 to 479) | 615.18 (482.7 to 761.12) | -1.96 (-2.39 to -1.53) |
| Mozambique | 2759.24 (2250.69 to 3329.95) | 502.78 (362.77 to 675.62) | 6133.52 (4910 to 7763.59) | 616.21 (430.98 to 839.62) | 1.11 (0.93 to 1.29) |
| Equatorial Guinea | 159.68 (117.59 to 210.86) | 853.62 (571.8 to 1219.95) | 261.75 (189.82 to 349.17) | 617.86 (408.7 to 871.24) | -1.54 (-1.75 to -1.32) |
| Bangladesh | 30721.18 (25964.03 to 35216.29) | 633.7 (494.27 to 786.26) | 91759.17 (71857.75 to 112994.27) | 621.88 (457.48 to 801.59) | 0.42 (0.05 to 0.78) |
| Seychelles | 59.43 (54.44 to 65.12) | 847.67 (720.8 to 976.2) | 72.72 (64.13 to 81.26) | 629.78 (523.62 to 742.73) | -1.2 (-1.35 to -1.06) |
| Saint Kitts and Nevis | 74.23 (69.4 to 78.99) | 1471.16 (1318.69 to 1627.1) | 40.47 (36.36 to 45.32) | 635.89 (539.37 to 739.65) | -2.8 (-3.04 to -2.55) |
| Sri Lanka | 10776.15 (9818.56 to 11769.99) | 1012.56 (868.66 to 1164.88) | 18354.59 (13691.39 to 23372.79) | 643.72 (461.83 to 845.45) | -1.13 (-1.3 to -0.96) |
| Comoros | 160.73 (116.27 to 199.75) | 693.72 (465.67 to 935.16) | 330.41 (251.95 to 427.7) | 643.91 (445.88 to 879.96) | -0.29 (-0.36 to -0.22) |
| Bolivia (Plurinational State of) | 2770.19 (2042.89 to 3638) | 859.89 (602.09 to 1165.27) | 6013.22 (4265.31 to 7826.76) | 645.45 (439.64 to 890.89) | -1.04 (-1.29 to -0.78) |
| Grenada | 115.4 (106.45 to 123.56) | 1137.46 (1011.46 to 1257.17) | 78.34 (71.88 to 84.89) | 652.08 (569.7 to 733.53) | -2.01 (-2.26 to -1.77) |
| Cuba | 15931.22 (14881.79 to 16513.18) | 1229 (1140.32 to 1289.18) | 15650.48 (13076.99 to 18697.69) | 655.63 (536.67 to 793.6) | -2.59 (-2.89 to -2.29) |
| Nigeria | 34137.36 (25809.95 to 46266.39) | 797.79 (596.17 to 1084.73) | 50616.27 (37318.36 to 63761.46) | 658.81 (473.18 to 826.35) | -0.76 (-0.87 to -0.65) |
| Mauritania | 985.58 (831.24 to 1164.58) | 940.19 (716.57 to 1195.14) | 1414.26 (1150.6 to 1713.54) | 660.08 (486.26 to 852.34) | -1.15 (-1.31 to -0.99) |
| Eritrea | 401.99 (284.98 to 551.33) | 548.01 (344.07 to 838.54) | 1400.79 (1106.59 to 1757.78) | 672.57 (480.87 to 917.79) | 0.75 (0.69 to 0.82) |
| China | 439906.4 (391283.54 to 492134.36) | 561.92 (494.91 to 634.9) | 1470845.31 (1271726.35 to 1670664.02) | 673.56 (576.91 to 765.59) | 1.33 (1.03 to 1.63) |
| Burundi | 1892.75 (1466.75 to 2423.48) | 793.26 (559.28 to 1091.5) | 2693.09 (2014.03 to 3492.37) | 687.07 (474.68 to 944.65) | -0.59 (-0.65 to -0.53) |
| Angola | 2469.92 (1953.38 to 3022.85) | 752.31 (535.97 to 1010.38) | 6325.14 (4892.08 to 7847.34) | 701.2 (489.19 to 931.83) | -0.43 (-0.53 to -0.32) |
| Cambodia | 3034.1 (2504.23 to 3759.27) | 712.7 (533.23 to 940.93) | 8169.91 (6669.96 to 9696.9) | 701.97 (529.6 to 898.38) | -0.11 (-0.16 to -0.07) |
| Trinidad and Tobago | 1280.98 (1218.21 to 1344.55) | 1298.51 (1200.11 to 1390.53) | 1537.05 (1189.54 to 1944.48) | 702.49 (538.57 to 894.83) | -2.81 (-3.09 to -2.54) |
| Eswatini | 181.99 (150.32 to 213.6) | 675.71 (505.56 to 874.35) | 376.22 (288.71 to 492.68) | 704.02 (498.04 to 961.79) | 0.47 (0.24 to 0.7) |
| Benin | 1686.28 (1447.43 to 1952.96) | 788.06 (601.71 to 997.81) | 3254.85 (2656.16 to 3950.49) | 704.27 (526.86 to 906.98) | -0.31 (-0.37 to -0.25) |
| Namibia | 574.62 (485.48 to 673.28) | 752.16 (566.81 to 959.05) | 1018.8 (826.7 to 1209.49) | 705.5 (519.83 to 920.96) | -0.34 (-0.5 to -0.17) |
| Lesotho | 518.39 (400.14 to 633.37) | 511.93 (359.48 to 689.61) | 863.88 (650.99 to 1116.34) | 710.18 (484.9 to 978.63) | 1.66 (1.42 to 1.9) |
| Democratic People's Republic of Korea | 8610.66 (6734.7 to 10538.31) | 611.22 (429 to 812.85) | 26023.93 (21882.54 to 30713.47) | 711.58 (540 to 895.19) | 0.72 (0.49 to 0.96) |
| Democratic Republic of the Congo | 11003.07 (8832.74 to 14125.09) | 796.51 (583.28 to 1072.08) | 22076.91 (16101.45 to 29634.7) | 711.68 (481.84 to 999.28) | -0.44 (-0.5 to -0.38) |
| Djibouti | 61.16 (48.83 to 77.94) | 612.57 (443.15 to 826.6) | 333.65 (241.33 to 446.45) | 713.89 (481.17 to 992.74) | 0.61 (0.54 to 0.68) |
| Cameroon | 2682.47 (2136.76 to 3290.17) | 666.35 (483.99 to 881.86) | 7693.86 (6172.93 to 9744) | 715.29 (519.87 to 955.34) | 0.44 (0.26 to 0.62) |
| Liberia | 1001.12 (848.9 to 1189.43) | 851.18 (649.8 to 1082.92) | 1306.98 (1033.56 to 1655.54) | 717.39 (518.87 to 953.51) | -0.42 (-0.56 to -0.28) |
| Turkey | 49807.35 (43384.22 to 55453.27) | 1326.99 (1093.42 to 1559.29) | 72212.33 (58676.65 to 87983.4) | 718.82 (560.83 to 897.68) | -2.22 (-2.5 to -1.94) |
| Jordan | 1562.26 (1368.84 to 1772.73) | 1349.4 (1103.77 to 1607.55) | 4156.79 (3517.81 to 4944.67) | 722.58 (579.43 to 886.87) | -2.86 (-3.16 to -2.55) |
| Tonga | 42.63 (36.33 to 49.32) | 734.83 (570.8 to 917.02) | 66.84 (54.93 to 79.74) | 723.52 (552.62 to 913.75) | -0.06 (-0.26 to 0.15) |
| Gabon | 470.44 (385.55 to 566.9) | 810.81 (595.39 to 1063.21) | 717.04 (564.86 to 878.95) | 726.21 (515.96 to 966.59) | -0.36 (-0.45 to -0.28) |
| Senegal | 2595.95 (2154.54 to 2988.79) | 815.02 (612.12 to 1033.2) | 5368.15 (4334.58 to 6479.82) | 727.09 (535.47 to 945.9) | -0.31 (-0.39 to -0.22) |
| Mali | 3036.09 (2509.84 to 3611.46) | 805.7 (599.22 to 1034.48) | 5807.34 (4688.78 to 7135.88) | 728.11 (530.17 to 947.68) | -0.26 (-0.35 to -0.17) |
| Nepal | 5402.6 (4284.01 to 6869.16) | 611.62 (442.87 to 817.64) | 17153.32 (13676.73 to 20626.93) | 729.23 (538.2 to 937.45) | 0.71 (0.62 to 0.8) |
| Poland | 102114.39 (96833.97 to 104728.25) | 1913.11 (1798.19 to 1976.01) | 70123.49 (59575.78 to 81155.68) | 732.4 (609.54 to 857.99) | -3.72 (-3.92 to -3.52) |
| Niger | 1956.55 (1550.24 to 2397.42) | 824.92 (595.07 to 1095.21) | 5005.74 (3944.25 to 6299.52) | 738.87 (535.7 to 979.98) | -0.29 (-0.4 to -0.18) |
| Somalia | 1382.15 (1061.43 to 1725.02) | 695.53 (482.15 to 950.54) | 4090.63 (3015.94 to 5442.25) | 741.96 (509.48 to 1050.02) | 0.57 (0.43 to 0.7) |
| Chad | 2198.41 (1818.08 to 2647.37) | 723.46 (538.06 to 935.64) | 3955.51 (3270.18 to 4811.14) | 747.4 (556.22 to 975.43) | 0.22 (0.17 to 0.27) |
| Maldives | 122.65 (109.06 to 139.31) | 1601.03 (1333.05 to 1902.74) | 215.81 (180.39 to 251.96) | 747.87 (596.18 to 910.99) | -3.32 (-3.53 to -3.11) |
| Saint Vincent and the Grenadines | 101.88 (94.73 to 108.57) | 1144.66 (1023.5 to 1259.46) | 120.75 (107.74 to 135.18) | 760.67 (648.96 to 875.29) | -1.46 (-1.79 to -1.12) |
| Guinea | 2381.07 (1944.76 to 2923.19) | 681.47 (492.35 to 893.17) | 4267.1 (3501.6 to 5232.9) | 760.72 (563.93 to 1001.37) | 0.77 (0.63 to 0.91) |
| Venezuela (Bolivarian Republic of) | 10540.72 (9858.79 to 11045.05) | 1023.27 (932.3 to 1094.44) | 24682.95 (19447.01 to 31273.16) | 762.87 (587.02 to 968.33) | -1.35 (-1.53 to -1.18) |
| Madagascar | 3702.79 (3152.75 to 4338.18) | 754.53 (592.3 to 932.31) | 6617.08 (4817.84 to 8775.61) | 774.59 (525.24 to 1071.85) | 0.04 (-0.09 to 0.17) |
| Brunei Darussalam | 98.35 (88.47 to 109.48) | 1239.44 (1051.44 to 1442.6) | 169.54 (151.17 to 189.83) | 775.76 (656.19 to 902.77) | -1.42 (-1.56 to -1.28) |
| Botswana | 401.13 (320.13 to 497.11) | 731.59 (537.26 to 969.37) | 955.01 (727.58 to 1226.36) | 778.4 (548.77 to 1054.97) | 0.04 (-0.19 to 0.26) |
| Croatia | 12182.66 (11360.95 to 12842.07) | 1632.74 (1483.31 to 1757.1) | 10130.48 (8160.46 to 12248.23) | 785.97 (623.48 to 964) | -2.44 (-2.57 to -2.32) |
| Ghana | 4149.78 (3453.37 to 4876.77) | 749.17 (567.87 to 953.77) | 11652.42 (9844.15 to 13752.62) | 786.04 (603.67 to 999.61) | 0.41 (0.31 to 0.52) |
| Bhutan | 150.52 (112.41 to 196.86) | 688.12 (481.73 to 936.54) | 480.21 (376.87 to 581.67) | 802.17 (592.37 to 1028.96) | 0.6 (0.53 to 0.68) |
| Indonesia | 59562.48 (52093.41 to 67072.95) | 663.61 (566.44 to 761.48) | 162217.28 (138705.09 to 178906.17) | 802.38 (680.36 to 904.72) | 0.81 (0.71 to 0.91) |
| Estonia | 5645.85 (5375.67 to 5840.08) | 2183.68 (2047.55 to 2283.33) | 3191.14 (2525.95 to 4335.8) | 804.23 (613.49 to 1096.71) | -4.22 (-4.58 to -3.86) |
| Philippines | 14919.83 (13042.49 to 16744.18) | 556.61 (481.57 to 635.28) | 61589.56 (51338.64 to 71712.64) | 818.38 (679.54 to 957.41) | 1.91 (1.48 to 2.33) |
| Sierra Leone | 1891.43 (1505.46 to 2328.57) | 912.04 (662.45 to 1194.22) | 2863.45 (2199.92 to 3651.69) | 821.81 (583.66 to 1106.86) | -0.07 (-0.21 to 0.06) |
| Togo | 978.01 (827.97 to 1142.27) | 882.58 (679.56 to 1121.78) | 2572.47 (2106.62 to 3184.66) | 824.67 (612.14 to 1083.56) | -0.14 (-0.2 to -0.09) |
| American Samoa | 17.19 (15.42 to 19.19) | 828.48 (664.13 to 1012.23) | 41.04 (35.96 to 46.27) | 824.79 (662.26 to 991.22) | 0.08 (0.01 to 0.14) |
| Northern Mariana Islands | 7.62 (6.48 to 9) | 660.43 (496.1 to 845.16) | 38.67 (33.64 to 43.58) | 825.99 (666.55 to 997.87) | 1.22 (1.04 to 1.4) |
| Burkina Faso | 2826.43 (2322.35 to 3345.74) | 674.09 (496.63 to 865.81) | 7016.19 (5830.89 to 8208.04) | 829.47 (631.83 to 1043.52) | 1.08 (0.83 to 1.32) |
| India | 385184.73 (339316.1 to 431764.9) | 968.99 (841.4 to 1106.67) | 1034963.1 (894288.19 to 1186197.9) | 845.22 (718.66 to 971.77) | -0.54 (-0.65 to -0.43) |
| Czechia | 35365.75 (33669.51 to 36361.58) | 1981.13 (1862.18 to 2071.73) | 24068.12 (19965.31 to 28400.77) | 850.56 (692.79 to 1010.71) | -3.09 (-3.19 to -2.99) |
| Congo | 1036.97 (831.61 to 1292.33) | 1025.74 (742.89 to 1373.63) | 1929.82 (1460.5 to 2509.21) | 867.25 (601.82 to 1174.54) | -0.67 (-0.74 to -0.59) |
| Central African Republic | 919.49 (729.12 to 1144.02) | 905.78 (637.11 to 1231.26) | 1493.81 (1115.26 to 1969.75) | 873.12 (591.12 to 1223.84) | 0.01 (-0.06 to 0.09) |
| Malaysia | 9606.92 (8835.2 to 10324.58) | 1026.22 (861.07 to 1199.51) | 24733.29 (19744.61 to 30040.43) | 873.34 (672.87 to 1102.16) | -1.4 (-1.83 to -0.97) |
| Cabo Verde | 247.43 (216.05 to 273.13) | 787.75 (644.4 to 931.37) | 421.96 (359.59 to 472.76) | 875.05 (696.86 to 1059.67) | -0.32 (-0.74 to 0.1) |
| Albania | 2096.96 (1961.5 to 2219.54) | 1019.85 (914.49 to 1121.7) | 4813.68 (3773.87 to 6091.96) | 882.39 (671.13 to 1137.87) | -0.15 (-0.36 to 0.05) |
| Nicaragua | 991.54 (904.3 to 1069.83) | 644.94 (546.75 to 735.99) | 3936.06 (3394.66 to 4466.28) | 893.34 (731.77 to 1055.1) | 0.83 (0.44 to 1.23) |
| Sao Tome and Principe | 48.94 (41.83 to 56.07) | 727.79 (567.53 to 902.94) | 90.36 (74.18 to 104.87) | 923.07 (700.18 to 1148.66) | 0.88 (0.79 to 0.97) |
| Bahrain | 365.62 (324.36 to 409.08) | 2695.15 (2346.09 to 3050.45) | 560.44 (454.17 to 687.34) | 927.5 (737.76 to 1148.17) | -4.09 (-4.42 to -3.75) |
| Gambia | 267.62 (217.3 to 327.87) | 825.79 (608.61 to 1080.88) | 916.11 (754.62 to 1086.45) | 930.01 (700.15 to 1178.82) | 0.51 (0.4 to 0.63) |
| Honduras | 1507.13 (1241.55 to 2011.67) | 703.73 (538.21 to 953.88) | 5873.28 (4839.32 to 7272.15) | 930.3 (711.78 to 1213.05) | 1.13 (0.91 to 1.35) |
| Timor-Leste | 138.38 (113.42 to 171.13) | 649.92 (488.37 to 854.24) | 791.29 (610.89 to 965.64) | 934.95 (682.95 to 1196.12) | 1.43 (1.32 to 1.53) |
| Papua New Guinea | 1242.34 (867.53 to 1773.69) | 748.95 (480.77 to 1117.77) | 3741.01 (2723.64 to 4996.64) | 942.56 (649.19 to 1317.09) | 0.97 (0.84 to 1.11) |
| Lao People's Democratic Republic | 2007.7 (1609.28 to 2553.53) | 996.92 (735.46 to 1337.1) | 3820.41 (3113.95 to 4511.29) | 950 (731.19 to 1180.27) | -0.29 (-0.35 to -0.23) |
| Guam | 77.36 (68.08 to 87.45) | 1160.15 (952.22 to 1382.81) | 207.03 (175.45 to 242.88) | 950.16 (777.31 to 1135.21) | -0.76 (-1.11 to -0.4) |
| Bosnia and Herzegovina | 5599.5 (5240.23 to 5874.45) | 1432.42 (1290.14 to 1555.04) | 7168.22 (5887.25 to 8679.88) | 950.73 (751.99 to 1171.01) | -1.91 (-2.1 to -1.72) |
| Iran (Islamic Republic of) | 37398.22 (34519.3 to 40163.15) | 1699.75 (1526.44 to 1841.94) | 73566.5 (67595.57 to 79423.58) | 956.9 (867.78 to 1040.06) | -2.53 (-2.8 to -2.26) |
| Montenegro | 663.3 (598.97 to 729.16) | 938.56 (795.63 to 1086.76) | 1218.01 (1025.9 to 1412.31) | 977.09 (795.13 to 1181.53) | 0.4 (0.25 to 0.55) |
| Dominican Republic | 2950.68 (2599.31 to 3282.48) | 756.32 (640.56 to 868.37) | 10365.49 (8361.09 to 12925.54) | 979.42 (748.46 to 1259.78) | 1.76 (1.47 to 2.06) |
| Zimbabwe | 3154.01 (2789.71 to 3527.94) | 826.94 (660.56 to 1000.81) | 6366.65 (5177.96 to 7677.28) | 983.84 (741.25 to 1263.11) | 0.95 (0.79 to 1.12) |
| Guinea-Bissau | 393.45 (311.41 to 496.07) | 1013.14 (725.02 to 1360.52) | 624.26 (501.13 to 774.45) | 988.28 (737.64 to 1287.18) | 0.14 (0.06 to 0.23) |
| Libya | 2108.61 (1697.23 to 2604.21) | 1120.89 (859.8 to 1423.6) | 4977.68 (3966.51 to 6475.03) | 999.29 (744.74 to 1318.11) | -0.39 (-0.58 to -0.2) |
| Hungary | 30243.27 (28905.01 to 31197.45) | 1632.48 (1522.02 to 1726.7) | 26900.03 (22599.02 to 31524.36) | 1006.03 (828.72 to 1188.39) | -1.83 (-1.92 to -1.73) |
| Romania | 56493.91 (53844.23 to 59419.78) | 1798.9 (1662.76 to 1923.21) | 53639.27 (45381.18 to 62701.87) | 1013.84 (835.42 to 1209.82) | -2.56 (-2.79 to -2.34) |
| United States Virgin Islands | 104.29 (91.53 to 117.21) | 1206.6 (1014.63 to 1412.92) | 237.61 (212.44 to 262.75) | 1025.21 (856.17 to 1189.26) | -0.29 (-0.49 to -0.1) |
| United Arab Emirates | 405.18 (338.48 to 505.33) | 1829.28 (1447.24 to 2330.67) | 1593.41 (1185.65 to 2072.15) | 1038.23 (748.73 to 1373.02) | -2.19 (-2.79 to -1.58) |
| Pakistan | 50177.56 (41351.96 to 58734.72) | 818.05 (640.24 to 1002.45) | 106961.65 (89452.95 to 127930.52) | 1039.88 (840.5 to 1275.49) | 0.74 (0.51 to 0.97) |
| Tokelau | 1.89 (1.55 to 2.34) | 1073.63 (807.83 to 1403.14) | 1.51 (1.26 to 1.84) | 1042.2 (809.06 to 1319.59) | -0.06 (-0.17 to 0.05) |
| Guyana | 624.09 (559.26 to 687.14) | 1579.54 (1371.33 to 1788.84) | 698.34 (563.54 to 853.32) | 1092.92 (861.43 to 1349.86) | -1.23 (-1.42 to -1.04) |
| Latvia | 8900.37 (8485.19 to 9176.11) | 1945.11 (1830.09 to 2034.56) | 6796.36 (5826.2 to 7982.81) | 1156.19 (960.13 to 1383.8) | -2.1 (-2.35 to -1.85) |
| Serbia | 18973.63 (17370.48 to 20290.11) | 1515.23 (1319.02 to 1701.04) | 24361.26 (20225.22 to 28906.59) | 1159.08 (937.89 to 1404.57) | -1.28 (-1.53 to -1.03) |
| Niue | 3.48 (2.97 to 4.05) | 1197.54 (939.45 to 1479.88) | 3.1 (2.53 to 3.55) | 1159.66 (886.81 to 1413.71) | -0.21 (-0.3 to -0.12) |
| Tunisia | 7395.03 (6461.75 to 8453.76) | 1448.66 (1209.29 to 1705.51) | 16324.04 (12354.98 to 20824.82) | 1165.68 (858.93 to 1506.31) | -0.93 (-1.01 to -0.84) |
| Slovakia | 16331.35 (15393.32 to 16940.45) | 2196.75 (2020.68 to 2338.69) | 13554.72 (10965.72 to 16416.18) | 1166.21 (923.45 to 1425.33) | -2.3 (-2.5 to -2.09) |
| Haiti | 4619.6 (3825.97 to 5675.31) | 1456.42 (1109.96 to 1848.95) | 7781.6 (5677.01 to 10580.47) | 1168.88 (813.53 to 1605.72) | -0.65 (-0.76 to -0.54) |
| Palau | 13.39 (10.71 to 17.16) | 1280.12 (960.6 to 1678.06) | 23.28 (19.07 to 28.53) | 1171.85 (903.19 to 1484.43) | -0.3 (-0.35 to -0.25) |
| Saudi Arabia | 7796.47 (6384.25 to 9199.81) | 1457.71 (1149.7 to 1795.95) | 14779.35 (12453.23 to 17097.61) | 1187.97 (958.83 to 1413.63) | -0.76 (-0.98 to -0.54) |
| North Macedonia | 2718 (2533.73 to 2868.8) | 1384.99 (1232 to 1537.51) | 4292.29 (3514.02 to 5173.21) | 1195.72 (954.44 to 1474.62) | -0.86 (-1.13 to -0.59) |
| Samoa | 114.15 (97.35 to 133.93) | 1222.16 (949.88 to 1517.9) | 182.8 (155.31 to 217.53) | 1197.21 (956.56 to 1484.98) | -0.05 (-0.07 to -0.02) |
| Kiribati | 44.32 (36.69 to 52.22) | 1216.68 (907.03 to 1567.32) | 74.2 (58.76 to 91.21) | 1217.5 (883.56 to 1604.12) | -0.01 (-0.06 to 0.03) |
| Lithuania | 11979.76 (11262.17 to 12371.08) | 2069.71 (1935.96 to 2155.98) | 10695.25 (8936.49 to 12605.59) | 1237.08 (1021.2 to 1468.6) | -1.94 (-2.12 to -1.76) |
| Palestine | 1651.72 (1340.95 to 2006.47) | 1800.11 (1416.64 to 2226.05) | 2706.78 (2367.67 to 3092.74) | 1273.27 (1072.21 to 1501.71) | -1.56 (-1.7 to -1.42) |
| Georgia | 17514.33 (16322.4 to 18699.04) | 2494.88 (2280.69 to 2682.36) | 10255.1 (8766.04 to 11880.7) | 1282.85 (1058.46 to 1518.45) | -2.83 (-3.14 to -2.53) |
| Tuvalu | 9.67 (7.85 to 11.88) | 1344.43 (1006.87 to 1751.41) | 14.67 (11.92 to 18.69) | 1329.69 (1016.59 to 1735.56) | -0.04 (-0.08 to 0.01) |
| Bulgaria | 30715.78 (28586.13 to 32089.62) | 2273.99 (2086.37 to 2423.3) | 27506.79 (22895.13 to 32410.14) | 1331.23 (1077.31 to 1603.03) | -2.78 (-3.17 to -2.38) |
| Algeria | 23689.69 (19832.12 to 27843.77) | 2309.94 (1910.69 to 2734.87) | 45331.63 (37259.3 to 54827.54) | 1340.35 (1064.89 to 1643.4) | -1.98 (-2.09 to -1.88) |
| Fiji | 492.14 (420.82 to 573.51) | 1554.97 (1243.05 to 1899.96) | 984.66 (800.35 to 1204.21) | 1380.51 (1069.19 to 1730.49) | -0.65 (-0.79 to -0.5) |
| Armenia | 5360.62 (5080.92 to 5596.47) | 2001.63 (1869.2 to 2115.86) | 7115.81 (6054.15 to 8213.09) | 1381.46 (1159.77 to 1605.59) | -2.08 (-2.3 to -1.85) |
| Russian Federation | 389819.63 (375198.08 to 397909.96) | 1868.1 (1776.44 to 1917.48) | 423119.55 (369503.06 to 474629.97) | 1403.17 (1210.01 to 1585.77) | -1.32 (-1.75 to -0.89) |
| Lebanon | 4920.95 (4315.82 to 5664.38) | 2102.87 (1778.97 to 2468.66) | 9276.54 (6726.18 to 10641.76) | 1428.04 (1016.44 to 1689.76) | -1.08 (-1.24 to -0.92) |
| Marshall Islands | 23.32 (18.49 to 28.34) | 1433.19 (1058.38 to 1866.79) | 41.22 (31.97 to 52.94) | 1450.38 (1066.99 to 1908.28) | 0.28 (0.13 to 0.42) |
| Kazakhstan | 23867.37 (22610.62 to 24841.96) | 1789.44 (1651.28 to 1898) | 25137.96 (21975.12 to 28313.83) | 1486.53 (1275.06 to 1702.93) | -1.31 (-1.98 to -0.63) |
| Micronesia (Federated States of) | 64.09 (48.39 to 81.96) | 1360.37 (969.99 to 1834.06) | 92.76 (69.07 to 118.42) | 1491.31 (1063.04 to 1963.98) | 0.3 (0.26 to 0.34) |
| Iraq | 14903.3 (12658.2 to 17205.1) | 1827.97 (1514.03 to 2174.04) | 32739.81 (27229.05 to 37714.16) | 1538.54 (1239.12 to 1808.14) | -0.89 (-0.98 to -0.79) |
| Qatar | 157.35 (128.31 to 186.58) | 2618.5 (2037.9 to 3138.18) | 494.54 (383.38 to 622.04) | 1596.2 (1255.95 to 1990.51) | -1.88 (-2.19 to -1.56) |
| Vanuatu | 89.57 (71.85 to 114.62) | 1424.96 (1064.71 to 1880.69) | 285.15 (230.91 to 363.74) | 1625.6 (1252.42 to 2127.58) | 0.36 (0.27 to 0.44) |
| Sudan | 20335.86 (16393.74 to 23930.93) | 2102.49 (1644.16 to 2530) | 28990.54 (23126.04 to 35722.47) | 1628.29 (1249.07 to 2046.95) | -1.06 (-1.13 to -0.98) |
| Republic of Moldova | 11739.56 (11270.12 to 12045.98) | 2569.22 (2410.43 to 2681.61) | 11875.91 (10470.51 to 13337.74) | 1647.09 (1420.08 to 1868.75) | -2.03 (-2.37 to -1.69) |
| Morocco | 25587.38 (22485.04 to 28904.96) | 1848.9 (1543.32 to 2150.58) | 52825.21 (42762.74 to 60660.23) | 1688.7 (1327.45 to 2012.22) | -0.48 (-0.67 to -0.3) |
| Mongolia | 2573.39 (2210.17 to 2913.02) | 2398.37 (1995.46 to 2800.14) | 3276.76 (2693.38 to 4038.52) | 1728.29 (1369.2 to 2148.65) | -2 (-2.48 to -1.52) |
| Yemen | 9427.03 (7671.37 to 11609.29) | 2050.9 (1619.68 to 2557.85) | 21670.58 (18092.97 to 27264.89) | 1743.57 (1380.94 to 2213.32) | -0.71 (-0.8 to -0.62) |
| Afghanistan | 17190.6 (14203.95 to 20561.42) | 2265.71 (1764.97 to 2778.77) | 18260.08 (14638.07 to 21849.74) | 1792.35 (1359.47 to 2227.69) | -0.99 (-1.11 to -0.87) |
| Nauru | 4.59 (3.82 to 5.52) | 1680.87 (1311.75 to 2087.98) | 4.67 (3.79 to 5.6) | 1825.67 (1417.14 to 2264.44) | 0.25 (-0.07 to 0.57) |
| Kyrgyzstan | 4970.63 (4639.09 to 5257.84) | 1420.31 (1295.08 to 1527.84) | 8560.96 (7611.06 to 9496.74) | 1981.58 (1736.98 to 2217.4) | 1.59 (1.09 to 2.1) |
| Belarus | 31368.23 (29633.75 to 32561.35) | 1985.75 (1848.66 to 2085.77) | 42314.09 (34671.58 to 51924.17) | 2050.02 (1661.84 to 2518.66) | -0.08 (-0.44 to 0.28) |
| Syrian Arab Republic | 11020.33 (9234.65 to 12994.43) | 2197.57 (1808.49 to 2598.37) | 24471.09 (19324.71 to 30922.94) | 2102.95 (1670.4 to 2626.34) | -0.54 (-0.71 to -0.37) |
| Oman | 1637.38 (1312.94 to 1972.68) | 2888.88 (2297.54 to 3495.74) | 2389.08 (2165.98 to 2644.36) | 2111.65 (1813.19 to 2409.2) | -0.98 (-1.16 to -0.79) |
| Solomon Islands | 252.68 (202.45 to 310.06) | 2029.87 (1541.73 to 2593.29) | 566.38 (465.76 to 664.74) | 2159.11 (1708.63 to 2640.92) | 0.16 (0.11 to 0.2) |
| Egypt | 69118.71 (63566.07 to 76080.71) | 2456.19 (2152.42 to 2779.01) | 129712.1 (101485.25 to 162968.64) | 2191.18 (1691.94 to 2754.19) | -0.25 (-0.35 to -0.14) |
| Turkmenistan | 4605.87 (4380 to 4751.03) | 2441.25 (2272.22 to 2561.73) | 7951.5 (6604.69 to 9564.17) | 2200.98 (1797.72 to 2647.17) | -1.28 (-1.7 to -0.87) |
| Ukraine | 181154.38 (173720.21 to 185842.66) | 2085.83 (1959.95 to 2176.19) | 261052.33 (228152.12 to 297589.4) | 2579.56 (2219.23 to 2956.42) | 0.24 (-0.13 to 0.62) |
| Azerbaijan | 10347.39 (9560.56 to 11013.48) | 2034.26 (1844.2 to 2191.08) | 21538.9 (18963.46 to 24295.2) | 2761.1 (2364.33 to 3172.46) | 0.78 (0.56 to 1) |
| Tajikistan | 4653.76 (4170.31 to 5110.67) | 1495.74 (1309.86 to 1661.82) | 10080.62 (8567.01 to 11975.18) | 2875.52 (2418.26 to 3402.71) | 2.67 (2.43 to 2.9) |
| Uzbekistan | 24060.22 (22437.06 to 25033.1) | 1959.21 (1805.31 to 2060.05) | 57174.95 (50364.91 to 64255.63) | 4527.55 (4029.27 to 5028.11) | 3.18 (2.54 to 3.83) |

**Supplementary Table 8** Incidence of IHD among older adults in 1990 and 2019 and the EAPC in different countries and territories.

| Location | 1990 |  | 2019 |  | 1990-2019 |
| --- | --- | --- | --- | --- | --- |
|  | Incidence cases NO.(95%UI) | ASIR/100,000 (95% CI) | Incidence cases NO.(95%UI) | ASIR/100,000 (95% CI) | EAPC (95%CI) |
| Uzbekistan | 40530.52 (36240.34 to 45051.82) | 3230.36 (2677.43 to 3841.55) | 93238.68 (85462.06 to 101580.52) | 6431.94 (5753.17 to 7146.19) | 2.17 (1.78 to 2.57) |
| Iran (Islamic Republic of) | 133815.04 (110295.55 to 159846.92) | 5099.51 (3848.49 to 6537.13) | 371117.93 (313119.61 to 436022.12) | 4605.54 (3471.4 to 5890.86) | -0.57 (-0.66 to -0.47) |
| Azerbaijan | 18811.01 (16505.03 to 21401.45) | 3594.43 (2867.78 to 4405.88) | 37777.86 (33764.01 to 42206.1) | 4410.79 (3722.71 to 5191.48) | 0.49 (0.38 to 0.6) |
| Tajikistan | 10452.2 (9000.01 to 12068.63) | 3304.96 (2531.49 to 4161.56) | 17530.52 (15406.81 to 19742.97) | 4407.49 (3605.35 to 5275.01) | 1.11 (1.06 to 1.16) |
| Egypt | 135457.69 (122854 to 148947.56) | 4506.1 (3892.06 to 5193.31) | 278600.14 (253824.18 to 305297.69) | 4289.23 (3715.63 to 4926.72) | -0.12 (-0.19 to -0.05) |
| Oman | 2355.54 (1985.53 to 2785.81) | 3840.4 (2909.63 to 4947) | 5786 (4864.92 to 6808.58) | 4248.01 (3235.7 to 5423.98) | 0.23 (0.14 to 0.32) |
| Syrian Arab Republic | 21054.71 (18322.9 to 24089.39) | 3952.04 (3162.9 to 4862.47) | 53069.5 (46525.39 to 60193.55) | 4140.27 (3402.81 to 4968.23) | -0.08 (-0.19 to 0.03) |
| Ukraine | 301847.58 (258377.26 to 349602.24) | 3405.66 (2627.87 to 4322.41) | 387221.52 (330788.27 to 447778.2) | 3833.39 (2963.72 to 4856.89) | 0.13 (-0.16 to 0.42) |
| Kuwait | 1881.78 (1553.86 to 2221.4) | 3612.31 (2655.75 to 4754.5) | 8193.87 (6755.39 to 9715.22) | 3731.75 (2736.16 to 4850.63) | 0.07 (0 to 0.14) |
| United Arab Emirates | 900.74 (736.82 to 1085.52) | 3419.71 (2526.55 to 4466.84) | 7467.65 (6099.33 to 9045.1) | 3705.52 (2729.28 to 4840.91) | 0.24 (0.21 to 0.27) |
| Bahrain | 602.87 (499.53 to 716.27) | 3886.04 (2871.34 to 5092.62) | 3116.42 (2559.93 to 3769.93) | 3689.14 (2713.38 to 4841.61) | -0.26 (-0.31 to -0.21) |
| Afghanistan | 32710.57 (28977.83 to 37095.91) | 4101.77 (3329.49 to 4972.78) | 38731.17 (34115.08 to 44368.1) | 3682.11 (2956.93 to 4499.69) | -0.56 (-0.63 to -0.48) |
| Morocco | 57699.27 (51166.26 to 65120.54) | 3891.94 (3187.09 to 4699.48) | 123674.53 (110475.72 to 137312.03) | 3672.68 (3080.76 to 4335.03) | -0.31 (-0.37 to -0.26) |
| Turkmenistan | 7431.59 (6381.15 to 8472.27) | 3750.66 (2910.82 to 4692.02) | 13981.5 (12157.15 to 15980.59) | 3658.32 (2876.04 to 4526.87) | -0.43 (-0.58 to -0.28) |
| Mongolia | 4336.96 (3679.68 to 5013.77) | 3868.3 (2961.28 to 4911.44) | 7504.93 (6393.54 to 8780.1) | 3633.95 (2762.22 to 4675.42) | -0.47 (-0.62 to -0.32) |
| Yemen | 18612.51 (15983.53 to 21549.69) | 3771.59 (2984.34 to 4685.85) | 48277.38 (42246.86 to 55549.27) | 3616.68 (2902.82 to 4424.42) | -0.27 (-0.32 to -0.23) |
| Iraq | 33765.28 (29701.3 to 38449.97) | 4022.87 (3234.35 to 4943.4) | 82798.85 (72770.29 to 93438.6) | 3603.2 (2930.33 to 4361.14) | -0.46 (-0.49 to -0.42) |
| Sudan | 40329.1 (35637.39 to 45527.14) | 3968.47 (3235.1 to 4803.33) | 67344.84 (59516.77 to 75804.02) | 3584.56 (2889.7 to 4356.72) | -0.49 (-0.53 to -0.45) |
| Palestine | 3430.65 (2859.04 to 4058.71) | 3584.64 (2681.22 to 4644.87) | 8245.42 (6870.07 to 9686.83) | 3580.04 (2690.39 to 4619.35) | -0.07 (-0.1 to -0.04) |
| Lebanon | 9450.2 (8148.06 to 10919.94) | 3767.19 (2926.54 to 4729.62) | 23021.06 (19862.29 to 26283.21) | 3579.08 (2788.02 to 4451.19) | -0.15 (-0.22 to -0.07) |
| Kyrgyzstan | 11255.94 (9570.09 to 13028.91) | 3163.08 (2425.94 to 4038.02) | 15803.78 (13757.44 to 17941.74) | 3477.8 (2770.87 to 4273.52) | 0.46 (0.35 to 0.58) |
| Jordan | 4597.15 (3809.28 to 5445.54) | 3673.24 (2733.51 to 4762.04) | 21826.35 (18187.66 to 25665.11) | 3415.81 (2534.47 to 4443.31) | -0.36 (-0.44 to -0.28) |
| Qatar | 242.71 (199.5 to 290.34) | 3365.42 (2463.61 to 4383.06) | 2211.19 (1777.49 to 2667.35) | 3407.58 (2501.37 to 4496.81) | -0.08 (-0.12 to -0.05) |
| Saudi Arabia | 18234.61 (15533.56 to 21107.22) | 3220.42 (2488.79 to 4037.11) | 47483.47 (40635.68 to 55000.64) | 3394.23 (2655.09 to 4239.31) | 0.17 (0.07 to 0.28) |
| Estonia | 9851.26 (8657.28 to 11191.42) | 3796.8 (3029.63 to 4653.15) | 12302.7 (10260.92 to 14384.38) | 3394.17 (2553.9 to 4390.63) | -0.48 (-0.57 to -0.38) |
| Libya | 6160.8 (5091.99 to 7239.93) | 3155.94 (2347.01 to 4102.21) | 17199.91 (14438.88 to 20190.38) | 3388.56 (2556.73 to 4350.88) | 0.15 (0.08 to 0.22) |
| Armenia | 10504.75 (9064.89 to 11996.61) | 3659.58 (2834.57 to 4544.14) | 17130.35 (14778.8 to 19666.64) | 3257.41 (2524.85 to 4071.34) | -0.58 (-0.66 to -0.5) |
| Algeria | 52245.03 (46019.97 to 59121.79) | 4158.53 (3440.37 to 4983.86) | 119143.8 (105060.63 to 134068.43) | 3244.4 (2655.22 to 3898.23) | -1.05 (-1.12 to -0.98) |
| Belarus | 49896.89 (45075.06 to 55436.45) | 3123.99 (2630.71 to 3677.01) | 65984.38 (60362.04 to 72443.92) | 3205.88 (2748.06 to 3713.74) | -0.04 (-0.23 to 0.15) |
| Tunisia | 18346.38 (15649.35 to 21085.63) | 3271.61 (2544.64 to 4092.25) | 46977.59 (40600.49 to 53800.48) | 3189.71 (2524.85 to 3958.55) | -0.23 (-0.28 to -0.18) |
| Kazakhstan | 46326.01 (41167 to 51966.18) | 3316.8 (2721.81 to 3972.5) | 54036.57 (47390.41 to 61304.33) | 2924.4 (2373.62 to 3556.2) | -0.73 (-1.08 to -0.37) |
| Pakistan | 163286.16 (139388.32 to 190038.18) | 2561.95 (1985.92 to 3231.86) | 327177.97 (277985.34 to 382998.37) | 2915.44 (2260.96 to 3657.77) | 0.42 (0.31 to 0.52) |
| Republic of Moldova | 16850.59 (14915.89 to 18932.36) | 3488.59 (2860.42 to 4187.75) | 20654.72 (18185.44 to 23294.06) | 2835.41 (2266.56 to 3450.32) | -0.89 (-1.06 to -0.73) |
| Georgia | 29573.69 (26281.8 to 32968.42) | 4051.16 (3352.5 to 4813.29) | 21956.49 (19292.77 to 24701.3) | 2814.66 (2261.04 to 3424.97) | -1.58 (-1.73 to -1.44) |
| Russian Federation | 648777.23 (556379.96 to 746819.85) | 3017.02 (2328.19 to 3813.15) | 814305.71 (696166.64 to 943349.99) | 2679.02 (2061.09 to 3398.11) | -0.62 (-0.82 to -0.42) |
| India | 1154512.43 (978459.29 to 1353532.02) | 2549.8 (1964.71 to 3210.8) | 3266096.77 (2789666.66 to 3792583.92) | 2478.7 (1920.43 to 3116.35) | -0.16 (-0.28 to -0.04) |
| Latvia | 13122.46 (11522.83 to 14805.01) | 2876.44 (2304.27 to 3494.38) | 13639.53 (11744.91 to 15654.84) | 2460 (1901.97 to 3100.03) | -0.88 (-0.98 to -0.78) |
| Lithuania | 17612.88 (15612.72 to 19701.13) | 3067.58 (2491.53 to 3690.08) | 19395.26 (17024.79 to 21805.98) | 2416.95 (1898.83 to 2981.21) | -0.89 (-0.97 to -0.81) |
| Bhutan | 568.11 (463.07 to 682.01) | 2334.49 (1715.02 to 3019.04) | 1519.32 (1243.43 to 1802.03) | 2369.2 (1753.9 to 3075.58) | 0.07 (0.03 to 0.1) |
| Trinidad and Tobago | 2479.77 (2045.1 to 2946.54) | 2429.27 (1815.76 to 3161.2) | 5365.63 (4425.03 to 6346.55) | 2320.18 (1718.43 to 3028.23) | -0.22 (-0.27 to -0.18) |
| Suriname | 604.42 (500.01 to 720.63) | 2079.75 (1525.9 to 2714.92) | 1546.45 (1269.33 to 1835.7) | 2246.09 (1644.14 to 2945.13) | 0.26 (0.21 to 0.31) |
| Belize | 230.96 (189.26 to 273.9) | 2100.75 (1536.27 to 2765.43) | 654.24 (539.05 to 779.35) | 2223.91 (1629.51 to 2902.3) | 0.19 (0.15 to 0.23) |
| Guyana | 952.57 (782.43 to 1129.78) | 2285.37 (1701.32 to 2968.13) | 1530.41 (1257.3 to 1811.97) | 2223.45 (1644.83 to 2905.78) | -0.1 (-0.13 to -0.07) |
| Serbia | 32553.01 (28643.89 to 36979.51) | 2421.64 (1962.36 to 2937.71) | 47495.52 (41895.1 to 53550.11) | 2206.1 (1791.53 to 2679.44) | -0.67 (-0.78 to -0.55) |
| Nepal | 21668.48 (17895.4 to 25767.23) | 2258.94 (1695.48 to 2929.93) | 56010.63 (47699.92 to 64875.64) | 2182.36 (1703.13 to 2732.84) | -0.07 (-0.1 to -0.03) |
| Grenada | 199.61 (165.29 to 235.45) | 2086.45 (1534.29 to 2712.59) | 279.69 (227.97 to 333.69) | 2170.19 (1600.61 to 2829.85) | 0.11 (0.09 to 0.14) |
| Bangladesh | 109637.55 (93868.08 to 126763.47) | 2174.25 (1710.88 to 2683.17) | 329982.02 (289757.59 to 374544.74) | 2145.84 (1738.97 to 2603.26) | 0.12 (0.04 to 0.2) |
| Saint Lucia | 237.29 (193.9 to 284.9) | 2131.54 (1562.84 to 2798.33) | 547.07 (451.92 to 651.18) | 2142.38 (1576.2 to 2817.58) | -0.01 (-0.04 to 0.01) |
| Jamaica | 4376.69 (3570.89 to 5181.59) | 1917.48 (1409.34 to 2496.96) | 7475.11 (6163.36 to 8873.67) | 2114.55 (1559.2 to 2759.31) | 0.39 (0.34 to 0.44) |
| Saint Vincent and the Grenadines | 189.31 (154.77 to 227.95) | 2060.21 (1511.65 to 2697.78) | 349 (286.27 to 415.63) | 2108.71 (1533.85 to 2765.6) | 0.08 (0.05 to 0.12) |
| Saint Kitts and Nevis | 107.64 (87.61 to 128.9) | 2041.52 (1504.26 to 2677.15) | 154.41 (127.81 to 185.53) | 2078.51 (1530.14 to 2727.9) | 0.06 (0.04 to 0.08) |
| Barbados | 803.35 (661.39 to 950.96) | 2026.78 (1484.83 to 2668.96) | 1315.71 (1073.62 to 1563.77) | 2071.95 (1533.07 to 2711.78) | 0.06 (0.03 to 0.09) |
| Dominica | 196.63 (161.73 to 234.02) | 2013.86 (1473.99 to 2624.43) | 233.05 (194.85 to 274.99) | 2065.44 (1523.03 to 2715.91) | 0.13 (0.09 to 0.16) |
| Bahamas | 343.81 (281.75 to 408.8) | 2020.88 (1479.58 to 2645.71) | 891.01 (732.04 to 1058.69) | 2050.53 (1505.93 to 2680.71) | 0.07 (0.04 to 0.09) |
| Haiti | 7477.83 (6279.53 to 8791.16) | 2153.54 (1645.26 to 2733.41) | 14791.1 (12532.68 to 17387.44) | 2037.67 (1557.52 to 2589.9) | -0.24 (-0.29 to -0.19) |
| Antigua and Barbuda | 138.59 (113.15 to 164.3) | 2000.96 (1467.22 to 2613.02) | 231.81 (189.49 to 276.65) | 1962.03 (1431.55 to 2571.72) | -0.12 (-0.14 to -0.09) |
| Dominican Republic | 7666.48 (6392.6 to 9084.79) | 1840.44 (1365.54 to 2389.16) | 20964.97 (18055.31 to 24175.96) | 1947.7 (1505.76 to 2457.43) | 0.25 (0.2 to 0.31) |
| United States Virgin Islands | 181.78 (148.38 to 215.78) | 1930.09 (1409.69 to 2530.69) | 487.77 (396.56 to 583.06) | 1945.04 (1426.44 to 2560.88) | 0.04 (0.02 to 0.06) |
| Puerto Rico | 9298.61 (7856.69 to 10900.81) | 2016.15 (1511.51 to 2581.31) | 18027.92 (14870.44 to 21568.67) | 1941.6 (1440.1 to 2534.62) | -0.16 (-0.23 to -0.08) |
| Czechia | 53846.64 (49335.53 to 59211.24) | 3002.96 (2559.49 to 3516.08) | 52631.78 (46113.08 to 59900.84) | 1887.58 (1496.57 to 2308) | -1.98 (-2.14 to -1.82) |
| Bulgaria | 40032.48 (36081.85 to 44233.38) | 2770.24 (2361.46 to 3226.74) | 37610.22 (33610.51 to 41775.85) | 1856.99 (1523.89 to 2220.38) | -2.07 (-2.4 to -1.74) |
| Australia | 66897.03 (59003.74 to 75512.4) | 2652.49 (2138.41 to 3231.11) | 97629.54 (81752.53 to 114010.56) | 1828.51 (1378.75 to 2355.45) | -1.44 (-1.54 to -1.33) |
| Turkey | 103771.03 (93121.6 to 115169.08) | 2668.64 (2227.11 to 3179.27) | 187874.02 (166491.73 to 211606.7) | 1820.7 (1490.37 to 2195.46) | -1.69 (-1.82 to -1.56) |
| Bermuda | 143.85 (118.42 to 171.14) | 1893.33 (1388.85 to 2456.64) | 298.81 (244.85 to 356.82) | 1781.33 (1307.92 to 2339.26) | -0.29 (-0.31 to -0.26) |
| Hungary | 45960.01 (41496.11 to 51304.21) | 2440.78 (2023.53 to 2891.94) | 46404.62 (41006.6 to 52263.51) | 1767.98 (1432.44 to 2144.62) | -1.29 (-1.34 to -1.24) |
| Montenegro | 1243.01 (1030.87 to 1459.95) | 1704.93 (1261.56 to 2231.48) | 2137.89 (1784.03 to 2520.36) | 1668.81 (1235.7 to 2165.8) | -0.11 (-0.14 to -0.09) |
| Cuba | 27210.67 (23877.13 to 30892.82) | 2118.21 (1677.67 to 2596.67) | 38182.19 (32821.43 to 43782.88) | 1650.57 (1269.49 to 2072.92) | -0.97 (-1.07 to -0.88) |
| Slovakia | 19311.9 (17320.82 to 21629.27) | 2571.42 (2134.31 to 3067.52) | 19561.28 (17013.09 to 22202.38) | 1647.31 (1301.71 to 2033.26) | -1.73 (-1.88 to -1.58) |
| Bosnia and Herzegovina | 7792.43 (6633.01 to 9021.29) | 1836.04 (1410.65 to 2313.03) | 12792.88 (10920.23 to 14797.9) | 1646.77 (1260.65 to 2090.94) | -0.54 (-0.62 to -0.46) |
| Romania | 79521.89 (72693.34 to 87151.61) | 2445.77 (2109.42 to 2817.36) | 82782.65 (74550.69 to 91382.81) | 1607.38 (1342.77 to 1899.44) | -1.92 (-2.07 to -1.77) |
| North Macedonia | 3490.53 (2933.29 to 4098.37) | 1676.88 (1263.71 to 2157.8) | 6346.55 (5346.76 to 7410.81) | 1605.59 (1214.92 to 2048.25) | -0.31 (-0.42 to -0.2) |
| Gambia | 479.94 (390.99 to 570.43) | 1350.97 (985.81 to 1789.68) | 1466.27 (1208.61 to 1732.82) | 1435.48 (1052.05 to 1871.2) | 0.24 (0.22 to 0.26) |
| New Zealand | 13073.48 (10811.11 to 15493.95) | 2558.49 (1899.07 to 3312.16) | 14430.11 (12773.56 to 16097.56) | 1430.1 (1168.15 to 1717.02) | -1.98 (-2.02 to -1.93) |
| Sierra Leone | 3120.17 (2580.31 to 3715.14) | 1440.51 (1064.16 to 1887.92) | 5212.49 (4340.99 to 6199.1) | 1423.53 (1057.9 to 1848.2) | -0.04 (-0.06 to -0.03) |
| Slovenia | 4987.9 (4132.51 to 5863.91) | 1624.93 (1215.48 to 2095.86) | 8097.82 (6741.25 to 9537.91) | 1421.84 (1051.34 to 1863.1) | -1.12 (-1.36 to -0.89) |
| Zimbabwe | 5473 (4521.29 to 6475.4) | 1299.95 (968.51 to 1691.94) | 9897.13 (8337.19 to 11661.01) | 1419.41 (1069.1 to 1821.87) | 0.32 (0.23 to 0.41) |
| Albania | 3269.44 (2719.29 to 3872.58) | 1470.99 (1093.16 to 1910.54) | 7816.83 (6595.95 to 9113.53) | 1405.1 (1059.78 to 1803.53) | -0.14 (-0.2 to -0.08) |
| Liberia | 1710.05 (1396.22 to 2037.35) | 1350.97 (987.33 to 1771.36) | 2684.74 (2205.44 to 3152.26) | 1394.31 (1022.75 to 1832.94) | 0.12 (0.09 to 0.15) |
| Central African Republic | 1447.35 (1184.76 to 1731.09) | 1366.66 (1004.62 to 1792.83) | 2527.39 (2084.43 to 3037.44) | 1388.6 (1021.6 to 1811.65) | 0.06 (0.05 to 0.07) |
| Congo | 1494.25 (1218.22 to 1790.77) | 1387.55 (1022.9 to 1826.72) | 3328.48 (2737.61 to 3967.66) | 1380.68 (1021.21 to 1804.53) | -0.05 (-0.06 to -0.04) |
| Guinea-Bissau | 542.97 (441.03 to 647.29) | 1329.92 (976.19 to 1745.43) | 921.61 (754.54 to 1089.64) | 1372.71 (1005.58 to 1791.23) | 0.13 (0.11 to 0.15) |
| Netherlands | 55313.45 (48325.61 to 62794.07) | 2130.17 (1703.81 to 2613.03) | 60517.23 (50715.12 to 70453.15) | 1367.47 (1028.62 to 1753.42) | -1.96 (-2.11 to -1.81) |
| Croatia | 15120.54 (13397.12 to 17066.35) | 1966.86 (1584.4 to 2390.1) | 16411.82 (14402.75 to 18574.46) | 1352.28 (1063.86 to 1674.94) | -1.34 (-1.53 to -1.16) |
| Mauritania | 1442.43 (1176.55 to 1722.81) | 1312.59 (962.56 to 1714.21) | 3031.63 (2497.89 to 3605.63) | 1342.91 (985.94 to 1769) | 0.03 (0 to 0.05) |
| Senegal | 4672.95 (3854.11 to 5536.7) | 1368.73 (1015.81 to 1786.39) | 10485.87 (8763.59 to 12300.63) | 1336.42 (1002.1 to 1722.19) | -0.11 (-0.13 to -0.1) |
| Ghana | 7728.39 (6393.43 to 9102.93) | 1274.4 (943.42 to 1654.17) | 21172.83 (17835.23 to 24811.79) | 1322.81 (1010.16 to 1671.46) | 0.19 (0.16 to 0.22) |
| South Africa | 32960.87 (27204.24 to 39096.75) | 1431.26 (1052.71 to 1869.8) | 65410.6 (54166.54 to 77467.84) | 1318.82 (981.67 to 1714.22) | -0.35 (-0.41 to -0.3) |
| Finland | 21798.63 (19127.37 to 24691.78) | 2320.88 (1859.24 to 2848.42) | 21620.33 (18356.83 to 24917.45) | 1312.96 (1006.29 to 1669.7) | -2.38 (-2.53 to -2.23) |
| Togo | 1551.93 (1268.06 to 1852.6) | 1294.51 (953.14 to 1703.67) | 4455.79 (3709.66 to 5295.58) | 1312.9 (972.44 to 1711.11) | 0.03 (0.01 to 0.06) |
| Sao Tome and Principe | 90.8 (73.67 to 108.7) | 1222.22 (900.62 to 1608.09) | 134.97 (110.63 to 160.22) | 1293.93 (942.21 to 1693.65) | 0.19 (0.16 to 0.22) |
| Italy | 189882.63 (158446.22 to 222259.28) | 1603.51 (1191.15 to 2084.51) | 235147.2 (202536.5 to 269117.76) | 1290.7 (1010.58 to 1616.98) | -0.87 (-1 to -0.73) |
| Eswatini | 365.98 (295.39 to 439.71) | 1267.14 (925.53 to 1670.67) | 764.54 (621.24 to 914.36) | 1287.85 (939.61 to 1687.84) | 0.1 (0.06 to 0.15) |
| Botswana | 748.08 (611.76 to 901.63) | 1251.05 (910.65 to 1652.33) | 1704.3 (1386.57 to 2031.31) | 1274.74 (934.46 to 1659.62) | 0.09 (0.07 to 0.11) |
| Gabon | 766.32 (623.04 to 927.89) | 1240.32 (904.54 to 1641.48) | 1345.93 (1100.5 to 1590.4) | 1272.52 (927.51 to 1672.28) | 0.14 (0.12 to 0.16) |
| Chad | 3711.69 (3043.2 to 4436.78) | 1169.46 (857.15 to 1529.58) | 7083.57 (5900.35 to 8316.46) | 1271.17 (942.29 to 1666.33) | 0.28 (0.27 to 0.3) |
| Angola | 4610.26 (3754.03 to 5441.81) | 1325.71 (972.85 to 1730.44) | 12618.8 (10433.28 to 14984.19) | 1267.73 (944.33 to 1646.28) | -0.22 (-0.26 to -0.19) |
| Democratic Republic of the Congo | 20297.71 (16982.03 to 23815.63) | 1338.39 (1018.45 to 1695.07) | 42110.12 (35963.31 to 48691.03) | 1264.21 (983.55 to 1579.72) | -0.27 (-0.29 to -0.24) |
| Germany | 473294.15 (433710.91 to 515217.11) | 2793.26 (2423.95 to 3199.81) | 327613.05 (295040.03 to 363449.04) | 1258.49 (1024.09 to 1517.54) | -3.03 (-3.22 to -2.84) |
| Burundi | 3049.58 (2498.99 to 3628.5) | 1213.85 (890.92 to 1586.69) | 5347.33 (4398.02 to 6383.85) | 1245.93 (915.37 to 1634.33) | 0.03 (0.01 to 0.06) |
| Guinea | 4129.03 (3397.68 to 4911.06) | 1112.03 (815.87 to 1455.64) | 7238.86 (6032.84 to 8480.56) | 1243.84 (927.63 to 1614.11) | 0.48 (0.42 to 0.54) |
| Mozambique | 7425.61 (6089.35 to 8831.67) | 1240.04 (910.62 to 1623.37) | 13338.05 (11114.5 to 15765.62) | 1243.06 (931.55 to 1603.52) | 0.07 (0.04 to 0.1) |
| Mali | 5119.88 (4178.74 to 6046.43) | 1212.39 (896.85 to 1581.74) | 10676.71 (8855.85 to 12514.98) | 1237.65 (928.57 to 1601.55) | 0.07 (0.03 to 0.11) |
| Djibouti | 133.92 (109.52 to 161.99) | 1146.79 (835.98 to 1510.02) | 661 (536.32 to 794.13) | 1236.02 (899.67 to 1633.08) | 0.23 (0.2 to 0.27) |
| Benin | 2716.99 (2237.52 to 3213.04) | 1228.57 (905.2 to 1610.46) | 6005.75 (4984.32 to 7064.34) | 1235.04 (911.3 to 1607.88) | 0.01 (-0.01 to 0.03) |
| Niger | 3188.34 (2612.61 to 3797.97) | 1211.52 (894.2 to 1585.01) | 9223.1 (7635.53 to 10869.59) | 1234.34 (919.48 to 1594.59) | 0.03 (0 to 0.06) |
| Lesotho | 1236.78 (1010.9 to 1477.68) | 1145.48 (844.39 to 1495.35) | 1621.62 (1319.2 to 1935.95) | 1231.29 (898.67 to 1611) | 0.28 (0.23 to 0.33) |
| Namibia | 1067.91 (869.17 to 1287.02) | 1295.68 (951.55 to 1713.39) | 1846.29 (1510.49 to 2183.8) | 1220.54 (900.17 to 1593.51) | -0.23 (-0.27 to -0.2) |
| Madagascar | 6222.64 (5130.53 to 7276.66) | 1172.89 (870.81 to 1518.09) | 11672.89 (9664.62 to 13839.1) | 1218.31 (912.6 to 1564.8) | 0.1 (0.05 to 0.15) |
| Burkina Faso | 5321.21 (4389.3 to 6306.04) | 1164.11 (862.81 to 1517.81) | 10888.37 (9114.71 to 12677.3) | 1217.24 (914.19 to 1551.22) | 0.16 (0.11 to 0.22) |
| Somalia | 2515.46 (2054.58 to 3002.84) | 1182.93 (865.96 to 1537.83) | 7404.86 (6107.03 to 8800.1) | 1213.03 (897.72 to 1575.97) | 0.08 (0.06 to 0.09) |
| Cabo Verde | 357.63 (291.89 to 422.81) | 1174.87 (860.47 to 1544.14) | 571.48 (471.45 to 680.89) | 1207.18 (886.36 to 1588.96) | 0.05 (0.03 to 0.07) |
| Equatorial Guinea | 258.67 (211.28 to 309.41) | 1332.92 (972.93 to 1754.63) | 559.39 (455.33 to 671.91) | 1203.28 (879.45 to 1577.96) | -0.45 (-0.49 to -0.4) |
| Malawi | 4800.7 (3964.51 to 5710.38) | 1227.76 (907.53 to 1608.48) | 8943.37 (7386.8 to 10625.26) | 1202.36 (886.01 to 1559.28) | -0.13 (-0.16 to -0.09) |
| Nigeria | 52422.92 (43313.01 to 62119.94) | 1132.43 (839.29 to 1472.07) | 98863.7 (81865.32 to 117014.65) | 1193.61 (882.99 to 1552.39) | 0.11 (0.04 to 0.18) |
| South Sudan | 2783.9 (2280.45 to 3314.06) | 1094.9 (802.42 to 1437.24) | 4286.32 (3508.28 to 5092.42) | 1193.01 (877.39 to 1558.03) | 0.27 (0.22 to 0.31) |
| Comoros | 302.9 (244.88 to 361.85) | 1225.49 (893.86 to 1618.98) | 638.35 (526.08 to 756.09) | 1192.68 (874.57 to 1561.45) | -0.15 (-0.17 to -0.13) |
| Vanuatu | 79.29 (64.5 to 94.93) | 1222.73 (896.87 to 1613.81) | 216.49 (176.02 to 256.91) | 1192.53 (873.24 to 1559.51) | -0.1 (-0.13 to -0.08) |
| Cameroon | 4794.54 (3944.72 to 5685.28) | 1090.07 (802.37 to 1431.08) | 13674.23 (11374.39 to 16063.34) | 1189.06 (893.84 to 1528.19) | 0.33 (0.25 to 0.41) |
| United Republic of Tanzania | 12427.45 (10251.7 to 14734.81) | 1090.24 (807.31 to 1420.81) | 29383.23 (24854.62 to 33949.58) | 1182.06 (899.78 to 1497.77) | 0.29 (0.27 to 0.31) |
| Greenland | 50.49 (41.4 to 60.05) | 1569.8 (1140.05 to 2069.57) | 90.18 (73.44 to 107.64) | 1181.28 (862.03 to 1552.45) | -0.94 (-1.01 to -0.88) |
| Iceland | 574.77 (473.11 to 681.91) | 1563.52 (1145.1 to 2058.09) | 822.49 (676.75 to 970.72) | 1173.75 (854.48 to 1546.6) | -0.96 (-1.03 to -0.88) |
| Philippines | 37621.62 (31046.96 to 44682.07) | 1220.76 (900.87 to 1595.41) | 93980.83 (78062.26 to 110708.13) | 1144.87 (851.1 to 1485.04) | -0.34 (-0.45 to -0.23) |
| Honduras | 2304.03 (1906.21 to 2749.19) | 1030.05 (752.14 to 1345.33) | 7581.02 (6311.66 to 8909.64) | 1134.55 (849.92 to 1466.02) | 0.32 (0.27 to 0.37) |
| Kenya | 9798.64 (8058.2 to 11731.98) | 1140.46 (840.73 to 1496.26) | 24112.75 (19734.09 to 28782.44) | 1129.28 (831.79 to 1477.87) | -0.05 (-0.06 to -0.04) |
| Fiji | 369.8 (302.23 to 440.09) | 1140.09 (829.63 to 1498.39) | 848.55 (700.16 to 1007.69) | 1125.21 (822.98 to 1483.07) | -0.08 (-0.12 to -0.03) |
| Solomon Islands | 136.22 (110.28 to 163.49) | 1138.35 (834.36 to 1484) | 294.05 (241.65 to 350.23) | 1121.75 (824.3 to 1473.95) | -0.11 (-0.17 to -0.06) |
| Austria | 25698.92 (22926.37 to 28856.4) | 1593.47 (1306.77 to 1915.21) | 25706.47 (22462.69 to 29321.31) | 1115.34 (879.53 to 1380.27) | -1.5 (-1.59 to -1.4) |
| Zambia | 3233.14 (2626.49 to 3878.64) | 1160 (849.46 to 1526.49) | 7013.9 (5829.09 to 8288.58) | 1110.02 (818.12 to 1456.62) | -0.26 (-0.32 to -0.2) |
| Papua New Guinea | 1801.61 (1467.38 to 2148.18) | 1054.49 (771.66 to 1381.02) | 4529.74 (3750.21 to 5350.72) | 1108.09 (819.4 to 1442.6) | 0.21 (0.17 to 0.25) |
| Denmark | 20503.94 (19063.53 to 22022.39) | 1901.32 (1674.63 to 2138.72) | 16556.49 (13699 to 19739.71) | 1099.81 (819.19 to 1439.68) | -2.02 (-2.12 to -1.91) |
| Venezuela (Bolivarian Republic of) | 13735.46 (11813.5 to 15686.48) | 1302.25 (1011.02 to 1636.01) | 36847.99 (31653.92 to 42335.57) | 1098.18 (863.88 to 1365.16) | -0.75 (-0.83 to -0.67) |
| Nicaragua | 1627.06 (1340.87 to 1947.21) | 1000.76 (736.62 to 1310.63) | 5175.21 (4324.94 to 6093.79) | 1083.34 (806.41 to 1405.2) | 0.25 (0.15 to 0.35) |
| Kiribati | 36.93 (30.07 to 44.18) | 1059.15 (772.96 to 1395.22) | 66.38 (54.17 to 78.94) | 1083.12 (789.82 to 1426) | 0.07 (0.05 to 0.09) |
| Ireland | 10649.76 (9075.39 to 12251.37) | 1966.94 (1534.69 to 2452.68) | 10209.37 (8480.01 to 11950.11) | 1069.78 (787.78 to 1394.39) | -2.65 (-2.86 to -2.44) |
| Samoa | 103.36 (84.14 to 122.92) | 1080.73 (787.42 to 1418.41) | 165.65 (135.38 to 196.31) | 1065.18 (776.72 to 1401.45) | -0.09 (-0.11 to -0.07) |
| Tonga | 64.41 (53.11 to 76.7) | 1076.78 (785.8 to 1416.15) | 98.1 (80.35 to 117.21) | 1062.81 (774.28 to 1400.58) | -0.09 (-0.11 to -0.08) |
| Eritrea | 856.73 (697.32 to 1025.98) | 1075.67 (784.55 to 1413.57) | 2469.02 (2025.38 to 2959.66) | 1061.81 (778.54 to 1400.15) | -0.06 (-0.08 to -0.05) |
| American Samoa | 22.31 (18.29 to 26.77) | 1022.65 (749.01 to 1350.68) | 54.42 (44.59 to 64.62) | 1059.54 (774.55 to 1393.27) | 0.11 (0.1 to 0.12) |
| China | 771963.29 (653713.8 to 892171.57) | 933.11 (714.17 to 1193.51) | 2406500.27 (2052074.88 to 2766416.28) | 1058.74 (816.38 to 1343.91) | 0.75 (0.62 to 0.89) |
| Tokelau | 1.82 (1.48 to 2.16) | 1021.97 (743.06 to 1352.68) | 1.6 (1.29 to 1.91) | 1051.13 (762.7 to 1390.79) | 0.06 (0.04 to 0.09) |
| Ethiopia | 19834.09 (16311.86 to 23658.82) | 1032.11 (765.52 to 1344.74) | 44266.36 (36796.38 to 52128.46) | 1046.24 (782.82 to 1360.5) | -0.03 (-0.09 to 0.02) |
| Marshall Islands | 16.5 (13.36 to 19.78) | 994 (727.57 to 1307.2) | 31.79 (25.66 to 38.17) | 1045.93 (756.11 to 1382.11) | 0.16 (0.14 to 0.18) |
| Tuvalu | 7.46 (6.06 to 8.91) | 1015.55 (741.85 to 1340.89) | 12 (9.84 to 14.3) | 1043.2 (761 to 1379.07) | 0.08 (0.07 to 0.09) |
| Nauru | 2.94 (2.39 to 3.47) | 1048.23 (762.72 to 1380.25) | 2.8 (2.3 to 3.35) | 1042.22 (765.19 to 1362.16) | -0.02 (-0.11 to 0.06) |
| Belgium | 35402.91 (30693.66 to 40677.83) | 1713.81 (1353.28 to 2123.94) | 31013.46 (26050.1 to 36180.9) | 1041.49 (778.05 to 1343.02) | -1.93 (-2.05 to -1.81) |
| Palau | 10.93 (8.89 to 13.03) | 1020.23 (742.18 to 1345.04) | 21.65 (17.6 to 25.88) | 1029.26 (750.04 to 1354.64) | 0 (-0.02 to 0.01) |
| Mexico | 52213.95 (43320.63 to 61831.51) | 1108.37 (819.74 to 1440.73) | 138546.06 (115437.35 to 163060.68) | 1025.62 (761.03 to 1330.84) | -0.36 (-0.4 to -0.32) |
| Uruguay | 6668.23 (5628.96 to 7742.42) | 1314.27 (989.79 to 1683.06) | 7316.77 (6073.22 to 8647.12) | 1012.76 (747.79 to 1322.99) | -1.11 (-1.21 to -1.01) |
| Cook Islands | 13.95 (11.38 to 16.68) | 1015.8 (739.72 to 1337.43) | 31.1 (25.38 to 36.98) | 1012.48 (737.62 to 1335.43) | -0.05 (-0.06 to -0.04) |
| Uganda | 7027.05 (5813.07 to 8327.58) | 1041.64 (776.78 to 1356.8) | 13802.85 (11412.63 to 16197.89) | 1012.04 (755.68 to 1307.28) | -0.16 (-0.21 to -0.11) |
| Greece | 29591.58 (25624.06 to 33623.09) | 1485.38 (1172.09 to 1852.99) | 33115.4 (28329.4 to 37852.22) | 1011.26 (775.67 to 1272.09) | -1.61 (-1.72 to -1.49) |
| Niue | 2.8 (2.32 to 3.35) | 987.29 (718.15 to 1293.5) | 2.68 (2.2 to 3.19) | 1002.9 (729.01 to 1324.05) | 0.01 (-0.01 to 0.04) |
| Canada | 79705.17 (70713.43 to 89782.9) | 1922.95 (1570.95 to 2316.67) | 88449.06 (76076.11 to 101572.28) | 993.86 (761.12 to 1249.22) | -2.56 (-2.72 to -2.4) |
| Norway | 13719.9 (11347.78 to 16206.78) | 1470.94 (1092.32 to 1912.54) | 12149.7 (9952.86 to 14405.78) | 993.4 (731.09 to 1303.45) | -1.68 (-1.83 to -1.52) |
| Northern Mariana Islands | 11.97 (9.66 to 14.42) | 954.19 (694.74 to 1266.61) | 49.92 (40.64 to 59.67) | 990.06 (720.31 to 1308.12) | 0.1 (0.07 to 0.12) |
| Guam | 66.05 (54.06 to 79.5) | 885.42 (645.1 to 1172.47) | 218.65 (180.15 to 260.94) | 988.55 (721.6 to 1301.94) | 0.39 (0.34 to 0.43) |
| Rwanda | 3062.78 (2479.77 to 3680.34) | 1032.62 (755.86 to 1353.2) | 5865.75 (4808.71 to 6989.43) | 980.62 (723.13 to 1286.49) | -0.31 (-0.36 to -0.26) |
| Chile | 15087.29 (12818.9 to 17353.51) | 1321.99 (1016.81 to 1675.03) | 29217.26 (24560.07 to 34532.48) | 979.31 (733.81 to 1272.56) | -1.3 (-1.65 to -0.94) |
| Micronesia (Federated States of) | 50.37 (40.39 to 60.78) | 1038.62 (750.88 to 1373.22) | 64.47 (52.9 to 77.31) | 979.09 (714.24 to 1286.82) | -0.24 (-0.27 to -0.21) |
| Malaysia | 9775.22 (8381.22 to 11283.31) | 1020.52 (780.02 to 1285.25) | 28984.63 (25000.25 to 33245.89) | 977.46 (764.38 to 1220.03) | -0.52 (-0.73 to -0.31) |
| Andorra | 82.8 (66.88 to 99.49) | 1238.25 (901.63 to 1631.77) | 163.51 (132.92 to 195.03) | 976.38 (709.34 to 1296.72) | -1.11 (-1.23 to -0.98) |
| San Marino | 55.08 (44.77 to 65.63) | 1265.77 (928.85 to 1670.86) | 79.4 (64.9 to 94.6) | 973.65 (709.56 to 1281.86) | -1.06 (-1.18 to -0.93) |
| Democratic People's Republic of Korea | 12485.11 (10501.58 to 14444.27) | 871.21 (657.69 to 1122.59) | 35521.29 (31006.77 to 40201.58) | 967.18 (769.54 to 1195.05) | 0.43 (0.33 to 0.52) |
| Monaco | 123.06 (101.38 to 146.8) | 1257.95 (922.9 to 1642.29) | 119.66 (98.15 to 142.27) | 962.91 (703.63 to 1264.99) | -1.09 (-1.2 to -0.98) |
| Sweden | 34266.32 (31735.58 to 37150.93) | 1629.9 (1422.79 to 1858.52) | 26912.34 (23588.68 to 30509.12) | 960.12 (763.89 to 1178.58) | -2.11 (-2.24 to -1.97) |
| United States of America | 895962.41 (742059.59 to 1058423.46) | 2123.33 (1560.1 to 2776.04) | 683929.36 (606483.7 to 770735.84) | 952.79 (778.23 to 1150.58) | -3.08 (-3.23 to -2.93) |
| United Kingdom | 193227.63 (158552.45 to 228538.38) | 1570.57 (1160.38 to 2056.92) | 154630.96 (130907.83 to 178151) | 951.23 (729.24 to 1209.36) | -1.9 (-2.09 to -1.71) |
| Costa Rica | 2063.12 (1716.81 to 2444.49) | 1028.58 (757.45 to 1339.03) | 5784.86 (4776.74 to 6862.02) | 947.9 (694.4 to 1238.94) | -0.38 (-0.41 to -0.35) |
| Guatemala | 3424.84 (2841.43 to 4047.86) | 922.01 (682.51 to 1200.54) | 12157.79 (10262.63 to 14220.61) | 937.52 (700.57 to 1209.76) | 0.04 (-0.03 to 0.11) |
| El Salvador | 3052.87 (2521.27 to 3612.64) | 911.31 (673.65 to 1187.7) | 6785.23 (5640.27 to 7965.56) | 934.98 (690.22 to 1214.29) | 0.05 (0.02 to 0.09) |
| Panama | 1612.27 (1330.02 to 1915.9) | 946.32 (690.93 to 1239.91) | 4589.5 (3803.13 to 5480.83) | 929.66 (682.88 to 1220.88) | -0.08 (-0.09 to -0.06) |
| Spain | 103977.48 (91218.11 to 117291.61) | 1447.06 (1174.79 to 1761.48) | 111523.16 (96012.37 to 127641.54) | 915.91 (698.4 to 1160.81) | -1.73 (-1.87 to -1.59) |
| Switzerland | 20035.33 (17127.68 to 22978.73) | 1437.69 (1113.3 to 1793.16) | 20301.75 (16978.72 to 23758.52) | 911.29 (676.76 to 1180.13) | -1.52 (-1.71 to -1.33) |
| Colombia | 22583.43 (19437.46 to 25901.02) | 1211.27 (952.63 to 1509.05) | 57107.19 (49123.58 to 65672.41) | 893.55 (688.08 to 1126.52) | -1.19 (-1.27 to -1.12) |
| Singapore | 2750.36 (2257.97 to 3223.88) | 1185.99 (875.01 to 1548.9) | 8186.54 (7660.11 to 8737.98) | 889.46 (790.99 to 996.21) | -0.77 (-0.91 to -0.63) |
| Argentina | 56942.86 (50904.18 to 63879.47) | 1460.47 (1209.23 to 1733.34) | 61270.43 (53521.92 to 69725.45) | 889.3 (701.32 to 1099.68) | -1.92 (-2.11 to -1.74) |
| Israel | 10030.3 (8461.22 to 11601.75) | 1571.26 (1200.91 to 1977.74) | 13125 (10860.75 to 15421.03) | 885.29 (646.23 to 1168.91) | -2.43 (-2.68 to -2.17) |
| France | 165475.84 (146693.54 to 185834.74) | 1466.38 (1177.87 to 1778.15) | 155786.06 (133843.58 to 178472.48) | 874.8 (664.09 to 1117.54) | -2.07 (-2.18 to -1.96) |
| Brunei Darussalam | 82.98 (67.95 to 99.24) | 963.63 (706.01 to 1269.92) | 223.4 (181.46 to 266.61) | 832.43 (609.58 to 1095.74) | -0.59 (-0.64 to -0.55) |
| Taiwan (Province of China) | 15785.81 (13207.7 to 18431.27) | 984.94 (742.74 to 1268.24) | 41004.41 (34140.24 to 48129.2) | 831.06 (616.65 to 1084.8) | -0.55 (-0.65 to -0.45) |
| Sri Lanka | 11554.95 (9913.34 to 13342.77) | 1024.35 (795.99 to 1285.38) | 25125.08 (21501.59 to 29026.23) | 829.81 (642.15 to 1050.42) | -0.83 (-0.94 to -0.71) |
| Malta | 603.92 (499.29 to 717.73) | 1125.54 (827.22 to 1476.14) | 1019.14 (839.17 to 1210.32) | 816.76 (598.26 to 1064.6) | -0.92 (-1.3 to -0.54) |
| Lao People's Democratic Republic | 1828.93 (1503.12 to 2178.3) | 839.22 (619.04 to 1098.73) | 3502.5 (2910.68 to 4139.33) | 801.23 (593.14 to 1040.16) | -0.2 (-0.28 to -0.12) |
| Luxembourg | 728 (602.24 to 863.2) | 1012.69 (750.94 to 1327.73) | 978.1 (802.91 to 1163.44) | 786.12 (572.29 to 1031.93) | -1.02 (-1.37 to -0.67) |
| Myanmar | 22399.42 (19255.49 to 25803.04) | 927.71 (724.6 to 1163.27) | 38613.01 (33328 to 44159.14) | 785.47 (618.08 to 970.74) | -0.76 (-0.83 to -0.69) |
| Viet Nam | 36433.86 (31536.18 to 41947.67) | 798.1 (631.43 to 993.84) | 75894.64 (66774.82 to 85569.14) | 771.69 (626.09 to 930.74) | -0.19 (-0.25 to -0.14) |
| Timor-Leste | 184.25 (148.87 to 222.18) | 768.65 (558.73 to 1011.06) | 723.95 (592.19 to 867.31) | 759.14 (554.12 to 995.49) | -0.05 (-0.06 to -0.03) |
| Cambodia | 3564.37 (2950.34 to 4182.97) | 784.77 (583.8 to 1017.89) | 9386.61 (7925.05 to 11029.65) | 749.82 (561.37 to 970.67) | -0.18 (-0.23 to -0.14) |
| Maldives | 74.34 (59.77 to 89.8) | 873.91 (635.83 to 1156.08) | 222.9 (183.92 to 265.26) | 746.88 (547.21 to 977.92) | -0.66 (-0.72 to -0.61) |
| Seychelles | 53.99 (44.29 to 64.76) | 763.2 (555.97 to 1008.56) | 90.46 (73.97 to 108.29) | 742.05 (539.84 to 984.48) | -0.12 (-0.13 to -0.1) |
| Mauritius | 719.69 (593.26 to 848.16) | 871.52 (645.68 to 1125.51) | 1555.52 (1272.5 to 1874.5) | 739.03 (539.99 to 981.74) | -0.9 (-1.01 to -0.8) |
| Cyprus | 982.96 (806.43 to 1173.54) | 956.54 (710.92 to 1253.51) | 1843.77 (1505.57 to 2184.41) | 725.79 (531.55 to 951.72) | -1.58 (-1.83 to -1.33) |
| Poland | 104417.65 (86542.63 to 122794.76) | 1932.5 (1431.57 to 2521.73) | 64507.14 (57995.46 to 71453.99) | 688.87 (571.71 to 818.97) | -4.28 (-4.71 to -3.85) |
| Japan | 171417 (142647.14 to 201271.67) | 819.72 (608.72 to 1070.9) | 325585.83 (268225.58 to 386977.87) | 671.01 (495.32 to 879.93) | -1.01 (-1.18 to -0.85) |
| Thailand | 26288.41 (22157.56 to 30506.16) | 739 (565.45 to 937.79) | 73227.83 (61669 to 85056.37) | 594.72 (451.92 to 756.76) | -0.96 (-1.04 to -0.89) |
| Brazil | 66172.05 (56258.93 to 76759.42) | 684.83 (527.66 to 862.51) | 167065.2 (144191.01 to 192486.87) | 589.51 (462.38 to 733.34) | -0.46 (-0.65 to -0.27) |
| Republic of Korea | 28620.7 (24753.27 to 32445.67) | 1035.88 (823.27 to 1270.18) | 61398.22 (51575.41 to 71506.78) | 550.96 (413.29 to 716.69) | -2.76 (-3.14 to -2.38) |
| Paraguay | 1333.35 (1097.76 to 1581.5) | 528.91 (386.15 to 690.45) | 3349.94 (2750.01 to 3962.91) | 527.13 (388.89 to 686.1) | -0.01 (-0.08 to 0.05) |
| Bolivia (Plurinational State of) | 1691.21 (1394.87 to 1987.99) | 503.07 (372.03 to 653.86) | 5004.23 (4149.65 to 5841.14) | 511.56 (381.43 to 666.25) | 0 (-0.14 to 0.14) |
| Ecuador | 2648.28 (2227.23 to 3102.44) | 445.08 (335.45 to 573.35) | 7903.5 (6695.28 to 9141.17) | 446.92 (345.25 to 563.43) | -0.07 (-0.32 to 0.17) |
| Indonesia | 50242.13 (40468.46 to 60276.37) | 527.19 (382.37 to 697.7) | 98229.93 (82871.37 to 115472.58) | 442.76 (336.14 to 566.25) | -1.03 (-1.5 to -0.56) |
| Portugal | 15775.87 (13583.62 to 18161.87) | 874.66 (677.3 to 1089.85) | 14009.79 (11784.48 to 16429.14) | 431.67 (324.5 to 554.05) | -3.03 (-3.28 to -2.77) |
| Peru | 6384.06 (5362.21 to 7408.02) | 496.72 (374.22 to 639.63) | 15387.84 (12699.47 to 18043.35) | 404.67 (298.14 to 528.17) | -0.9 (-1.06 to -0.73) |
